# Microplastics enhance the prevalence of antibiotic resistance genes in mariculture sediments by enriching host bacteria and promoting horizontal gene transfer

**DOI:** 10.1016/j.eehl.2025.100136

**Published:** 2025-01-30

**Authors:** Yifan Liu, Liuqingqing Liu, Xiao Wang, Mengying Shao, Zihan Wei, Lina Wang, Bing Li, Chenguang Li, Xianxiang Luo, Fengmin Li, Hao Zheng

**Affiliations:** a Institute of Coastal Environmental Pollution Control, College of Environmental Science and Engineering, Key Laboratory of Marine Environment and Ecology, Ministry of Education, Frontiers Science Center for Deep Ocean Multispheres and Earth System, Ocean University of China, Qingdao 266100, China; b Sanya Oceanographic Institution, Ocean University of China, Sanya 572000, China; c Marine Agriculture Research Center, Tobacco Research Institute, Chinese Academy of Agricultural Sciences, Qingdao 266101, China; d School of Energy and Environmental Engineering, University of Science and Technology Beijing, Beijing 100083, China

**Keywords:** Antimicrobial resistance, Microbial community, Horizontal gene transfer, Plastisphere, Biofilm colonization, Plastic additives

## Abstract

Microplastics (MPs) and antibiotic resistance genes (ARGs) pose significant challenges to the One Health framework due to their intricate and multifaceted ecological and environmental impacts. However, the understanding of how MP properties influence ARG prevalence in mariculture sediments remains limited. Herein, the polystyrene (PS) and polyvinyl chloride (PVC) MPs with different sizes (20–120 μm and 0.5–2.0 mm) were selected to evaluate their impacts and underlying mechanisms driving ARGs dissemination. The results showed that PS and PVC MPs increased the relative abundance of ARGs by 1.41–2.50-fold and 2.01–2.84-fold, respectively, compared with control, particularly high-risk genes. The polymer type effect was identified as more influential than the size effect in driving the sediment resistome evolution. PVC shifted the microbial community assembly from stochastic to deterministic processes, thus enriching ARG host pathogens. Furthermore, the highly hydrophobic PS not only recruited the host bacteria colonization but also facilitated ARG exchange within the plastisphere. The exogenous additives released by PVC (e.g., heavy metals, bisphenol A, and tridecyl ester) and the particles synergistically promoted ARG conjugative transfer by inducing oxidative stress and enhancing cell membrane permeability. These findings revealed how MPs characteristics facilitated the spread of ARGs in marine benthic ecosystems, underscoring the importance of mitigating MPs pollution to maintain mariculture ecosystem health, prevent zoonotic diseases, and balance global mariculture with ecological health.

## Highlights

• Polymer type- and size-dependent elevation of ARGs abundance in mariculture sediments are investigated.
• MPs recruit ARBs by enhancing deterministic assembly of bacterial community.
• Hydrophobic PS MPs facilitate bacterial contact to promote plastisphere formation.
• PVC MPs enhance HGT by triggering oxidative stress-mediated membrane permeability.
• PVC MPs particles and endogenous additives synergistically enhance ARGs transfer.

## Graphical abstract

**Figure:**
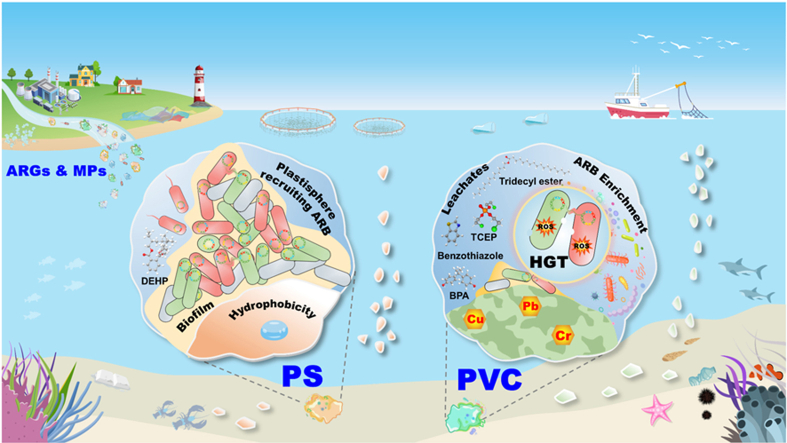

## 1 Introduction

Antimicrobial resistance (AMR), a priority environmental and health concern from a One Health perspective [1], has rapidly emerged and spread globally through the occurrence and dissemination of antibiotic resistance genes (ARGs) [2]. Aquatic products from mariculture, an essential component of the global food system, contribute to 12%–25% of the newly added human nutrition demand for high-quality protein [3]. However, the improper or abusive use of antibiotics in the aquaculture industry extremely exacerbated ARG pollution in mariculture ecosystems [4]. More than 50% of mariculture environments and aquatic products worldwide are facing AMR issues [5]. The ARG abundances detected in typical mariculture sediments (e.g., Bohai Sea, Baltic Sea, and Caulín Bay) were as high as 4.67 × 10^3^–1.08 × 10^7^ copies/g, two to four orders of magnitude higher than those in the surrounding seawaters [6]. Thus, understanding the dissemination processes of ARGs in mariculture sediments is crucial to safeguarding human health and ensuring sustainable aquaculture practices.

Microplastics (MPs) are also widely distributed in mariculture environments and have emerged as one of the global environmental issues threatening the health and stability of marine ecosystems [7,8]. Approximately 99% of MPs from fishing, offshore operations, and land-based discharges into the ocean enter sediments and have intensified MP burdens in sediments [9,10]. The abundance of MPs in major coastal mariculture sediments worldwide has reached between 5.98 × 10^2^ and 1.48 × 10^5^ particles/kg, one to three orders of magnitude higher than those of non-aquaculture environments with an average abundance of 24–84 particles/kg [11]. The interactions between MPs and ARGs in various environments have become a significant focus of research due to their potential impact on global health and ecosystems [12,13]. Currently, most studies emphasize the role of the plastisphere associated with floating MPs in seawater as carriers that facilitate ARG transmission and diffusion [14,15]. However, sporadic studies have investigated the effects of MPs on ARG abundance in marine sediments [16,17], and the effects of MPs as coexisting contaminants in marine sediment microbiomes on ARG distribution are far from being understood. A study found that polyhydroxyalkanoate MPs significantly enriched ARGs in mariculture sediments by increasing the abundance of host bacteria compared to polyethylene terephthalate MPs [16]. Another study observed that polyethylene (PE) and polyvinyl chloride (PVC) MPs increased ARG abundance in mariculture sediments and followed an order of PVC > PE [17]. Inconsistently, in coastal mangrove sediment, ARG abundance on the surface of PE MPs was much higher than that of PVC MPs [18]. Obviously, the effects of different types of MPs on ARG abundance in marine sediments are still uncertain. Specifically, the physicochemical properties of MPs largely dictate their environmental behaviors and ecological impacts [7], inevitably influencing the proliferation and dissemination of ARGs in sediments, yet their roles have been consistently underestimated. Therefore, elucidating the effects and underlying mechanisms of MPs on ARGs dissemination in mariculture ecosystems is a prerequisite for predicting their environmental risks and establishing effective strategies for combating AMR from a One Health perspective.

Microbial communities and horizontal gene transfer (HGT) are critical factors determining the abundance and diversity of ARGs in the environment [19]. Extensive studies evidenced that the coexisting pollutants such as heavy metals, antibiotics, and engineered nanoparticles elevated antibiotic-resistant bacteria (ARB) abundances in aquatic environments by disrupting bacterial community succession and reducing bacterial community diversity [15,20]. Furthermore, HGT is a rampant evolutionary force driving the spread of ARGs among bacterial species [21]. Recently, numerous studies have evidenced that the emerging contaminants like disinfection by-products, perfluorocarbons, and endocrine disrupting chemicals [22] could disrupt bacterial metabolism, induce reactive oxygen species (ROS) overproduction, increase cell membrane permeability, thus improving potential for HGT of ARGs [23,24]. The complex physicochemical properties of MPs, mainly including polymer type, sizes, hydrophobicity, and exogenous additives, are the fundamental factors determining their ecological effects [14]. Particularly, the differences in hydrophobicity of MPs resulting from polymer type and surface functional groups can selectively induce colonization of sediments by specific bacteria like ARB [25]. The exogenous additives [e.g., phthalates, bisphenol A (BPA), and heavy metals] released from MPs could exert co-selection pressure on bacteria, thus driving community succession and inducing oxidative stress to bacteria [14,26]. Moreover, the biofilms formed on the surfaces of particles can also facilitate gene transfer [25]. Therefore, it is reasonable to hypothesize that the pollution of MPs will increase ARG abundances in mariculture sediments and show the polymer type and size-dependent patterns, which can be mainly attributed to the formation of dense plastisphere providing for ARG host bacteria colonization resulting from the hydrophobic MPs, and the enhanced HGT by the released exogenous additives and the increased cellular contact within the plastisphere.

To prove the above hypothesis, two commonly detected MPs derived PS and PVC, which differ in polarity—with PS being non-polar and PVC being more polar—were selected with different particle sizes (20–120 μm and 0.5–2.0 mm) in mariculture environments (Table S1). These MPs were used to investigate their impacts on the abundance and HGT of ARGs in mariculture sediments using microcosm experiments, biofilm dynamics observations, and plasmid conjugation experiments. The specific research objectives are to: 1) elucidate the impact patterns of different polymer types and sizes of MPs on the abundance of ARGs in the mariculture sediments; 2) explore the potential mechanisms underlying the MPs-induced bacterial community succession driving ARB enrichment; 3) evaluate the roles of MP properties in affecting horizontal ARG transfer. These results will first provide insight into the potential mechanisms by which MP properties promote the dissemination of ARGs in mariculture benthic ecosystems.

## 2 Materials and methods

### 2.1 Preparation of MPs and bacterial strains

**Fig. 1:**
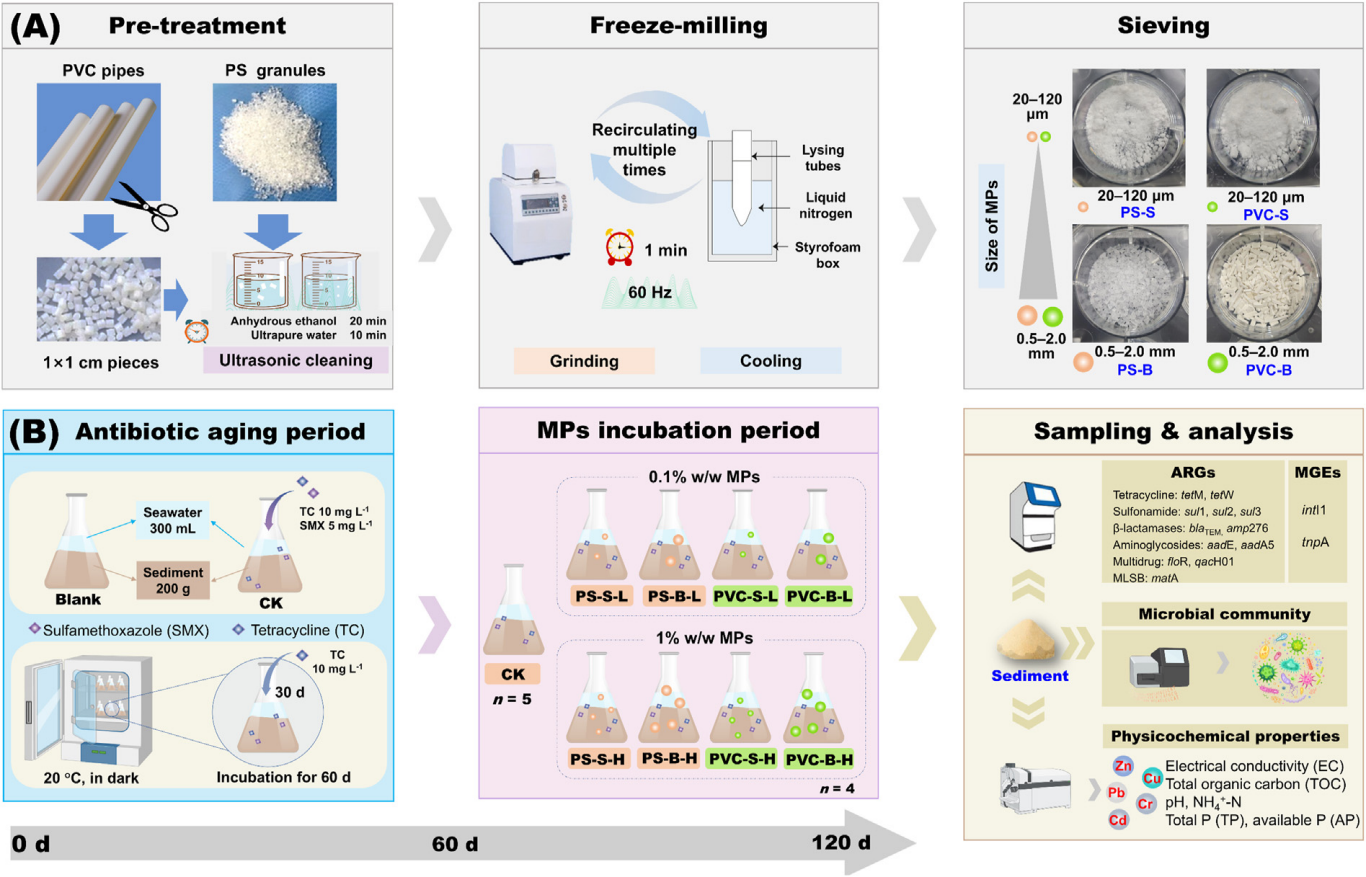
Experimental workflow of this study. (A) MPs preparation; (B) sediment microcosmic experiment design. PS-S (20–120 μm), PVC-S (20–120 μm), PS-B (0.5–2.0 mm) and PVC-B (0.5–2.0 mm) were obtained by freeze-milling and sieving of the purchased PS granules and PVC pipes. The maricultural sediment was aged with two antibiotics (TC and SMX) for 60 days to induce the occurrence of ARGs before adding MPs. Then the MPs were added in to the sediments and the microcosms were cultivated for another 60 days to explore their effects on ARG propagation. Blank group: the sediment without antibiotic addition. CK group: the sediments with antibiotic addition during the antibiotic aging period and without MPs addition during the MPs incubation period. The MPs were added at the low (0.1%, -L) and high (1%, -H) amounts.

PS and PVC MPs, commonly found in mariculture environments (Table S1), were selected in the experiment. Based on our previous study [27] and existing research (Table S1), MPs with sizes of 20–120 μm and 0.5–2.0 mm are dominant in mariculture sediments. Therefore, PS granules were bought from Yangli Technology Co., Ltd. (Shanghai, China), and commercial PVC pipes were bought from Weihai Huana plastic Co., Ltd. (Shandong, China) to prepare the small (20–120 μm, PS-S, PVC-S) and big (0.5–2.0 mm, PS-B, PVC-B) MPs (Fig. 1A). Currently, most studies use particle counts rather than weight, limiting the reference for specific concentrations (Table S1). Therefore, we set the concentration of MPs added in this experiment at 0.1%–1% by weight, based on the abundances used in existing research [7,17]. The details of the preparation and characterization of MPs are presented in the Supplementary Information (SI, Text S1–S3, Figs. S1–S2, Tables S2–S5).

*Pseudomonas putida* (*P. putida*) and *Escherichia coli* (*E. coli*) are representative bacterial strains that harbor significant levels of ARGs in mariculture environments [28,29], making them suitable models for studying the interactions between bacteria and MPs. The *P. putida* KT2440::*lacI*^*q*^-*dsRed*, harboring a conjugative *gfp*-RP4 plasmid, was incubated in Lysogeny broth (LB) medium supplemented with ampicillin (Amp, 100 mg/L) and kanamycin (Km, 50 mg/L) [30]. *E. coli* NK5449 was grown in an LB medium supplemented with rifampicin (Rif, 160 mg/L) [31]. The donor strain (*P. putida* KT2440) and the recipient strain (*E. coli* NK5449) were incubated overnight at 37 °C with appropriate antibiotics. Subsequently, strains were centrifuged separately at 10,000 × *g* for 10 min, washed twice with phosphate-buffered saline (PBS), and diluted in PBS to a concentration of 3 × 10^8^ CFU/mL for subsequent experiments.

### 2.2 Maricultural sediment microcosms

The schematic diagram and details of the microcosmic experiment design were presented in Fig. 1B. It included an antibiotic aging period and an MP incubation period. The details for the preparation and properties of sediments are presented in the Text S4, Table S6. Based on the environmental-related concentrations of tetracycline (TC) and sulfamethoxazole (SMX), the sediment was aged by a mixed solution of TC (10 mg/L) and SMX (5 mg/L) for 60 days to accurately simulate high levels of pollution with antibiotics in aquaculture (Table S7). These designed antibiotic concentrations provide a realistic simulation of the antibiotic selection pressure in mariculture sediments, resulting in a stable antibiotic resistance gene composition. Specifically, microcosms were set up in 500 mL flasks filled with 200 g of air-dried sediment and 300 mL of natural seawater (pH 8.0, salinity 31.6‰) containing TC and SMX. There were 42 conical flasks for the antibiotic treatments and 5 flasks for the blank treatments without antibiotics. For the whole aging period, all the flasks wrapped with aluminum were cultivated at 20 °C in the dark for 60 days. The ARG abundance and concentrations of TC and SMX in the sediments were measured after 60 days of cultivation.

During the MP incubation period, PVC and PS MPs were separately added to the sediments in amounts of 0.1% and 1% (w/w) (Fig. 1B), based on environmentally relevant levels (Table S1). In total, nine treatment groups were set up, consisting of eight groups exposed to the four MPs and one control group without MPs (CK). The groups treated with MPs were designated as follows: PS-S-L, PS-S-H, PS-B-L, PS-B-H, PVC-S-L, PVC-S-H, PVC-B-L and PVC-B-H, where -S and -B labeled the small and large size of MPs, and -L and -H pointed out the relatively lower (0.1%) and higher rate (1%) of the added MPs. Each treatment was set up with four replicates. After 60 days of cultivation, sediments were analyzed for ARG and mobile genetic element (MGE) abundance, microbial communities, and the selected physicochemical properties (Text S5, Fig. S3).

### 2.3 DNA extraction and quantification of the targeted genes

The DNA was extracted from 0.5 g of freeze-dried sediment by Powersoil DNA extraction kit (Qiagen, USA) [32]. Real-time quantitative polymerase chain reaction (qPCR) was performed using an ABI StepOne Plus real-time PCR system (Applied Biosystems, USA) to determine 12 representative ARGs and two MGEs, which are widely detected in mariculture environments worldwide (Fig. 1B, Table S8). More detailed information on the amplification procedure of target genes, standard curves (Table S9), primer sequence, protocols for qPCR reaction systems (Table S10), and quality control were reported in the previous studies [32,33]. The abundance of the 16S rRNA gene was used as the reference gene to calculate the relative abundance of ARGs and MGEs [4].

### 2.4 Sediment microbiome analysis

The microbiome in marine sediments was analyzed by 16S rRNA amplicon sequencing using the Illumina MiSeq technique (Illumina PE250, USA). The bacterial 16S rRNA gene, which specifically targeted the V3–V4 region, was amplified using primers 341F (CCTACGGGNGGCWGCAG) and 806R (GGACTACHVGGGTATCTAAT). Detailed information on the PCR amplification conditions, library preparation, and sequence data processing was provided in a previous study [32].

### 2.5 Observation of biofilm colonization and HGT on the surface of MPs

An optimized biofilm growth experiment was designed using *P. putida* KT2440 and *E. coli* NK5449. In brief, 1.5 mL of a mating mixture of *P. putida* KT2440 and *E. coli* NK5449, containing 1.5 or 15 mg MP, was added into each well of 24-well microplates and then incubated at 20 °C in the dark for 144 h. A Laser scanning confocal microscope (LSCM, Nikon A1Plus, Nikon, Japan) was employed to observe the distribution of green fluorescence (excitation of 488 nm and emission of 525 nm) representing transconjugants and red fluorescence (excitation of 561 nm and emission of 650 nm) representing donor cells at the desired time (1, 12, 24, 48, 72, 96, 120, and 144 h). All fluorescence images were processed and analyzed in ImageJ (Fiji version 1. 52i, National Institute of Health, MD, USA). The transfer frequency was determined as the ratio of the transconjugant area to the donor cell area [30]. Additionally, crystal violet staining and real-time qPCR were used to quantify the biomass of biofilm formed on MPs, and the details are described in the Text S6.

### 2.6 Contributions of MP particles, leachates, and their combined effects on the conjugative transfer of ARGs

An optimized intergeneric conjugative transfer model was established using the donor strain *P. putida* KT2440 and the recipient strain *E. coli* NK5449. Briefly, 150 μL of the resuspended donor and recipient bacterial suspensions were mixed at a ratio of 1:1 and added to 30 mL of LB medium to make a mating mixture. The rinsed MPs (particles without leachates) corresponding leachates (filtering out MPs), and unwashed pristine MPs (particles + leachates) were added to the mating mixture. The concentrations of MPs in the mixture were 0, 1, 10, and 100 mg/L, respectively.

The dosages of leachates were consistent with the corresponding concentrations of MPs. The conjugation mixtures were then cultured at 37 °C and 200 rpm for 18 h [31]. Subsequently, the mixtures were inoculated onto selective LB plates supplemented with the corresponding antibiotics for 24 h to screen transconjugants, donors, and recipients. The plasmid transfer frequency was calculated as the ratio of transconjugant counts to recipient cell counts. The interactive effects of MPs and their leachates on HGT were assessed using the Bliss independence model [34,35], and details were presented in Text S7. Intracellular ROS production and cell membrane permeability of recipients were assessed as described in a previous study [31].

### 2.7 Statistical analysis

One-way analysis of variance (ANOVA) and Student's *t*-test were conducted using Statistical Product and Service Solutions (SPSS) software (Version 25.0, SPSS Inc, Chicago, IL, USA). Analyses on the similarities (ANOSIM), non-metric multidimensional scaling (NMDS), and procrustes analysis were computed using the “vegan 2.6.4” package. Partial least squares-discriminant analysis (PLS-DA) plots were generated using the “mixOmics 6.22.0” package. Network analysis employed the “Hmisc” package and was visualized using Gephi software (Version 0.9.1). The neutral community model (NCM) and null model were implemented using the “Hmisc”, “minpack. lm”, “stats4” and “picante” packages [36]. The structural equation model (SEM) was constructed using AMOS 21 (SPSS Inc., Chicago, IL, USA). Further details are provided in Text S8.

## 3 Results and discussion

### 3.1 MPs elevated ARGs abundance in mariculture sediments

After 60 days of age with the two antibiotics, TC and SMX concentrations in the sediment pore water were 17.3 ng/L and 5.81 ng/L, respectively (Fig. S4). The fluctuation of the antibiotic concentrations aligned with those detected in typical mariculture regions like Jiaozhou Bay and the Baltic Sea (Table S7). Following aging, the absolute abundances of six target ARGs increased from 0.32 × 10^8^ copies/g to 1.56 × 10^8^ copies/g (Fig. S5). These ARG abundances were equivalent to the levels reported in the mariculture sediments from China, Bangladesh, and Thailand, ranging from 10^3^ to 10^10^ copies/g (Table S8). These results proved that the sediment microcosm system receiving antibiotic inputs successfully simulated ARG pollution in the real mariculture environment.

**Fig. 2:**
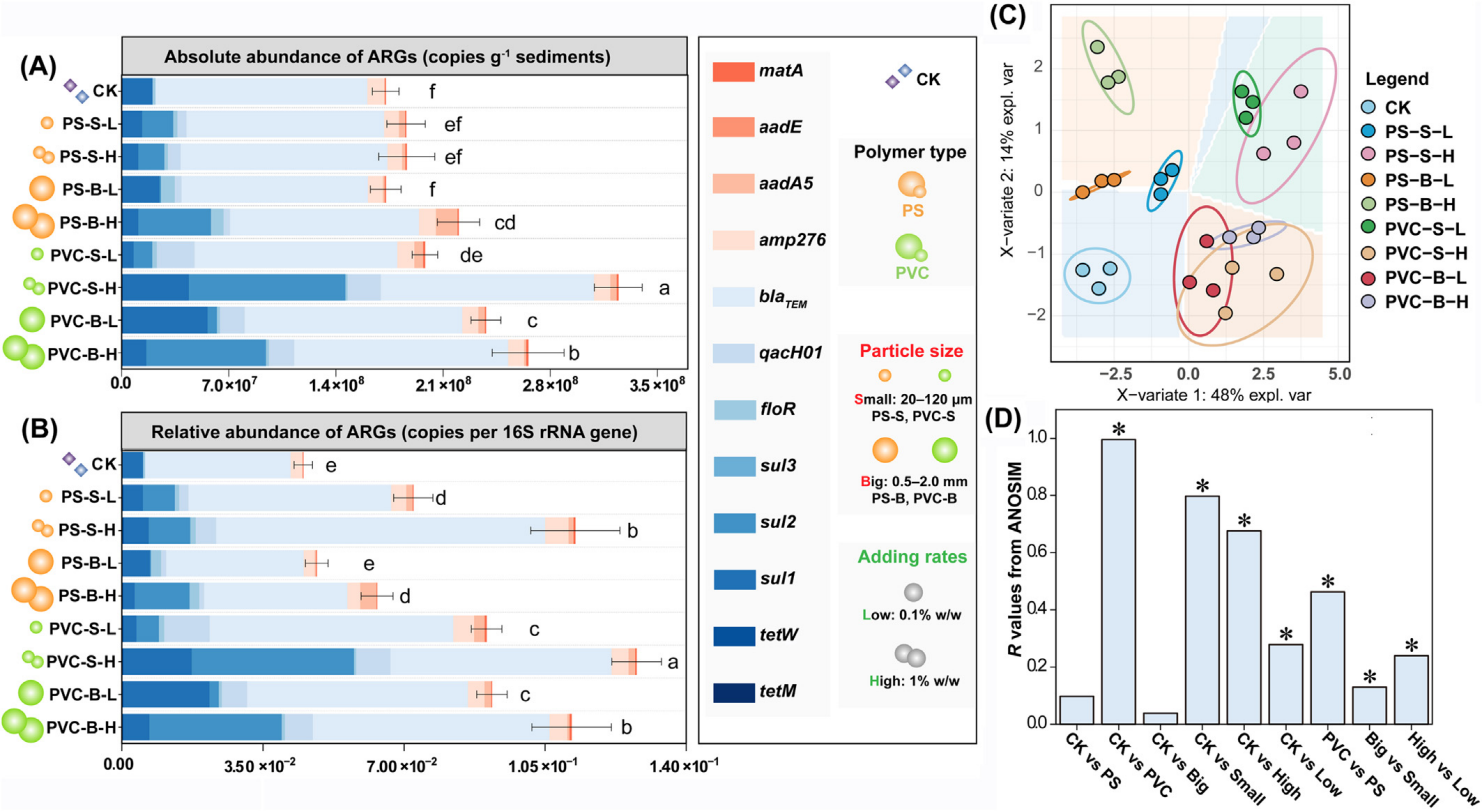
Effects of MPs on (A) the absolute abundance and (B) relative abundance of targeted ARGs normalized by the 16S rRNA gene copy number in the marine sediments. The different small letters represent significant difference in the total ARG abundance among the different treatments (Duncan's multiple-comparison test, *n* = 3, *P* < 0.05). (C) Partial least squares discriminant analysis (PLS-DA) of sediment ARGs profiles. (D) The *R* values of analyses on the similarities (ANOSIM) represent the inter-group and within-group differences and similarities in the ARG profiles. The *R* values close to 1 indicate the large variation between the groups, while the *R* values close to or less than 0 represent the non-significant difference within inter-group. The asterisk (∗) indicates that the test is statistically significant with high reliability (*P* < 0.05).

As expected, after 60 days of MP cultivation, the total absolute abundance and relative abundance of 12 targeted ARGs in all the MP treatments were (2.20–3.25) × 10^8^ copies/g and (4.84–12.8) × 10^−2^ copies per 16S rRNA gene, respectively, increasing by 14.9%–88.1% and 7.46%–188% compared to the control (Fig. 2A and B). Notably, the absolute abundances of *flo*R and *aad*E, defined as high-risk ARGs due to their extremely high prevalence, gene mobility, and potential pathogenicity [37], increased by 57.2%–396% and 12.1%–49.9% in the MP treatments relative to the control, respectively. Among them, *floR*, identified in clinically significant pathogenic bacteria (*Salmonella*) [38], has been detected in various seafood products, including grouper, shrimp, and *Mylopharyngdon piceus* [39,40], indicating that high-risk ARGs can be transferred to humans through the food chain, causing harm or untreatable diseases. These results evidenced our hypothesis that MP pollution elevated ARG abundance and risk levels in the mariculture environments.

Polymer types and MP particle sizes had varied effects on ARG profiles in the sediments (Fig. 2). Regarding the polymer types, the total ARG absolute abundance was significantly increased by 1.15–1.88-fold in the PVC MP treatment, whereas in the PS treatment, the increase was only 1.00–1.27-fold (Fig. 2A). The promotion effect was also consistently supported by the results of the normalized ARG copy numbers per 16S rRNA (Fig. 2B). For the polymer types, PVC MPs exhibited a stronger enrichment effect on the relative abundance of ARGs than PS MPs. Notably, for the high-risk genes such as *flo*R, *aad*E, and *aad*A5, their absolute abundances significantly increased by 1.03–1.57-fold, 1.12–1.26-fold, and 52.4–225-fold, respectively, and the relative abundances significantly increased by 1.05–2.75-fold, 1.75–1.96-fold, and 84.3–395-fold. For *flo*R and *aad*A5, PS MPs significantly increased their absolute abundances by 1.23–4.96-fold and 81.0–558-fold, and their relative abundances by 2.22–5.33-fold and 47.1–616-fold, respectively (Fig. S5), further confirming that PVC MPs caused a higher risk of AMR prevalence than PS MPs.

Regarding particle sizes, both PS-S and PS-B exhibited similar promotion effects on the absolute abundance of ARGs. However, the relative abundance of ARGs in the PS-S was significantly higher than in the PS-B (Fig. 2B). This difference might be attributed to the increased surface area of smaller particles, like PS-S, which could have provided more attachment sites for bacteria. Zhu et al. [41] revealed that plastispheres act as hotspots for ARGs, with their surfaces tending to accumulate ARG hosts. The enhanced relative abundance of ARGs in PS-S suggested that smaller particles might more effectively concentrate these bacteria, leading to greater ARG enrichment. Moreover, there was evidence suggesting that smaller-sized MPs were more likely to be ingested by benthic invertebrates (e.g., mussels and clams), and the ARGs they carried might more easily accumulate in the tissues of these organisms, posing a potential threat to seafood safety and the food chain [42,43], which needs to be further verified in the future.

The antibiotic resistome determines the antibiotic treatment strategies in mariculture, husbandry, and clinical settings [1]. Understanding the pollutant effects on resistome dynamics could contribute to a more comprehensive environmental risk assessment of ARGs [37]. The PLS-DA results showed that ARG composition in all the treatments was distinct from that of the control (ANOSIM *R* = 0.984, *P* = 0.001) (Fig. 2C and D), suggesting that MPs drove the succession of antibiotic resistome in the sediments. Specifically, the greatest variation in the ARG profiles was found between the type of PS and PVC (*R* = 0.462), followed by dose (*R* = 0.239) and size of MPs (*R* = 0.130) (Fig. 2D). Thus, the primary factor determining the sediment resistome evolution was polymer type rather than MP size. Moreover, the primary types of ARGs enriched by MPs in the sediments included those associated with antibiotic inactivation (*aad*E, *aad*A5, *amp*276, and *bla*_TEM_), efflux pumps (*mat*A, *flo*R, and *qac*H01), and cell protection (*sul*2, *tet*M and *tet*W). The abundances of these genes were significantly positively correlated with the contents of heavy metals (Cr and Pb) in the sediments (Fig. S6), suggesting that certain exogenous heavy metals exerted selective pressure on microbial communities, thereby promoting an increase in ARG abundance. Previous studies have documented co-resistance between heavy metals and antibiotics, providing evidence that resistance to both can co-occur within microbial communities. For instance, *mat*A and *flo*R have demonstrated potential roles in efflux mechanisms against Cu and Hg in contaminated environments [44], while *tet*A and *tet*M encode ribosome protection proteins that stabilize ribosomal structure, helping bacteria maintain polypeptide synthesis under heavy metal stress from Cd, Zn, Cu, and Pb [45]. Such protection mechanisms have conferred resistance to specific bacterial taxa, including *Gammaproteobacteria*, *Bacteroidia*, and *Actinomycetia*, enabling them to survive and proliferate under metal-contaminated conditions [46,47]. These results suggested that the heavy metals (especially Cr and Pb) leached from MPs (details are shown in Text S3 and Table S4) would indirectly contribute to ARG enrichment in sediments. Furthermore, some studies reported that stress from contaminants such as diesel oil, antibiotics, and metallic nanoparticles may enrich ARB with these ARGs to combat external pressures [23,48]. Additionally, PS MPs selectively enriched TC resistance genes *tet*W, while PVC MPs increased the absolute abundance of SMX resistance genes (*sul*1, *sul* 2, and *sul* 3) (Fig. 2A). These findings are consistent with a field survey by Sun et al. [18] in mangrove environments, where the abundance of TC resistance genes on PS surfaces was significantly higher than on other MPs. This result was rationalized by the selective adsorption of PS and PVC MPs on antibiotics. The adsorption of TC by PS MPs was driven by π-π interactions between the aromatic rings in both PS and TC, thereby strengthening their affinity for each other [49]. In contrast, SMX showed a preference for PVC due to electrostatic interactions between the polar functional groups of SMX and the polar sites created by chlorine atoms on the PVC surface, as well as possible surface complexation involving hydrogen bonding or coordination bonds between the sulfonamide group of SMX and hydroxyl groups on PVC [50]. Additionally, this adsorption will enrich the antibiotic on the MP surface, thereby increasing the selective pressure on bacteria colonizing their surface [13]. This implied that residual antibiotics may interact with MPs, resulting in a combined exposure effect on ARGs. This underscored the importance of understanding the synergistic effects of MPs with environmental factors such as antibiotics and heavy metals on the spread of ARGs. Assessing these interactions was crucial for evaluating the comprehensive ecological risks associated with MPs and for developing effective long-term mitigation strategies to protect environmental health.

Additionally, *qac*H01 and *flo*R were significantly enriched in PS MP treatments, while *mat*A, *bla*_TEM_, *amp*276, *aad*A5, and *aad*E were significantly enriched in the PVC MP treatments (Fig. S6), consistent with the enrichment patterns in estuarine plastisphere [51]. This differential enrichment likely reflected the varying interactions between different types of MPs and ARGs hosts, influenced by the specific properties of each polymer type. Similarly, under contaminants like MP stress [48], community composition [20] drove HGT, and adaptive evolution [26] could enrich the aforementioned ARGs in marine sediments. The observed ARG enrichment patterns underscored the need for further investigation into how different types of MPs and their interactions with environmental factors contribute to the evolution and spread of antibiotic resistance. Understanding these dynamics is crucial for developing targeted strategies to mitigate the impact of MPs on marine ecosystems and public health, which will be discussed in the following section.

### 3.2 MPs-driven deterministic microbial community assembly and host bacterial enrichment

**Fig. 3:**
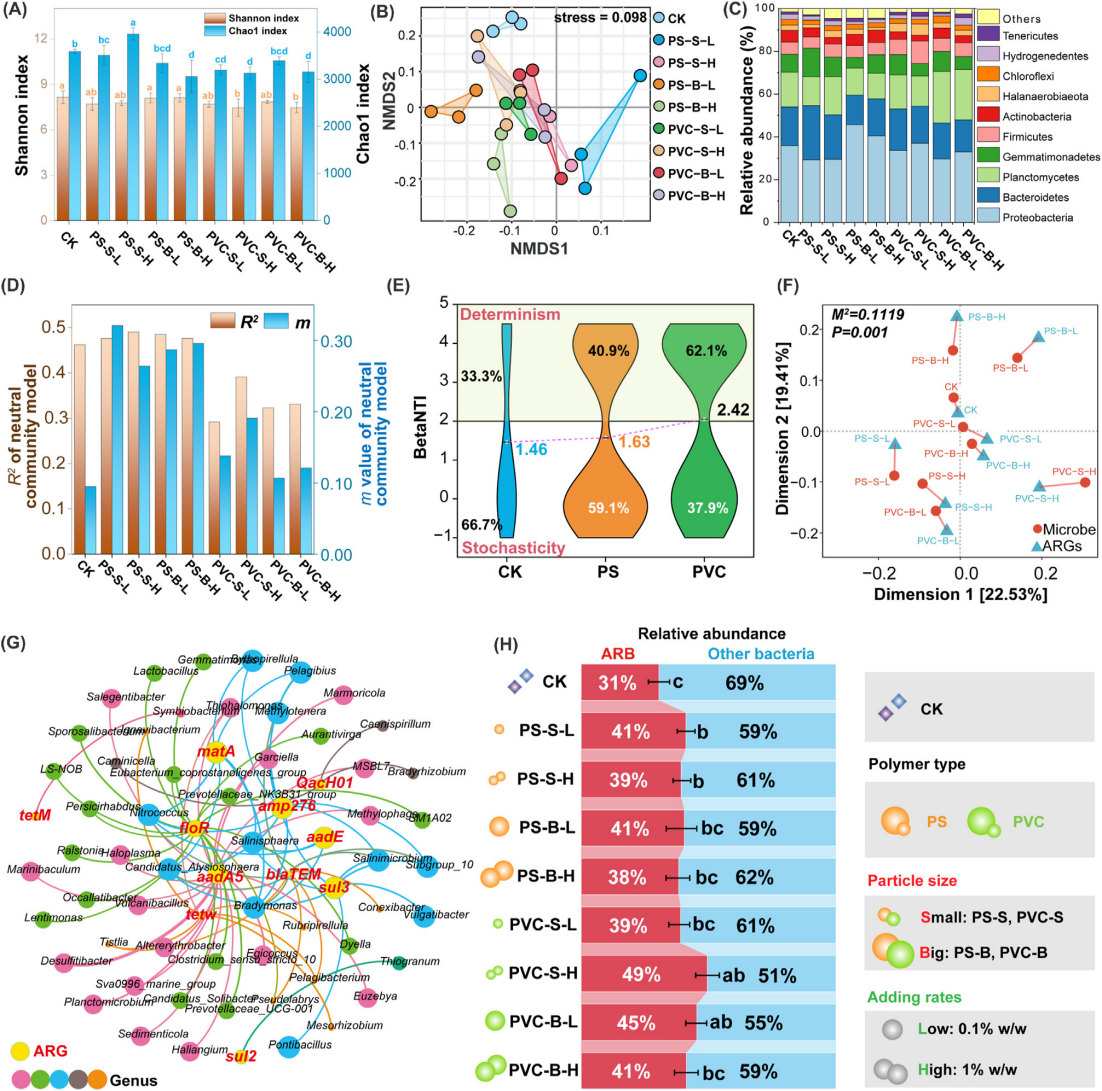
Effect of MPs on microbial community structure in the marine sediments. (A) Shannon and Chao1 index showing significant differences in the evenness and richness of microbiota. (B) NMDS plots based on Bray-Curtis distance on the bacterial phylum level. (C) The relative abundance of bacteria taxon at phylum level. (D) Sloan neutral community model statistics (*m*: species migration rate; *R*^*2*^: model goodness of fit) for the bacterial community of different treatments. (E) Beta nearest taxon index (betaNTI) values and relative contribution (%) of deterministic and stochastic processes in the assembly of sediment bacterial communities. (F) Procrustes correlation analysis between bacterial compositions (16S rRNA gene OTU data) and ARG profiles based on Bray-Curtis dissimilarity metrics (*M*^*2*^ = 0.1119, *P* = 0.001). (G) Co-occurrence network analysis of ARGs and dominant microbial genera [with abundance > 0.1, Spearman's correlation (*R* > 0.7, *P* < 0.01)]. (H) The relative abundance of potential ARB. The different small letters reflect significant difference among the different treatments (Duncan's multiple-comparison test, *n* = 3, *P* < 0.05).

To determine the roles of bacterial communities in ARG evolution in the sediments, the changes in microbial communities in sediments under MP pollution were explored (Fig. 3). Compared to the control, the Shannon and Chao1 indices were not affected by PS MPs, but were significantly decreased by PVC MPs (Fig. 3A). This effect might be attributed to the PVC MPs, which, due to their strong negative charge and the release of exogenous additives such as plasticizers and stabilizers (Tables S3–S5), likely caused more severe disruption to the sediment environment compared to PS MPs. The negative charge of PVC MPs could induce electrostatic repulsion with negatively charged bacterial cells, which might inhibit bacterial attachment and colonization. Additionally, the release of chemical additives from PVC MPs might have introduced toxic substances or altered the sediment chemistry in ways that are detrimental to bacterial diversity and richness. This disruption could lead to a reduction in the overall microbial community diversity and richness. Conversely, PS MPs, which exhibited strong hydrophobicity and a lower negative charge (Table S3), did not significantly alter the Shannon and Chao1 indices. The hydrophobicity of PS MPs could facilitate bacterial attachment and biofilm formation, supporting a more stable microbial community. The lack of significant impact on community diversity and richness suggested that while PS MPs influenced microbial colonization, they might not have as severe an effect on the overall community structure as PVC MPs. These findings were consistent with recent studies indicating that the physicochemical properties of MPs, including their surface charge and the additives, played a crucial role in shaping microbial community dynamics [52,53]. Setiyawan et al. [53] showed that MP surface properties could affect bacterial colonization and community composition differently, depending on the type of polymer and the environmental conditions. Our results aligned with these studies, reinforcing the idea that MPs could have varied impacts on microbial communities based on their chemical and physical characteristics. Overall, the observed effects underscored the importance of considering MP types and their physicochemical properties when evaluating their impact on sediment microbial communities and ARG evolution. Understanding these interactions is essential for assessing the broader ecological and environmental implications of MP pollution.

In addition, the OTU-based NMDS and ANOSIM analysis showed that all the MP treatments significantly changed the composition of bacterial communities, and the polymer type-dependent effects (*R* = 0.624, *P* = 0.003) were stronger than size-dependent effects (*R* = 0.353) (Fig. 3B, Table S11). Although studies have demonstrated that MPs exhibit size-dependent effects on bacteria, influencing growth, biofilm formation, and community structure [26,54], our findings highlighted a more pronounced sensitivity of microbial communities to polymer type. While smaller MPs (less than 1 μm) generally have a greater impact on bacterial viability and metabolic activity compared to larger particles [55], the chemical composition of MPs appeared to play a more significant role in shaping bacterial communities. For example, smaller particles may enhance the uptake of harmful substances by bacteria and promote biofilm formation due to their larger surface area [13,56], but these effects can be overshadowed by the influence of polymer type on microbial competition and metabolic pathways. Han et al. [54] reported that specific polymer types led to varied responses in bacterial growth and diversity, which often overshadowed the effects attributed to particle size. Similarly, Frère et al. [57] emphasized that chemical properties associated with different polymers can more significantly alter microbial metabolic pathways than size variations. These observations aligned with the ARG patterns observed in the sediments, further suggesting that polymer type played a dominant role in driving ARG evolution by shaping microbial community structure in sediments.

The alterations of the bacterial abundance from the top 10 phyla showed that the four MPs selectively enriched different bacteria (Fig. 3C). All the MP treatments enriched biofilm-forming bacterial taxa such as Firmicutes, Gemmatimonadetes, and Tenericutes. Notably, Proteobacteria, Bacteroidetes, Gemmatimonadetes, and Firmicutes, widely acknowledged as host bacteria for ARGs [41], significantly accumulated in the PS MP treatments (Fig. 3C). These bacteria are widely detected in the plastisphere due to their ability to produce flagella or pili and secrete extracellular polymer to form biofilms [58,59]. Consistently, the BugBase phenotype prediction confirmed that PS exposure enhanced biofilm formation in the sediment communities (Fig. S7A). Moreover, Planctomycetes, Halanaerobiaeota, and Chloroflexi, which could contribute to the bacterial resistance to contaminant stress [60], consistent with the observed changes in the bioavailable heavy metal contents in the sediments due to MP addition in this study (Fig. S8). These results suggested that the enrichment of ARGs in the PVC-contaminated sediments, driven by the pollution stress, triggers bacterial community succession.

The changes in microbial communities depended on community assembly processes, which are controlled by the deterministic processes based on niche theory and the stochastic processes based on neutral theory [36]. The neutral and null models were established (Fig. 3D and E). The neutral community model results showed that PS MPs (47.6%–49.0%) had little effect on the neutral process of community assembly, while PVC MPs (29.2%–39.1%) decreased the neutral process of bacterial community assembly when compared with control (46.2%). Notably, the migration rates (*m*) in PS MP treatments (0.26–0.32) were higher than those in PVC MP treatments (0.11–0.19) (Table S12), suggesting that the bacteria in PS MPs-contaminated sediments have greater dissemination ability, which may exacerbate sediment ARG migration. Furthermore, the null model analysis indicated that PS MPs and PVC MPs increased the deterministic assembly processes of the communities from 33.3% to 40.9% and 62.1%, respectively, suggesting that PVC MPs shifted the assembly from stochasticity process to deterministic process (Fig. 3E). As selective pressure increases, deterministic assembly typically becomes more dominant, while stochastic assembly decreases, indicating that PVC MPs exert greater selective pressure on the sedimentary microorganisms [61]. These might be related to the observed selective pressure on bacteria by MPs [62,63], especially Zn and Cu released from PVC, as well as phthalates and BPA (Tables S4 and S5). Deterministic processes were found to play a significant role in shaping the bacterial communities and antibiotic resistome of the plastisphere in various environments [17,64]. These processes were observed to dominate the bacterial community assembly in plastispheres, leading to distinct microbial compositions and functions, including the enrichment of potential pathogens and ARBs. The deterministic community assembly process induced by enhanced selective pressure would inevitably lead to changes in ARG host abundance [17]. Procrustes analysis further confirmed a significant correlation between ARG composition and microbial community composition (*M*^2^ = 0.112, *P* = 0.001) (Fig. 3F), confirming a close host-supplier relationship between ARGs and potential bacterial hosts in the sediment. These results further demonstrated that MPs could increase the abundance of ARGs by increasing the deterministic assembly processes of the bacterial communities in the sediments. The deterministic nature of these processes underscored the importance of understanding the ecological drivers controlling the sediment microbial communities, especially in the context of antibiotic resistance and pathogen recruitment, emphasizing the need for further research and conservation efforts in the face of increasing plastic pollution.

Based on the significant correlations between ARGs and microbial communities, a co-occurrence network was constructed at the genus level to elucidate the symbiotic patterns between bacterial general hosts and ARGs (Fig. 3G, Table S13). The results implied that MPs promoted the enrichment of multidrug-resistant bacteria in the sediments. Specifically, PS enriched *Ralstonia*, *Planctomicrobium*, *Vulcanibacillus*, *Desulfitibacter*, and *Conexibacter* carrying *flo*R, *aad*A5, and *bla*_TEM_, while PVC enriched *Tistlia*, *Subgroup_10*, *Candidatus_Alysiosphaera*, and *Pelagibius* carrying *tet*W, *sul*3, *mat*A, and *bla*_TEM_. The network analysis revealed that all the MP treatments (38.3%–49.5%) accumulated more potential host bacteria in the sediments than the control (30.6%), and PVC MPs (39.0%–49.5%) showed more facilitation effect than PS MPs (38.3%–41.1%) (Fig. 3H). These results aligned with the observed ARGs abundance (Fig. 2), confirming that MPs could promote the dissemination of ARGs in mariculture sediment by inducing the enrichment of potential host bacteria with a polymer type-dependent effect. Additionally, MPs increased the abundance of *Ralstonia*, *Conexibacter*, and *Marinibaculum* (Fig. S9), which were identified as potential ARG hosts in this study (Fig. 3G), and are also pathogens for aquatic organisms and humans [41,65]. These results further confirmed that MPs could enhance the AMR risks by enriching these resistant pathogens. Notably, *bla*_TEM_ was frequently co-occurring in the same bacteria as ARGs such as *mat*A and *amp*276, suggesting that these ARGs might be on the same MGEs and transmitted between sediment microorganisms via HGT [19,66].

Plastisphere is a hotspot for the enrichment of host bacteria and the occurrence of HGT [41], which might lead to the observed co-occurrence patterns (Fig. 3G). Hence, the process of bacterial growth and interactions on the surfaces of MPs is crucial for understanding the key mechanisms by which MPs enhance ARG pollution, which will be discussed in the following section.

### 3.3 Biofilm enriched host bacteria and served as a hotspot for horizontal gene transfer of antibiotic resistance plasmid

**Fig. 4:**
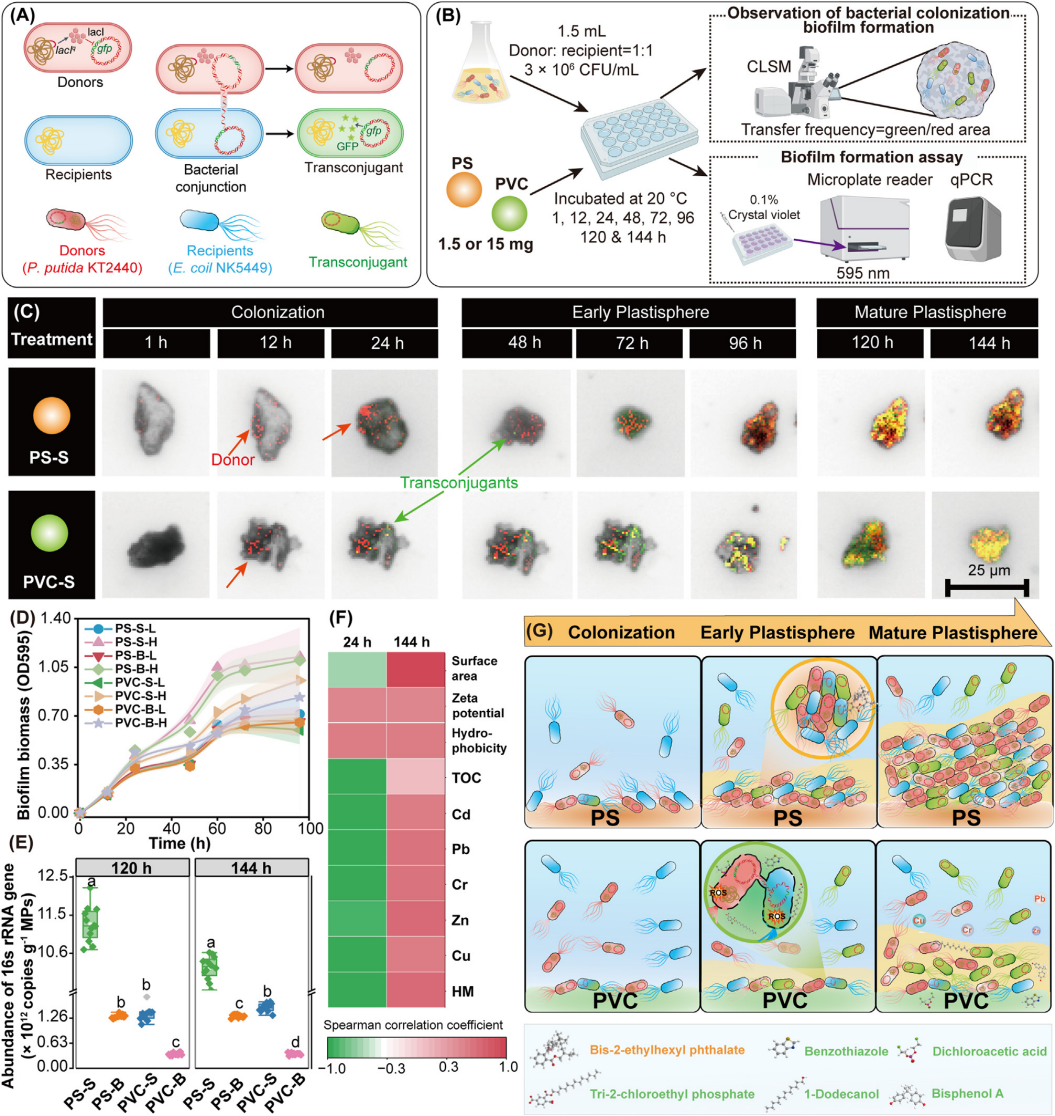
(A) Schematic diagram of fluorescence expression of bacterial conjugative transfer. (B) Flow chart of biofilm formation on MPs experiments. (C) Images of donors (red fluorescence) and transconjugants (green fluorescence) in the biofilms captured by an LSCM, and (D) biofilm biomass of the mixed donor and recipient on the MPs, the transparent area width represented the error range. (E) The biomass of the biofilm on mature plastisphere. (F) Spearman's correlation between biomass and physicochemical properties of MPs during biofilm colonization and maturation periods. (G) Schematic diagram showing the donor (red) colonization process and transconjugant (green) formation on the surface of small size MPs.

The strain *P. putida* KT2440::*lacI*^*q*^-*dsRed* as donor and *E. coli* NK5449 as recipient was used to investigate the biofilm formation and the ARG spreading process in the biofilm on the two small MPs (Fig. 4A and B). The plastisphere formation process can be categorized into bacterial colonization, plastisphere growth, and maturation phase (Fig. 4C). Differences in the plastisphere formation on PS and PVC MPs were evident, as shown by the LSCM images (see more details in the caption of Fig. S10A, B). At the bacterial colonization stage (1–24 h), PS MPs exhibited higher adherence of bacteria cells on their surfaces compared to PVC MPs (Figs. 4C, S10A). During the early plastisphere growth stage (48–96 h), the colonization rate on PS MPs reached 42.8% ± 31.0%, which was higher than that of PVC MPs (39.5% ± 20.4%). At the plastisphere maturation stage (120–144 h), PS-S and PVC-S were completely covered by bacteria, and the biomass on MPs had stabilized, as confirmed by crystal violet staining and real-time qPCR (Fig. 4D and E). Furthermore, the biomass on PS MPs [(1.32–11.1) × 10^12^ copies/g MPs)] was significantly higher than that on PVC MPs [(0.36–1.53) × 10^12^ copies/g MPs)]. Moreover, small MPs [(1.42–11.1) × 10^12^ copies/g MPs)] exhibited significantly higher biomass than big MPs [(0.36–1.47) × 10^12^ copies/g MPs)].

The hydrophobic surface of MPs has been proven to have a higher affinity for hydrophobic bacteria and their produced adhesins (e.g., extracellular polysaccharides, proteins, and DNA) [25,67], making bacteria more inclined to colonize the hydrophobic surface of PS MPs. Meanwhile, the heightened negative charge of PVC MPs could induce electrostatic repulsion between the MP surface and negatively charged bacteria, impeding bacterial colonization. This was supported by Spearman correlation analysis (Fig. 4F), which revealed that the biofilm biomass on four MPs during the initial plastisphere development (24 h) positively correlated with hydrophobicity and zeta potential of the MPs. Additionally, the significant negative correlation between total organic carbon (TOC) and heavy metals (Cd, Pb, and Cu) content with the biofilm biomass (Fig. 4F) further implied that the released exogenous additives of MPs inhibited the growth of bacteria and flagella [31,68], consequently diminishing their colonization ability and resulting in reduced biomass on PVC MPs. While previous studies indicated that rougher MP surfaces are more conducive to bacterial pili growth, attachment, and colonization [69], our findings contrast this by showing that the PVC MPs, despite having more gaps and cracks on their surfaces (Fig. S1A), supported lower bacterial colonization. This suggested that other factors, such as the chemical properties of PVC MPs and the release of inhibitory exogenous additives, may outweigh the physical advantages typically associated with rougher surfaces. During the maturation phase (144 h), the particle sizes were negatively correlated with biomass, indicating that small MPs with larger specific surface areas provided more growth sites for biofilm development [67]. In summary, compared to PVC MPs with a surface characterized by strong negative potential and rich exogenous additives, the strong hydrophobicity and weaker zeta potential of PS MPs facilitated the dense biofilm formation (Fig. 4G). This observation was consistent with the predicted phenotypic patterns of biofilm-forming bacteria in the sediment communities (Fig. S7A), confirming that PS MPs could alter the structure and function of sediment bacterial communities by recruiting bacteria for biofilm formation. Additionally, Zhao et al. [26] and Zhu et al. [64] discussed the role of MPs as reservoirs and vectors for ARGs in constructed wetlands and soil, highlighting the importance of MP characteristics in ARGs dissemination. These studies reinforced our findings and highlighted how the interplay between MP size, surface characteristics, and bacterial colonization dynamics underscored the complex ecological impacts of MPs across various environments.

Compared to free bacteria or those in natural aggregates, the elevated bacterial density in the plastisphere promoted cellular contact, creating a favorable environment for ARG exchange [60]. To further determine the occurrence frequency of HGT in the plastisphere, the transconjugant number and the conjugation frequency in the biofilm were analyzed (Fig. S10B, C). The appearance of green fluorescence representing transconjugants on the surface of PVC MPs (24 h) occurred earlier than PS MPs (48 h) (Figs. 4C, S10B), indicating an earlier HGT event on PVC MPs. During the plastisphere growth and maturation stage (72–144 h), the ratio of transconjugants to donors on PVC MPs (0.53–1.12) was significantly higher than those of PS MPs (0.22–0.81) (Fig. S10C), demonstrating the higher gene mobility in the biofilm on PVC MPs. This is consistent with the Bugbase phenotypic prediction of sediment communities (Fig. S7A). Additionally, the MP biofilms were confirmed to facilitate natural transformation, which enhanced the transmission of ARGs [67]. Future studies should aim to clarify the HGT processes occurring in biofilms to deepen understanding of the complex transmission dynamics of AMR in the environment stressed by MPs. Notably, vertical gene transfer (VGT) may play a substantial role in the propagation of ARGs within MP-associated biofilm. The higher rate of bacterial proliferation on the surfaces of PS MPs compared to PVC MPs (Fig. 4D) implied that ARGs would be propagated more quickly via VGT on PS MPs. However, a greater number of transconjugants were found on the surface of PVC MPs, indicating that HGT facilitated by MPs had a more significant impact on the transmission of ARGs than VGT. Although previous studies have also found that HGT contributed the most to shape the ARG transmission in sediment environments [70], the contributions of VGT and HGT to the variations in ARG abundance across different MPs remain unclear, which warrants further investigation in the future. Moreover, a higher frequency of HGT should be found in the biofilm of PS MPs due to its higher biomass than PVC MPs. Paradoxically, the biofilm of PS MPs in this study had a higher biomass, in which the frequency of HGT was lower than that of PVC MPs. It was reported that MPs and the released additives may play crucial roles in promoting HGT [26,60]. Therefore, this study hypothesizes that the complex additives released by PVC MPs would significantly contribute to the higher frequency of HGT on their surface. This will be further discussed in the following section.

### 3.4 Microplastic particles and leachates synergistically enhanced horizontal ARG transfer

**Fig. 5:**
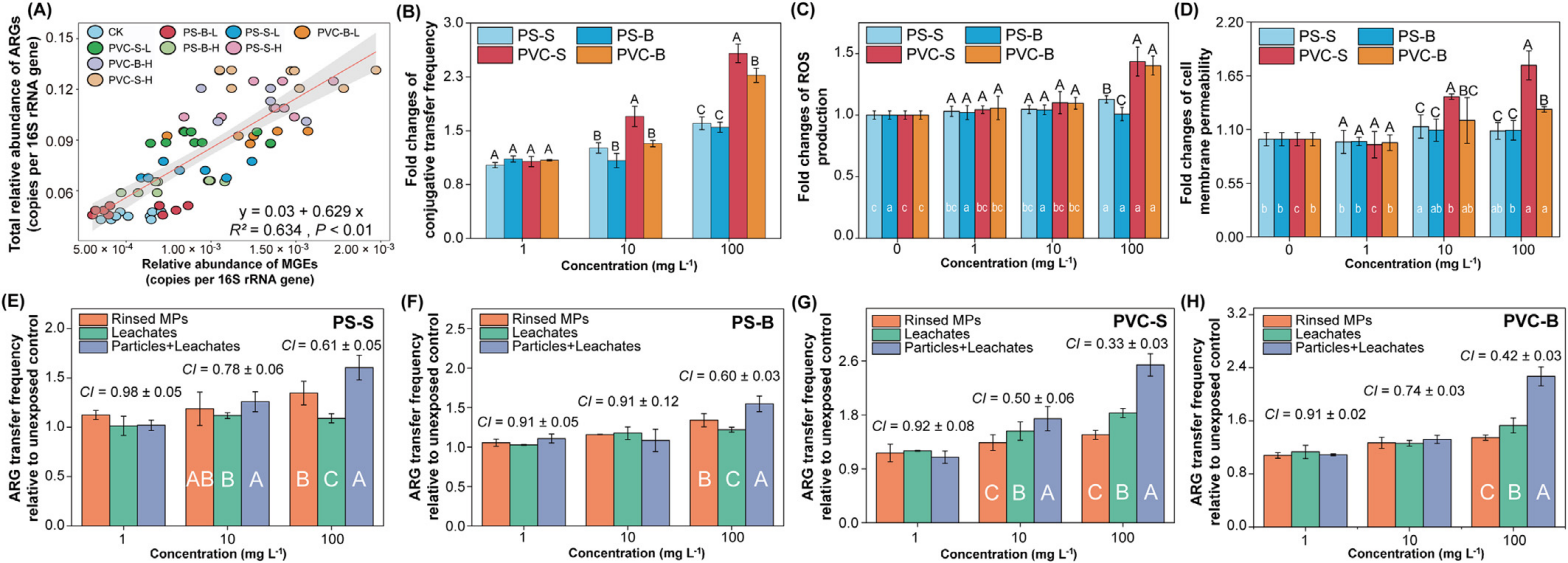
(A) The linear regression between the abundances of ARGs and MGEs in the marine sediments polluted by MPs (shaded area denotes 95% confidence interval). (B) Effect of MPs on the conjugative transfer frequency of plasmid RP4 between the donor *P. putida* KT2440 and recipient *E. coli* NK5449. Fold changes in (C) intracellular ROS production of the recipient and (D) cell membrane permeability. Effects of the rinsed MPs (MPs particles), leachates, and non-rinsed MPs (total contribution) of (E) PS-S, (F) PS-B, (G) PVC-S and (H) PVC-B on the conjugative transfer frequency. Based on the Bliss independence model, the combined effect of MPs particles and leachate was considered synergistic when *CI* < 1 or antagonistic when *CI* > 1 The different small letters represent a significant difference among the different MPs treatments, the capital letters indicate a significant difference among the treatments with the same concentrations (Duncan's multiple-comparison test, *n* = 3, *P* < 0.05).

To verify whether the promoted gene exchange in the plastisphere enhanced the HGT potential in sediments, the abundances of two typical MGEs, integrase genes (*intI*1) and transposons (*tnp*A), was determined in the sedimentary communities. During the sediment aging process, the total absolute abundance of MGEs significantly increased from (2.11 ± 0.33) × 10^7^ copies/g to (4.66 ± 0.67) × 10^7^ copies/g, equivalent to their abundances in mariculture areas [(0.28–250) × 10^7^ copies/g] (Table S8). Additionally, the total abundance of MGEs and ARGs was significantly positively correlated (Fig. S5B, C), confirming that antibiotic pollution in mariculture could exacerbate ARG pollution by increasing HGT potential. Following a 60-day exposure to MPs, the total relative abundance of MGEs in the sediments significantly increased by 27.9%–161% compared to the control (Fig. S11). Moreover, the relative abundance of MGEs and ARGs was significantly positively correlated (Fig. 5A), further confirming that HGT is a crucial pathway through which MPs boosted ARG levels and risks in the sediments.

Conjugative transfer is the primary mechanism for the spread of ARGs in the environment, and its contribution to ARG dissemination far exceeds that of transformation and transduction [21,23]. Therefore, the impact of MPs on plasmid-mediated ARG conjugative transfer was investigated (Fig. 4A). Compared to the control, the transconjugant number and conjugative transfer frequency increased by 8.45%–104% and 2.03%–60.4%, respectively, with increasing concentrations of the two MPs (Figs. 5B, S11A), these findings were consistent with the observed ARG abundance patterns in sediments following MPs exposure (Fig. 2A). Notably, in the PVC MP treatments, the transconjugant number exhibited a trend of initially increasing and then decreasing with increasing exposure concentration, whereas the transfer frequency showed a dose-dependent pattern (*R* = 0.92, *P* < 0.05). This discrepancy was primarily associated with the significant reduction (64.8%–67.4%) in recipient bacteria exposed to the high concentration of PVC (100 mg/L) (Fig. S11B). This further confirmed that PVC MP contamination stress could inhibit bacterial growth. Moreover, the promoting effects of PVC MPs on the conjugative transfer were stronger than those of PS MPs, and small MPs were more potent than big MPs (Fig. 5B). Zhao et al. [26] reported similar findings, showing that MPs promoted ARG transfer in a size-dependent manner, with 100 μm particles exhibiting the most substantial promotion compared to 4 mm particles. These studies demonstrated that MP exposure promoted ARG transfer between bacteria, with the extent of promotion depending on polymer type and particle size. Smaller MPs provided more sites for bacterial colonization (Fig. 4D and E), which facilitated gene exchange, while the release of more exogenous additives from PVC MPs affected bacterial metabolic activities.

The ROS-inducing potential of MPs played a key role in their cytotoxic effects across various cell types [71]. Bacteria under pollutant stress overproduced ROS, which oxidized DNA and phospholipids, leading to the disruption of double-stranded DNA and cell membrane structures. This process promoted error-prone polymerases that increased genetic diversity, thereby heightening the likelihood of integrating ARGs into the bacterial genome [24,31]. Therefore, the changes in ROS production and membrane permeability in recipient bacteria were determined (Fig. 5C and D). The results demonstrated that PS MPs had little effect on the intracellular ROS levels compared to the control. In contrast, the intracellular ROS levels in the bacteria exposed to PVC MPs exhibited a dose-dependent effect, consistent with the enhanced conjugation frequency pattern (Fig. 5B). Tax4Fun functional predictions further revealed that PVC MPs significantly enriched the pathways related to DNA replication and repair, as well as cell growth and death in sediment bacterial communities (Fig. S7B). Furthermore, the flow cytometry analysis confirmed that the increased cell membrane permeability of recipient bacteria under MPs exposure was consistent with the ROS levels (Fig. S12C), with PVC MPs exhibiting a stronger effect than PS MPs and small MPs having a greater impact than big MPs (Fig. 5D). These results confirmed that MPs could promote the HGT by inducing oxidative stress and enhancing membrane permeability. In addition, oxidative stress induced by MPs in sediments could trigger bacterial mutations via introducing errors during DNA replication or repair [72]. Accordingly, homologous recombination during DNA repair and reduced membrane permeability could facilitate the transformation of extracellular ARGs [67,73]. Zeng et al. [17] attributed the oxidative stress caused by PVC MPs to chlorine radicals generated from C–Cl bond cleavage. However, these radicals would not generate under our experimental conditions, suggesting that chemical substances leaching from MPs were the primary cause of ROS production. This hypothesis was supported by a strong positive correlation between conjugation frequency and the levels of heavy metals (Cu, Cr, Pb) and TOC in MP leachate (Fig. S12C). These results implied that exogenous additives, especially in small PVC MPs, were likely responsible for the strong potential to induce ARG conjugative transfer. Therefore, the contributions of rinsed MPs (without exogenous additives) and MP leachates (filtering out MPs) to the dissemination of ARGs via HGT were further investigated.

For PS MPs, the rinsed PS MPs without exogenous additives (10–100 mg/L) increased the conjugation transfer frequency by 15.8%–34.7%, significantly stronger than their leachate (9.03%–21.8%) (Fig. 5E–H). This suggested that the promoted HGT by PS MPs is dominated by the particle effect, as PS MPs were more conducive to the formation of a dense biofilm (Fig. 4G), thereby enhancing the exchange of genes between bacteria. In addition, for PVC, the promotion effect of PVC MPs leachates on the conjugation frequency (13.2%–79.1%) was significantly higher than those of the particles (8.00%–43.6%), implying that the enhanced HGT by PVC MPs mainly contributed by exogenous additives. The substances in the leachates of PVC MPs, including BPA, tridecyl ester, and heavy metals (Tables S4, S5), have been confirmed to directly damage the bacterial cell membranes and regulate the expression of conjugation or quorum-sensing-related genes at environmentally relevant concentrations, thereby promoting HGT [22,74,75]. In this study, based on Tax4Fun functional prediction, it was also confirmed that PVC MPs promoted the function of signal transduction in the sediment microbial community, especially the enrichment effect of PVC-S was the most significant (Fig. S7B). These results evidenced that the leachate of PVC MPs not only caused oxidative stress to bacteria but also promoted the dissemination of ARGs in the sediment by regulating quorum-sensing signal transduction.

Based on the Bliss independence model analysis, the promotion of conjugation transfer by four types of MP particles and leachate all showed a synergistic effect (*CI* < 1, Fig. 5E–H). Notably, the lowest *CI* of PVC-S among all the MP treatments indicated that the small PVC MPs, capable of releasing more exogenous additives (Tables S4, S5), exhibited the strongest synergistic effect with their leachates on the conjugation transfer. This synergistic effect might be attributed to the fact that exogenous additives released by MPs are trapped in the extracellular polymers released by colonizing bacteria, increasing the time and opportunities for bacteria to be exposed to exogenous additives. These results highlighted that the impact of exogenous additives from MPs on the risk of ARG dissemination has been underestimated in the past, and future research should not overlook the synergistic effects of MPs particles and leachate to better assess their environmental risks.

## 4 Conclusions

**Fig. 6:**
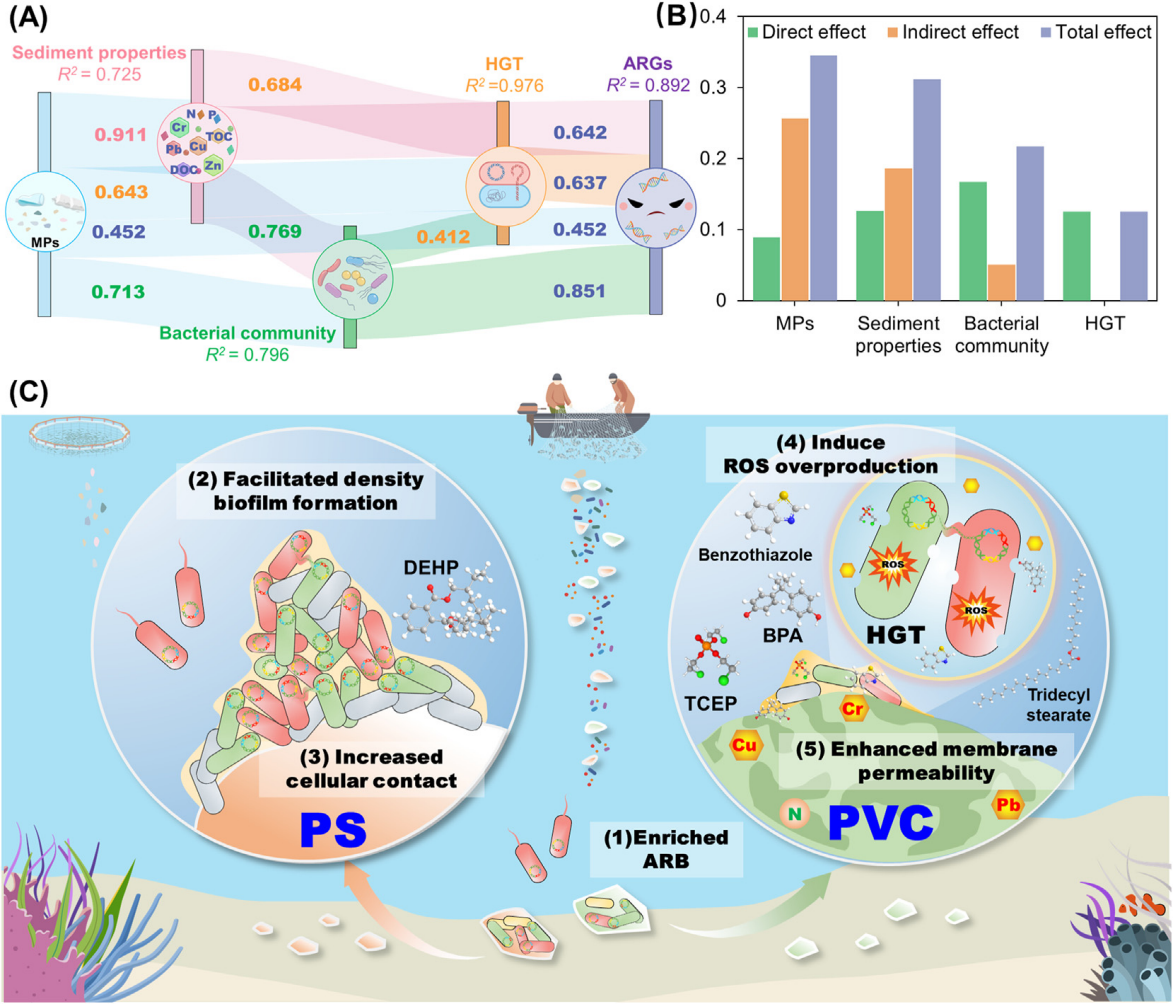
(A) Relationships among MPs, sediments physicochemical parameters, bacterial communities, HGT, and ARGs based on the structural equation model (SEM). The thickness of streamline represents the size of the effect. (B) Standard total effects, direct and indirect effects on ARGs in MPs derived from SEM. (C) Schematic diagram of the mechanisms for MPs on ARGs dissemination in mariculture sediments. MPs enriched the antibiotic resistance genes in mariculture sediments by altering specific hosts and promoting horizontal gene transfer. Hydrophobic PS MPs promotes bacterial contact and increases HGT frequency by forming a denser biofilm. PVC MPs boosted the ARG abundance mainly by inducing oxidative stress in bacteria and increasing cell membrane permeability, which enhanced the spread of ARGs through HGT among bacteria.

This study pioneers the investigation of polymer type and size-dependent promotion effects of MPs on ARG pollution in mariculture sediments, revealing the potential mechanisms of MPs enhancing the prevalence of ARGs (Fig. 6). The facilitating effects of PVC MPs on elevating ARG risks exceeded those of PS MPs, and the smaller size of MPs exhibited a more pronounced impact. PS and PVC MPs increased sediment community deterministic assembly processes, leading to ARB enrichment. The hydrophobic PS MPs enhanced ARG exchange within the plastisphere by (1) facilitating density biofilm formation and (2) increasing cellular contact. The exogenous additives and PVC MP particles synergistically promoted ARG conjugation transfer by (1) inducing oxidative stress and (2) enhancing cell membrane permeability. The hydrophobicity, specific surface area, and exogenous additives of MPs were regarded as crucial factors in promoting ARG transmission.

These findings reveal the mechanisms by which MP properties promoted the dissemination of ARGs in mariculture benthic ecosystems. This work highlighted the potential ecological risks associated with MPs in fostering the diffusion of antibiotic resistance. In coastal mariculture ecosystems, MPs acted as critical vectors for the transmission of ARGs and pathogens through the food chain, posing significant threats to the safety and sustainability of the mariculture industry. To address this issue, improving polymer manufacturing processes to reduce MP release and/or optimizing the composition of plastic additives could effectively mitigate the co-selective pressure of MPs on ARGs. This, in turn, could help manage the spread of ARGs in mariculture environments. In summary, this study underscores the urgent need to address the dual challenges of MP and ARG pollution in mariculture settings, which raises an alarm for the green and sustainable development of the mariculture industry.

## CRediT authorship contribution statement

**Yifan Liu:** Writing – original draft, Visualization, Validation, Formal analysis, Data curation. **Liuqingqing Liu:** Writing – review & editing, Investigation, Formal analysis. **Xiao Wang:** Investigation, Formal analysis. **Mengying Shao:** Validation, Investigation. **Zihan Wei:** Visualization, Investigation. **Lina Wang:** Investigation. **Bing Li:** Validation, Methodology. **Chenguang Li:** Formal analysis, Validation. **Xianxiang Luo:** Validation, Resources. **Fengmin Li:** Validation, Resources, Project administration. **Hao Zheng:** Writing – review & editing, Validation, Supervision, Resources, Project administration, Funding acquisition, Conceptualization.

## Declaration of competing interest

The authors declare that they have no known competing financial interests or personal relationships that could have appeared to influence the work reported in this paper.
