# Supplementary material for: Microplastics enhance the prevalence of antibiotic resistance genes in mariculture sediments by enriching host bacteria and promoting horizontal gene transfer
Source: Eco Environ Health. 2025 Jan 30;4(1):100136. doi: 10.1016/j.eehl.2025.100136 (PMC11883372; doi:10.1016/j.eehl.2025.100136)
Supplement: Multimedia component 1 [file mmc1.docx]

***Supplementary Information***

**Microplastics enhance the prevalence of antibiotic resistance genes in** **mariculture sediments by enriching host bacteria and promoting horizontal gene transfer**

Yifan Liu^a^, Liuqingqing Liu^a,b,^*, Xiao Wang^c^, Mengying Shao^a^, Zihan Wei^a^, Lina Wang^a^, Bing Li^d^, Chenguang Li^a^, Xianxiang Luo^a,b^, Fengmin Li^a,b^, Hao Zheng^a,b,^*

*^a^Institute of Coastal Environmental Pollution Control, College of Environmental Science and Engineering, Key Laboratory of Marine Environment and Ecology, Ministry of Education, Frontiers Science Center for Deep Ocean Multispheres and Earth System, Ocean University of China, Qingdao 266100, China*

^b^*Sanya Oceanographic Institution, Ocean University of China, Sanya 572000, China*

^c^*Marine Agriculture Research Center, Tobacco Research Institute, Chinese Academy of Agricultural Sciences, Qingdao 266101, China*

^d^*School of Energy and Environmental Engineering, University of Science and Technology Beijing, Beijing 100083, China*

*Corresponding authors: [llqq@ouc.edu.cn](mailto:llqq@ouc.edu.cn) (L. Liu); [zhenghao2013@ouc.edu.cn](mailto:zhenghao2013@ouc.edu.cn) (H. Zheng)

Name of the journal: ***Eco-Environment & Health***

Date of the document prepared: *May 13, 2024*

Number of pages:52

Number of texts:8

Number of tables:13

Number of figures:12

**Table of Contents**

**[Text S1.](#_Toc1383)** [Preparation of MPs 4](#_Toc1383)

**[Text S2.](#_Toc27648)** [Characterization of MPs 4](#_Toc27648)

**[Text S3.](#_Toc17863)** [Characteristics of MPs 8](#_Toc17863)

**[Text S4.](#_Toc31849)** [Maricultural sediment microcosms 10](#_Toc31849)

**[Text S5.](#_Toc1682)** [Determination of antibiotics and analysis of sediment physicochemical properties 12](#_Toc1682)

**[Text S6.](#_Toc12313)** [Quantification of biofilm biomass on MPs 13](#_Toc12313)

**[Text S7.](#_Toc9450)** [Assessing the role of MPs and leachates on conjugative transfer of ARGs 14](#_Toc9450)

**[Text S8.](#_Toc22009)** [Statistical analysis 16](#_Toc22009)

**[Table S1.](#_Toc10555)** [Literature review of the MP types, particle sizes, and abundance in different research. 18](#_Toc10555)

**[Table S2.](#_Toc32089)** [Selected surface elements and functional groups of MP samples. 19](#_Toc32089)

**[Table S3.](#_Toc21441)** [Selected physical properties of MP samples. 20](#_Toc21441)

**[Table S4.](#_Toc25327)** [Available heavy metal and nutrient contents in plastic leachates. 21](#_Toc25327)

**[Table S5.](#_Toc7713)** [Main components of MPs leachate determined by SPME combined with GC-MS in an electron impact ionization mode (70 eV) (matching ratio > 80%). 22](#_Toc7713)

**[Table S6.](#_Toc4748)** [The selected properties of the sediment used in the present study. 24](#_Toc4748)

**[Table S7.](#_Toc25271)** [Literature review of the concentrations of tetracycline (TC) and sulfamethoxazole (SMX) in different maricultural environments. 25](#_Toc25271)

**[Table S8.](#_Toc19360)** [Literature review of the abundance of ARGs and MGEs in different marine sediments. 26](#_Toc19360)

**[Table S9.](#_Toc8087)** [Standard curves of the target genes. 28](#_Toc8087)

**[Table S10.](#_Toc26316)** [Primers sequences and PCR reaction conditions for the target genes. 29](#_Toc26316)

**[Table S11.](#_Toc14927)** [Analyses on the inter-group and within-group differences and similarities in bacterial community structure. 31](#_Toc14927)

**[Table S12.](#_Toc11535)** [Microbial assembly processes neutral model statistics. 32](#_Toc11535)

**[Table S13.](#_Toc4837)** [Potential ARG bacterial hosts identified by the co-occurrence network analysis. 33](#_Toc4837)

**[Fig. S1.](#_Toc21843)** [Characteristics of MPs. 35](#_Toc21843)

**[Fig. S2.](#_Toc26541)** [XRD spectra and thermogravimetric curves of PVC and PS. 36](#_Toc26541)

**[Fig. S3.](#_Toc17249)** [Effect of MPs on sediment physicochemical parameters. 37](#_Toc17249)

**[Fig. S4.](#_Toc22763)** [The concentrations of tetracycline (TC) and sulfamethoxazole (SMX) in sediment overlying seawater and sediment pore water during aging period. 38](#_Toc22763)

**[Fig. S5.](#_Toc15245)** [Absolute abundance of target six ARGs and two MGEs (intI1 and tnpA) in sediment at aging phase. Spearman’s correlation heat map of the abundances of target ARGs and MGEs during the antibiotic aging period. 39](#_Toc15245)

**[Fig. S6.](#_Toc4696)** [Heat map representation of z-score transformed the absolute and relative abundance of target ARGs in the marine sediments. 40](#_Toc4696)

**[Fig. S7.](#_Toc6289)** [Heatmap analysis of taxonomic to phenotypic mapping of the 16S rRNA sequenced genes in sediments by BugBase and Tax4Fun. 41](#_Toc6289)

**[Fig. S8.](#_Toc4615)** [Contents of available Cu, Cd, Cr, Pb, Zn, and total heavy metal in the sediments. 42](#_Toc4615)

**[Fig. S9.](#_Toc15729)** [Heatmap of the microbial community composition at genus level. 43](#_Toc15729)

**[Fig. S10.](#_Toc32574)** [Ratio of donor and transconjugant colonization on the MP surfaces. 44](#_Toc32574)

**[Fig. S11.](#_Toc2917)** [The relative abundances of total MGEs, intI1, and tnpA in the marine sediments, and the linear regression between the abundance of ARGs and intI1 and tnpA in the sediments 45](#_Toc2917)

**[Fig. S12.](#_Toc22841)** [Effect of MPs on the conjugative transfer frequency of plasmid RP4 between the donor](#_Toc22841) *[P. putida](#_Toc22841)* [KT2440 and recipient](#_Toc22841) *[E. coli](#_Toc22841)* [NK5449 in a 30-mL mating system. 46](#_Toc22841)

***Text S1. Preparation of MPs***

Commercial PS granules (1 cm × 1 cm × 0.50 cm) and PVC pipes (external diameter 20 mm, thickness 2 mm) were purchased from Yangli Electromechanical Technology Co. (Shanghai, China) and Weihai Huana Plastics Co. (Shandong, China), respectively. To mimic the fragmented MPs from larger plastic items by the naturally physical weathering (e.g., winds, wave mechanical abrasion) in oceans, the pristine MPs were prepared by using an automatic sample freezer grinder (FSTPRP-24, Jingxin, China) ([Hämer et al. 2014](#_ENREF_20), [Wang et al. 2020c](#_ENREF_69)). In brief, the PVC pipes were cut into 1 cm × 1 cm pieces, and then PVC pieces or PS granules were washed five times with Milli-Q water and three times with anhydrous ethane to avoid possible airborne dust contamination. Afterward, the plastic particles were air-dried at 20 °C for 24 h, and were ground into fragments by freeze milling (2 cycles of 2 min at 15 strikes per second) in liquid nitrogen using a ball grinder (FSTPRP-24, Jingxin, China). The milled MPs were sequentially passed through the sieve with a mesh of 8, 16, 150, and 300 to obtain the PVC or PS MPs with the uniform size of 0.5–2.0 mm and 20–120 μm, respectively ([Wang et al. 2020c](#_ENREF_69)). All the obtained MPs were stored in the dark at 4 °C until further experiment.

***Text S2. Characterization of MPs***

***Morphology and size distribution analysis:*** Scanning electron microscopy (SEM, S-4800, Hitachi, Japan) was employed to observe the morphological properties of PS and PVC MPs. For SEM analysis, MPs were suspended in 50% ethanol solution, and 50 μL of MPs suspension was dropped onto an aluminum foil (1 cm × 1 cm) that was directly adhered onto the carbon (C) conductive tape, then deposited and air-dried before the observation. Laser scanning confocal microscope (LSCM, Nikon A1^+^, Nikon, Japan) and Image J software (<http://imagej.net/>) were used to observe and calculate the particle size distribution of the two big sizes of MPs (PS-B and PVC-B). Briefly, PS-B or PVC-B were added to anhydrous ethanol and uniformly dispersed by ultrasonication at 28 kHz and 20 ℃ for 30 min. Then 20 μL of the uniformly dispersed solution was dropped onto a clean coverslip, air dried and imaged by the LSCM. Then the size distribution of MPs was calculated based on more than 1000 particles with the Image J software ([Lu et al. 2017](#_ENREF_37), [Paes et al. 2022](#_ENREF_44)).

***Surface area, pore volume and average pore diameter:*** The pore and specific surface properties were determined by Quantachrome Autosorb-1 (Quantachrome, USA) with N_2_ adsorption isotherm at 330 K after degassing for 24 hours at 65 °C ([Li et al. 2020](#_ENREF_32)). The N_2_-derived surface area (SA_N2_), pore volume (V_m_, cm^3^ g^−1^) and average pore diameter (AD, nm) were calculated using the density functional theory (DFT) model ([Zhao et al. 2022](#_ENREF_85)).

***Elemental composition, functional group analysis and crystalline structure:*** Surface chemistry was analyzed by ESCALAB 250X X-ray photoelectron spectroscopy (XPS, Thermo Fisher Scientific, USA) with Al–Kα radiation (hv = 1486.6 eV) was used to analyze the surface chemical composition including C, N, O, P, and Ca and functional groups of MPs ([Wang et al. 2020c](#_ENREF_69)). The vacuum degree of the analysis room was 5 × 10^−10^ Pa, working voltage 12 kV, and filament current 6 mA. Signal accumulation was performed in 5 cycles. The test passing-energy full-spectrum was 100 eV, narrow spectrum was 50 eV, step length was 0.05 eV, and calibration was performed using the combined energy standard of C1s 284.80 eV, and the XPS spectra were analyzed by XPSPEAK41 software. The baseline correction and background subtraction of XPS survey plots were carried out using Origin 2023 software. Attenuated total reflection Fourier transformed infrared spectroscopy (ATR-FTIR, UATR Two, PerkinElmer, USA) was employed to detect the functional groups in the range of 400–4000 cm^−1^ with 4 cm^−1^ resolution. X-ray diffraction (XRD) measurement was carried out on a Bruker D8 Advance diffraction spectrometer with Cu-Ka radiation (λ = 1.54′Å) over the range 10–70° (2θ) at a scan speed of 2° (2θ) per minute ([Ur Razzaq et al. 2023](#_ENREF_61)).

***Surface charge and hydrophilicity*:** The surface charges of PS and PVC MPs were determined by a Zetasizer (Nano-ZS90, Malvern Instruments, UK). Prior to the measurement, MPs were dispersed in ultrapure water (pH 7.02) and filtered natural seawater (20°C, salinity 36‰, pH 8.3), respectively, and then sonicated for 60 min with an applied ultrasound of 50 W and an energy of 1.8 × 10^5^ J ([Wang et al. 2020a](#_ENREF_66)). In order to assess the surface hydrophilicity of MPs, an optical contact angle meter (JY-82B Kruss DSA, KRÜSS GmbH, Hamburg, Germany) and DROPImage Advanced software (Rame-Hart, Succasumna, NJ, USA) were used to determine and calculate the contact angles for water (*θ*_W_) according to a drop contact angle method ([Chen et al. 2021](#_ENREF_10)). A minimum of ten repeated measurements were performed for *θ*_W_ at different positions of each MPs in triplicate at room temperature.

***Thermogravimetric analysis:*** Thermogravimetric analysis (TGA) of MPs was performed using a synchronous thermal analyzer system (Netzsch STA 449 C, Bavaria, Germany) to examine their thermal behavior and quantify their weight loss. Briefly, 5–10 mg of the dried MPs were placed in a platinum crucible and then tested under the following conditions: temperature 25–600 °C, heating rate 10 °C/min, nitrogen flow 50 mL/min ([Chen et al. 2022](#_ENREF_7)).

***MPs leachate analysis*:** To obtain the corresponding leachates, 1.75 g of PS or PVC MPs were added into a 40-mL polytetrafluoroethylene (PTFE) headspace bottles containing 35 mL Milli-Q water and shaken in an incubator at 150 rpm and 20 ℃ in the dark for 60 d as those in the maricultural sediment microcosms experiments. Then all the suspensions were filtered through a 0.22-μm PTFE membrane (Whatman, U.S.). The organic chemicals in the leachates were determined using a solid-phase micro-extraction (SPME) method combined with a gas chromatography-mass spectrometer (GC-MS) in an electron impact ionization mode (70 eV) ([Si et al. 2016](#_ENREF_52)). To avoid chemical contamination of plastic wares, all the glassware used in this study was soaked overnight in K_2_CrO_7_/H_2_SO_4_ solution (1:1, v/v), sequentially rinsed with dichloromethane, acetone, n-hexane, and Milli-Q water for five times, and then baked at 450 °C for 5 h. A given amount (30 mL) of plastic leachate was added into a 50-mL glass vial, and a SPME stir bar (20 mm in length, coated with 1 mm polydimethylsiloxane (PDMS) film and anhydrous sodium chloride) was added before the vial was crimped with a PTFE-coated silicone septum cap. The vials were stirred at 360 rpm on a magnetic stirring apparatus (IKA, Germany) at 20 °C for 2 h. Then, the stir bars were taken out from the vials with clean tweezers, rinsed with Milli-Q water three times, and cleansed with absorbent papers (Kimberly-clark professional, China). Subsequently, the stir bars were placed in glass tubes containing 250 μL acetonitrile/methanol (2:3, v/v) solvent and were subjected to ultrasonic extraction (20 °C, 40 Hz, 20 min) with methanol (99.5% purity) thrice. The obtained solvents were diluted to 1 mL with methanol (99.5% purity), and were then analyzed by Agilent 5975 GC-MS equipped with an Agilent 300 C18 capillary column (5% phenyl methyl siloxane; Agilent technologies, USA) and an Agilent 5975C mass spectrometer (Agilent Technologies, USA). The samples were injected with an inlet temperature of 300 °C. The oven temperature was initiated at 70 °C for 2 min, elevated to 150 °C at 25 °C min^-1^ , to 170 °C at 3 °C min^-1^ , to 185 °C at 30 °C min^-1^ , to 195 °C at 3 °C min^-1^ , to 225 °C at 60 °C min^-1^, and finally elevated up to 280 °C at 8 °C min^-1^ for 10 min. Helium gas was used as a carrier with a rate of 1 mL min^-1^ ([Si et al. 2016](#_ENREF_52)). The obtained data were matched with the MS database of approximately 500 plastic additives, which was established based on information provided by the SpecialChem website (https://polymer-additives.specialchem.com) ([Chen et al. 2023](#_ENREF_9)).

The contents of heavy metals including Cu, Zn, Cr, Pb and Cd were measured by an inductively coupled plasma mass spectrometry (ICP-MS, NexION 350, PerkinElmer, USA). Total organic carbon (TOC) content was measured in 0.5 M K_2_SO_4_ (1:5, w/v) using a TOC autoanalyzer (TOC-Vario, Elementar, Germany). Available phosphorus (AP) content was determined using molybdenum antimony anti-colorimetric method. The concentrations of nitrate nitrogen (NO_3_^-^-N) and ammonium nitrogen (NH_4_^+^-N) in sediment were determined following standard methods. NO_3_^-^-N was extracted using the calcium chloride method and quantified via the Griess reaction, while NH_4_^+^-N was extracted by KCl and quantified using the indophenol blue method.

***Text S3. Characteristics of MPs***

The scanning electron microscopy images showed that the surfaces of small MPs were much rougher than the big ones (Fig. S1A). PS-S exhibited layered structures, whereas PVC-S exhibited irregular surfaces with multiple cracks along the shearing edges. The average size was 59.4 ± 25.3 μm for PS-S, 54.2 ± 33.4 μm for PVC-S, 1.10 ± 0.35 mm for PS-B, and 1.01 ± 0.33 mm for PVC-B. The attenuated total reflection Fourier-transform infrared spectroscopy spectra of four MPs align with the standard spectra of PS and PVC polymers (Fig. S1B), which is in line with the experimental design expectations. Additionally, the peaks at 873 cm^-1^ assigned to CaCO_3_ and X-ray diffraction spectra showed a sharp (*hkl*) peak at 29.4° in PVC (Fig. S1A), further confirming the presence of CaCO_3_, which could enhance PVC pipes’ tensile strength. X-ray photoelectron spectroscopy analysis revealed that PS MPs surfaces were primarily composed of C (98.0%–99.0%), while the PVC MPs surfaces were composed of C (47.1%–51.0%), O (6.8%–15.0%), Ca (1.5%–3.9%), P (1.8%–5.3%), and Cl (31.8%–31.9%) (Table S7), further confirming the presence of endogenous Ca- or P-containing additives in PVC. The O/C ratio and oxygen-containing functional groups (C-OH and -CO_3_^2-^) of PVC MPs were significantly higher than those of PS MPs (Fig. S1C, Table S2). The surface elements and functional groups on MPs generally determine their surface charge and hydrophobicity ([Luo et al., 2023](#_ENREF_39)). Accordingly, PVC-S carried a stronger negative charge (-9.6 ± 1.7 mV) than that of PS-S (-7.5 ± 0.6 mV) in the natural seawater (Table S3). Additionally, the contact angle of PVC MPs (83–89°) was significantly lower than PS MPs (103–104°) (Fig. S1D), suggesting the higher hydrophilicity of PVC MPs. Moreover, thermogravimetric analysis revealed that the decompositions of PVC MPs were more complex than those of PS MPs (Fig. S1B, S1C), further confirming that PVC MPs contain more complex additives than PS MPs.

The contents of detectable heavy metals in the four MPs followed an order of Cu > Zn > Cr > Pb > Cd (Table S4). The total contents of heavy metals released by PVC MPs were significantly higher than those of PS MPs, while small MPs released more heavy metals than big MPs. This might be due to the larger surface area of small MPs (1.18–1.22 m^2^ g^-1^) than the big MPs (0.653–0.746 m^2^ g^-1^) (Table S3). Heavy metals such as Cu, Zn, and Cr are crucial catalysts or reaction mediators in polymerization reactions and can mitigate plastic aging, thereby extending plastics’ lifespan ([Gunaalan et al. 2020](#_ENREF_17)). Additionally, the nutrient contents, including N, P, and total organic carbon (TOC), determine the species, abundances, and intraspecific and interspecific interaction ([Sheridan et al. 2022](#_ENREF_51)). In comparison to PS-S, PVC-S released higher levels of TOC, NO_3_-N, and TP (Table S4). The MPs derived TOC may serve as carbon sources to microorganisms ([Sheridan et al. 2022](#_ENREF_51)), and the organic chemicals as pollutants can pose stress on bacteria, resulting in the changes in bacterial community structure and function ([Wu et al. 2023](#_ENREF_72)). Hence, a gas chromatography-mass spectrometer was employed for analysis of organic compounds in the leachates (Table S5). The results showed that antioxidant 2246 and release agent n-hexadecenoic acid, extensively applied in various industries ([Gunaalan et al. 2020](#_ENREF_17)), were found in all four MPs. Plasticizer (bis(2-ethylhexyl) phthalate) (DEHP) was detected in PS MPs leachates, while synthetic intermediates (dichloroacetic acid), fungicide (benzothiazole), surfactants (tridecyl ester and 1-dodecanol), plasticizer BPA and P-containing flame retardant (tris(2-chloroethyl) phosphate (TCEP)) were leached from PVC MPs (Fig. S1E). Due to being directly added to the commercial products, demolding agents, DEHP, BPA, and P-containing flame retardants exhibit weak binding with the polymers ([Luo et al. 2023](#_ENREF_39)), allowing them to easily migrate into the environment. These differences in physicochemical properties will lead to distinct roles in ARGs proliferation and dissemination in the mariculture sediments.

***Text S4.*** ***Maricultural sediment*** ***microcosms***

The schematic diagram of microcosm experiment design is presented in Fig. 1B. It included an antibiotic aging period for 60 d and a MP incubation period for another 60 d. The natural surface marine sediments (0–20 cm) were collected from an aquaculture farm (120.30ºE, 36.19ºN) in Jiaozhou Bay, a typical mariculture area in China. The collected sediments were air-dried, pulverized, and sieved through a 2-mm mesh, and then homogeneously mixed and stored in dark prior to the following laboratory experiments. The sediment properties including pH, electrical conductivity (EC), the contents of total phosphorus (TP), available P (AP), NH_4_^+^-N, and extractable heavy metals (Cu, Zn, Cr, Pb and Cd) are listed in Table S2.

To mimic the scenario of heavy antibiotic contamination in mariculture sediments, 200 g of the air-dried sediment was added in a 500-mL conical flask filled with 300 mL of natural seawater (pH 8.0 and salinity 31.6‰) containing the expected concentrations of antibiotics (10 mg L^-1^ tetracycline (TC) and 5 mg L^-1^ sulfamethoxazole (SMX)), which are commonly used in mariculture and animal husbandry (Table S3). There were 42 conical flasks, wrapped in aluminum, for the antibiotic treatments and five flasks for the blank treatments without antibiotics (Fig. 1B). Additionally, the filtered natural seawater was added to the flasks twice a week to compensate for the evaporation loss of seawater. For the whole aging period with antibiotics, the sediments were cultivated in an incubator at 20 °C in the dark for 60 d. To maintain a relatively stable concentration of antibiotics within the sediment, TC was supplemented at the mid-aging stage (30 d) to achieve 10 mg L^-1^ in the overlying seawater ([Han et al. 2017](#_ENREF_22)). Aseptic syringes were used to collect overlying seawater samples, and wooden spoons were used to randomly collect the sediment samples for measuring the abundance of ARGs in the sediment and the concentrations of TC and SMX in the sediment pore water and overlying water.

After 60 d of aging with the two antibiotics, each of PVC and PS MPs was separately added into the sediments at the rates of 0.1% and 1% (w/w) (Fig. 1B). The adding rates of MPs (0.1% and 1% w/w) were selected according to their environmentally relevant levels (Table S1). In total, nine treatment groups were setup, consisting of eight groups exposed with the two MPs and one control group without MPs (CK). The groups treated with MPs were designated as follows: PS-S-L, PS-S-H, PS-B-L, PS-B-H, PVC-S-L, PVC-S-H, PVC-B-L and PVC-B-H, where -S and -B labeled the small and big size of MPs, and -L and -H pointed out the relatively lower (0.1%) and higher rate (1%) of the added MPs. Each of the treatments was set up with four replicates. All the flasks were kept consistent with those of the antibiotic aging period (Fig. 1B). After 60 d of cultivation, the sediments were sampled and homogenized. A portion of the collected sediments was stored at -80 °C for determining ARG and MGE abundance and analyzing microbial community, and the other portion was air-dried for determining physiochemical properties, including pH, electrical conductivity (EC), total organic carbon (TOC), dissolved organic carbon (DOC), TP, AP, total nitrogen (TN), NH_4_^+^-N, and the extractable heavy metal (Cu, Zn, Cr, Pb and Cd) contents (Text S3).

***Text S5. Determination of antibiotics and analysis of sediment physicochemical properties***

Tetracycline (TC) and sulfamethoxazole (SMX) in the seawater and sediment pore water were extracted by centrifugation at 2600 *g* for 10 min, and then supernatant (seawater) was filtered using a 0.2-µm syringe cartridge filter ([Pu et al. 2022](#_ENREF_46), [Wei et al. 2014](#_ENREF_71)). The concentrations of TC and SMX in the seawater, and sediment pore water were measured using a 1260 high-performance liquid chromatograph (HPLC, Agilent, USA) equipped with UV-visible detector and a reverse phase C18 column (5 μm particle size, 4.5 × 250 mm). For TC detection, the wavelength of detector was 355 nm, the column temperature was 30 °C, and the mobile phase was a mixture of acetonitrile and 0.01 M oxalic acid (25: 75, v:v) at a flow rate of 1.0 mL min^-1^ ([Yu et al. 2019](#_ENREF_79)). The injection volume was 20 μL. For SMX detection, the wavelength of detector was 265 nm, the column temperature was 30 °C, and the mobile phase was a mixture of acetonitrile and 0.05 M phosphoric acid (25: 75, v:v) at a flow rate of 1.0 mL min^−1^ ([Zheng et al. 2013](#_ENREF_87)).

The physicochemical properties of the sediments including pH, electrical conductivity (EC), Dissolved organic carbon (DOC), NH_4_^+^-N, total phosphorus (TP), and AP were determined. The pH and EC of the sediment were determined using a pH meter (AB15 Fisher Scientific, USA) and a conductivity meter (TetraCon 325, Cond 3210 SET 1, WTW, Germany), respectively. NH_4_^+^-N content in sediments was determined by indophenol blue colorimetric method using a spectrophotometer (UV-3300PC, Mapada, China) at 625 nm. TP contents were quantified by the molybdenum antimony anti-colorimetric method at an 880 nm wavelength using a spectrophotometer (UV-3300PC, Mapada, China). The determination of AP in sediments was extracted with 0.5 mol L^-1^ sodium bicarbonate solution (NaHCO_3_) and quantified using the molybdenum antimony anti-colorimetric method. The available heavy metals in the sediments were extracted using the toxicity characteristic leaching procedure (TCLP) method with 0.11 mol L^-1^ acetate solution in an incubator for 18 hours ([Gu et al. 2022](#_ENREF_16)). The supernatant was centrifuged at 4000 rpm and diluted 20-fold in a 10-mL centrifuge tube, and then the determined content of the extractable Cu, Cd, Cr, Pb and Zn by inductively coupled plasma mass spectrometry (ICP-MS, NexION 350, PerkinElmer, USA) ([Xu et al. 2017](#_ENREF_74)).

***Text S6. Quantification of biofilm biomass on MPs***

To quantify biofilm formation on MPs, a modified crystal violet stain was developed by modifying an existing protocol ([Lobelle and Cunliffe 2011](#_ENREF_34), [Wang et al. 2022a](#_ENREF_63)). In brief, 1.5 mL of mating mixture of *P. putida* KT2440 and *E. coli* NK5449 containing 1.5 or 15 mg of MPs was added into each well of 24-well microplates, and then incubated at 20 °C in the dark for 144 h. During the incubation, biofilm biomass was determined using the modified crystal violet staining method at desired time interval of 1, 12, 24, 48, 72, 96, 120 and 144 h, respectively. The bacterial suspension and culture medium were removed from the well plate under aseptic conditions. Then the remaining biofilm in the well plate was washed twice with sterile water to remove free bacteria. After air-drying for 10 minutes, 500 µL of 0.1% crystal violet dye (Sinopharm Group Chemical Reagent Co. Ltd, China) was added, and the well plate was incubated in the dark for 30 minutes. The dye solution was aspirated, and the excess dye was washed away by rinsing twice with sterile Milli-Q water. Subsequently, after being air-dried, 500 µL of ethanol (95% v/v) was added to dissolve the dye within the biofilm. The absorbance at a wavelength of 595 nm was measured using a spectrophotometer, which represents the amount of biofilm in the well plate. The incubated MPs at 120 and 140 h were passed through a 0.45 μm membrane and washed twice with sterilized PBS solution, then 16S rRNA abundance on the surface of MPs was determined using the method in Text S4.

***Text S7. Assessing the role of MPs and leachates on conjugative transfer of ARGs***

To investigate the impacts of MPs on horizontal ARG transfer, an optimized intergenera conjugative transfer model was established using the donor strain *P. putida* KT2440 and the recipient strain *E. coli* NK5449 ([Li et al. 2018a](#_ENREF_30), [Wang et al. 2019](#_ENREF_70)). Briefly, 150 μL of the resuspended donor and recipient bacteria suspensions were mixed at a ratio of 1:1 and added into 30 mL LB liquid medium to make a mating mixture. To match the microcosm experiments, PS and PVC MPs were added to the mating mixture at the concentrations of 0, 1, 10 and 100 mg L^-1^, respectively. The conjugation mixtures were then cultured at 37 °C and 200 rpm for 18 h. Subsequently, the mixtures were inoculated onto selective LB agar plates supplemented with the corresponding antibiotics to screen the transconjugants (Amp^R^, Tet^R^, and Rif^R^), donors (Amp^R^ and Tet^R^), and recipients (Rif^R^). All plates were cultivated at 37 °C for 24 hours, and the colony counts of transconjugants, donors, and recipients were tallied. The plasmid transfer frequency was calculated as the ratio of transconjugant counts to recipient cell counts ([Yu et al. 2021](#_ENREF_80)).

The MPs particles without additives and their leachates were prepared to examine their effects on the RP4 plasmid-mediated conjugation transfer using the same optimized intergenera conjugative transfer model. Briefly, 1, 10, and 100 mg MPs were added to 1 L of LB liquid medium and shaken at 37 °C and 200 rpm for 18 hours. The rinsed MPs and leachate were separated by centrifugation (8000 *g*, 10 min) and filtration through a 0.22-μm PTFE membrane ([Yuan et al. 2022](#_ENREF_81)). Subsequently, 300 μL of the 1:1 donor and recipient bacterial suspensions were added to 30 mL of the MP-leachate culture medium, the rinsed MPs were added to the mating mixture at the concentrations of 0, 1, 10 and 100 mg L^-1^, respectively, and other operational procedures were same as above. Conjugation frequency was calculated as the ratio of the colony-forming units (CFU) per mL of transconjugants to the CFU per mL of recipients ([Low et al. 2022](#_ENREF_35); [Yu et al. 2021](#_ENREF_80)). The interactive effects of MPs and the corresponding leachates on the horizontal ARG transfer were assessed using the Bliss independence model ([Bliss 1939](#_ENREF_2), [Brochado et al. 2018](#_ENREF_3), [Yuan et al. 2022](#_ENREF_81)). This involved calculation of combination indices (*CI*) is shown as follows:

$CI = (E_{p} + E_{c}-E_{p} \times E_{c}) / E_{p+c}$ (1)

where $E_{p}$, $E_{c}$ and $E_{p+c}$ represents the normalized ARG transfer frequency exposed by rinsed MPs (without leachate), leachates, and non-rinsed MPs, respectively. Based on the Bliss independence model, the combined effect of MPs and leachate was considered synergistic when *CI* < 1 or antagonistic when *CI* > 1 ([Bliss 1939](#_ENREF_2)).

***Text S8.* Statistical analysis**

One-way analysis of variance (ANOVA) Student *t*-test was conducted for the analysis of significant differences among different treatments (*P* < 0.05) using Statistical Product and Service Solutions (SPSS) software (Version 25.0, SPSS Inc, Chicago, IL, USA). The alpha-diversity indices, analyses on the similarities (ANOSIM), non-metric multidimensional scaling (NMDS) analyses, and procrustes analysis were computed using the package "vegan 2.6.4" ([Wang et al. 2023a](#_ENREF_64)). Partial least squares-discriminant analysis (PLS-DA) plots were generated using the package "mixOmics 6.22.0" ([Rohart et al. 2017](#_ENREF_48)). Network analysis depicting the co-occurrence between ARGs and dominant bacterial genera (with abundance > 0.1) was performed by "Hmisc" package and visualized on Gephi software (Version 0.9.1) based on Spearman's correlation coefficient (*R* > 0.7, *P* < 0.01) ([Xu et al. 2022a](#_ENREF_73)). The neutral community model (NCM) and null model (999 randomizations) were implemented using the "Hmisc," "minpack. lm," "stats4" and "picante" packages to evaluate the impact of stochastic processes on the assembly of bacterial communities ([Fang et al. 2023](#_ENREF_14), [Zhou and Ning 2017](#_ENREF_88)). The phylogenetic community assembly processes of sediment bacteria were evaluated by calculating the beta nearest taxon index (betaNTI) using phylocom 4.2 ([Hardy 2008](#_ENREF_23)). A |betaNTI| > 2 indicated a community assembly governed by deterministic processes, whereas a |betaNTI| < 2 suggested that stochastic processes contributed most to the community assembly ([Dini-Andreote et al. 2015](#_ENREF_12)). A structural equation model (SEM) was constructed using the AOMS 21 (SPSS Inc., Chicago, IL, USA) to estimate the direct and indirect contribution of the multiple factors to the variations of ARG profiles in the sediments polluted with MPs ([Zheng et al. 2021](#_ENREF_86)). Based on the Bliss independence model, the combined effect of MPs and leachate was considered synergistic when combination indices (*CI*) < 1 or antagonistic when *CI* > 1 ([Bliss 1939](#_ENREF_2)).

**Table S1.** Literature review of the MP types, particle sizes, and abundance in different research.

| Types | Particle sizes (μm) | Adding rates or concentrations | Sample | Reference |
| --- | --- | --- | --- | --- |
| PE, PET, PVC | 50–500 | 0.5%, w/w | Sediment microcosm system | ([Zeng et al. 2023](#_ENREF_82)) |
| PE, PUF, PVC, PLA | 53–300 | 0.5%, w/w | Sediment microcosm system | ([Seeley et al. 2020](#_ENREF_50)) |
| PS, PE, PA, PVC | 30, 200, 1500 | / | Soil microcosm system | ([Zhu et al. 2022](#_ENREF_89)) |
| All | 0.1–5.0 | 598 ± 309 items/kg | Beibu Gulf | ([Xue et al. 2020](#_ENREF_76)) |
| PE, PS | 0.03–5.00 | 1674 ± 526 items/kg | Sanggou Bay, China | ([Sui et al. 2020](#_ENREF_55)) |
| PP, PE, HDPE | 0.03–5.00 | 720 ± 192 items/kg | Puducherry coast, India | ([Dowarah and Devipriya 2019](#_ENREF_13)) |
| PS | 0.16–5.00 | 15–12852 items/kg | Qinzhou Bay, China | ([Li et al. 2018b](#_ENREF_31)) |
| PS | 0.30–5.00 | 24–253 items/kg | Hiroshima Bay, Japan | ([Sagawa et al. 2018](#_ENREF_49)) |
| PE, PP | 1.48 ± 0.90 | 84 ± 57 items/kg | Hangzhou Bay, China | ([Wang et al. 2020b](#_ENREF_67)) |

Notes: PE: Polyethylene, PET: Polyethylene Terephthalate, PVC: Polyvinyl Chloride, PUF: Polyurethane Foam, PLA: Polylactic Acid, PS: Polystyrene, PA: Polyamide (Nylon), PP: Polypropylene, HDPE: High-Density Polyethylene

**Table S2.** Selected surface elements and functional groups of MP samples.

| **Samples** | **Surface elemental content (%)^α^** | | | | | | **Atomic ratio** |  | **Surface functional groups (% of total C)** | | | |  |
| --- | --- | --- | --- | --- | --- | --- | --- | --- | --- | --- | --- | --- | --- |
|  | **C** | **O** | **N** | **P** | **Ca** | **Cl** | **O/C** |  | **C–C**  **/C=C** | **C–OH** | **π–π*** | **CO_3_^2-^** |  |
| PS-S | 98.0 ± 1.2a^β^ | 0.68 ± 0.05c | 0.62 ± 0.03a | ND^γ^ | ND | ND | (6.9 ± 0.5)×10^-3^c |  | 95 ± 1.6a | ND | 4.6 ± 0.8b | ND |  |
| PVC-S | 47.1 ± 1.6c | 15.0 ± 1.7a | 0.5 ± 0.16a | 1.8 ± 1.3b | 3.9 ± 1.6a | 31.8 ± 1.9a | 0.32 ± 0.02a |  | 66 ± 1.2c | 25 ± 0.3a | ND | 8.2 ± 0.2a |  |
| PS-B | 99.0 ± 1.1a | 0.72 ± 0.06c | 0.45 ± 0.01b | ND | ND | ND | (7.3 ± 0.6)×10^-3^c |  | 93 ± 0.8a | ND | 6.3 ± 0.5a | ND |  |
| PVC-B | 54.0 ± 1.3b | 6.8 ± 1.2b | 0.5 ± 0.07a | 5.3 ± 1.2a | 1.5 ± 1.5b | 31.9 ± 1.3a | 0.13 ± 0.02b |  | 73 ± 1.0b | 24 ± 0.5a | ND | 3.3 ± 0.1b |  |

^α^ Surface elemental and composition of C functional groups were analyzed by XPS.

^β^ Different letters behind the data represented significant differences among the different MP samples (*n* = 4, *P* < 0.05).

^γ^ ND represented “not detected”.

**Table S3.** Selected physical properties of MP samples.

| **Samples** | **Size distribution (μm)** | **Contact angles (°)** | **BET surface area (****m^2^ g^-1^)** | **Pore volume (μm^3^ g^-1^)** | **Average pore Diameter (nm)** |  | **Zeta potential (mV)** ^α^ | |
| --- | --- | --- | --- | --- | --- | --- | --- | --- |
|  |  |  |  |  |  |  | **Milli-Q water** | **Natural seawater** |
| PS-S | 59.41 ± 25.27 | 103 ± 1.1a^β^ | 1.621 | 0.002236 | 4.5 |  | 7.1 ± 0.4a | -7.5 ± 0.6a |
| PVC-S | 54.20 ± 33.37 | 83 ± 3.8c | 1.476 | 0.002614 | 5.8 |  | 9.4 ± 1.4b | -9.6 ± 1.7b |
| PS-B | 1104.81 ± 349.66 | 104 ± 0.7a | 0.653 | 0.000320 | 1.8 |  | ND^γ^ | ND |
| PVC-B | 1011.99 ± 333.71 | 89 ± 2.0b | 0.746 | 0.000370 | 2.2 |  | ND | ND |

^α^ Zeta potentials were measured at 10 mg L^-1^ MPs in the Milli-Q water (pH, 5.8) and natural seawater (pH, 8.3), respectively.

^β^ Different letters behind the data represented significant differences among the different MP samples (*n* = 4, *P* < 0.05).

^γ^ ND represented “not detected”.

**Table S4.** Available heavy metal and nutrient contents in plastic leachates.

| **Nutrient and heavy metals** | | **PS-S** | **PVC-S** | **PS-B** | **PVC-B** |
| --- | --- | --- | --- | --- | --- |
| Heavy metals^α^ | Cu (μg g^-1^) | 16.4 ± 1.7b^β^ | 24.1 ± 2.0a | 1.84 ± 0.26d | 6.83 ± 1.19c |
|  | Zn (μg g^-1^) | 45.3 ± 6.47a | 44.0 ± 4.29a | 9.18 ± 1.60b | 10.6 ± 1.20b |
|  | Cr (μg g^-1^) | 1.05 ± 0.00a | 1.06 ± 0.00a | 0.19 ± 0.00b | 0.22 ± 0.00b |
|  | Pb (μg g^-1^) | 0.33 ± 0.00b | 0.39 ± 0.01a | 0.03 ± 0.00d | 0.09 ± 0.00c |
|  | Cd (μg g^-1^) | 0.10 ± 0.00a | 0.11 ± 0.01a | 0.02 ± 0.00b | 0.02 ± 0.00b |
|  | ∑Heavy metals | 63.2 ± 4.26a | 69.7 ± 2.53a | 11.3 ± 2.17c | 17.7 ± 2.56b |
| Nutrient | TOC^γ^ (mg g^-1^) | 0.12 ± 0.00b | 0.24 ± 0.01a | 0.02 ± 0.00d | 0.07 ± 0.00c |
|  | TP^γδ^ (mg g^-1^) | 0.015 ± 0.00b | 0.025 ± 0.00a | 0.00 ± 0.00c | 0.01 ± 0.00c |
|  | NO_3_^-^-N (mg g^-1^) | 0.14 ± 0.00b | 0.22 ± 0.00a | 0.01 ± 0.00d | 0.07 ± 0.00c |
|  | NH_4_^+^-N (mg g^-1^) | 0.026 ± 0.00a | 0.010 ± 0.00b | 0.00 ± 0.00c | 0.00 ± 0.00c |

^α^ Extractable heavy metals.

^β^ The different small letters represented significant difference among the different treatments (Duncan’s multiple-comparison test, *n* = 3, *P* < 0.05).

^γ^ TOC: total carbon.

^δ^ TP: total phosphorus.

**Table S5.** Main components of MPs leachate determined by SPME combined with GC-MS in an electron impact ionization mode (70 eV) (matching ratio > 80%).

| **Number** | **Samples** | **Retention time (min)** | **Compound** | **Matching ratio (%)** | **Molecular weight (amu)** | **CAS Number** | **Function** |
| --- | --- | --- | --- | --- | --- | --- | --- |
| 1 | PS | 5.594 | Cyclopentasiloxane, decamethyl- | 91 | 370.094 | 000541-02-6 | Surfactant |
| 2 |  | 7.127 | Tridecane | 95 | 184.219 | 000629-50-5 | Stabilizer |
| 3 |  | 8.285 | Tetradecane | 96 | 198.235 | 000629-59-4 | Stabilizer |
| 4 |  | 9.778 | Pentadecane | 81 | 212.25 | 000629-62-9 | Stabilizer |
| 5 |  | 11.649 | Hexadecane | 94 | 226.266 | 000544-76-3 | Stabilizer |
| 6 |  | 12.168 | Cyclooctasiloxane, hexadecamethyl- | 92 | 592.15 | 000556-68-3 | Chain extender |
| 7 |  | 12.737 | Benzene, 1,1'-(1,3-propanediyl)bis- | 86 | 196.125 | 001081-75-0 | Bonding agent |
| 8 |  | 12.779 | Cyclobutane, 1,2-diphenyl- | 87 | 208.125 | 003018-21-1 | Plasticizer |
| 9 |  | 13.646 | Benzene, 1,1'-(1,2-cyclobutanediyl)bis-, cis- | 90 | 208.125 | 007694-30-6 | Plasticizer |
| 10 |  | 14.312 | Benzene, 1,1'-(1,2-cyclobutanediyl)bis-, trans- | 91 | 208.125 | 020071-09-4 | Plasticizer |
| 11 |  | 14.828 | Cyclononasiloxane, octadecamethyl- | 92 | 666.169 | 000556-71-8 | Chain extender |
| 12 |  | 15.132 | 1,4-Diphenyl-1,3-butadiene | 92 | 206.11 | 000886-65-7 | Plasticizer |
| 13 |  | 16.125 | Phthalic acid, isobutyl 2-pentyl ester | 83 | 292.167 | 1000315-48-6 | Plasticizer |
| 14 |  | 16.196 | Dibenzo[a,e]cyclooctene | 89 | 204.094 | 000262-89-5 | Stabilizer |
| 15 |  | 17.025 | Eicosane | 95 | 282.329 | 000112-95-8 | Lubricant |
| 16 |  | 17.156 | n-Hexadecanoic acid | 96 | 256.24 | 000057-10-3 | Release agent |
| 17 |  | 18.945 | Octadecanoic acid, methyl ester | 99 | 298.287 | 000112-61-8 | Release agent |
| 18 |  | 19.559 | Tetracosamethyl-cyclododecasiloxane | 90 | 888.226 | 018919-94-3 | Stabilizer |
| 19 |  | 21.898 | Bis(2-ethylhexyl) phthalate | 68 | 390.277 | 000117-81-7 | Plasticizer |
| 20 |  | 22.176 | 2,2'-Methylenebis(4-methyl-6-tert-butylphenol) (Antioxidant 2246) | 96 | 340.24 | 000119-47-1 | Antioxidant |
|  |  |  |  |  |  |  |  |
| 1 | PVC | 5.598 | Cyclopentasiloxane, decamethyl- | 94 | 370.094 | 000541-02-6 | Plasticizer |
| 2 |  | 6.247 | 1,2-Benzisothiazole | 86 | 135.014 | 000272-16-2 | Stabilizer |
| 3 |  | 6.247 | Tris（2-chloroethyl）phosphate | 83 | 356.106 | 053044-27-2 | Flame retardant |
| 4 |  | 6.929 | Cyclohexasiloxane, dodecamethyl- | 90 | 444.113 | 000540-97-6 | Chain extender |
| 5 |  | 7.127 | Tridecane | 95 | 184.219 | 000629-50-5 | Stabilizer |
| 6 |  | 7.923 | Dodecanal | 87 | 184.183 | 000112-54-9 | Stabilizer |
| 7 |  | 8.285 | Tetradecane | 96 | 198.235 | 000629-59-4 | Stabilizer |
| 8 |  | 8.744 | 1-Dodecanol | 95 | 186.198 | 000112-53-8 | Plasticizer |
| 9 |  | 9.622 | Cyclopropane, nonyl- | 94 | 168.188 | 074663-85-7 | Plasticizer |
| 10 |  | 9.773 | Pentadecane | 96 | 212.25 | 000629-62-9 | Stabilizer |
| 11 |  | 9.909 | Butylated Hydroxytoluene | 91 | 220.183 | 000128-37-0 | Antioxidant |
| 12 |  | 10.989 | Cyclododecane | 91 | 168.188 | 000294-62-2 | Stabilizer |
| 13 |  | 11.649 | Hexadecane | 92 | 226.266 | 000544-76-3 | Stabilizer |
| 14 |  | 12.293 | Bisphenol A | 95 | 228.245 | 000080-05-7 | Plasticizers |
| 15 |  | 13.364 | Heptadecane | 97 | 240.282 | 000629-78-7 | Stabilizer |
| 16 |  | 14.598 | Eicosanal- | 95 | 296.308 | 002400-66-0 | Stabilizer |
| 17 |  | 14.831 | Cyclononasiloxane, octadecamethyl- | 94 | 666.169 | 000556-71-8 | Plasticizer |
| 18 |  | 15.743 | Cyclotetradecane | 94 | 196.219 | 000295-17-0 | Stabilizer |
| 19 |  | 16.121 | 1,2-Benzenedicarboxylic acid, bis(2-methylpropyl) ester | 86 | 278.152 | 000084-69-5 | Plasticizer |
| 20 |  | 16.405 | Acetic acid, chloro-, octadecyl ester | 91 | 346.264 | 005348-82-3 | Dehydrating agent |
| 21 |  | 16.571 | Nonadecane | 97 | 268.313 | 000629-92-5 | Stabilizer |
| 22 |  | 17.168 | n-Hexadecanoic acid | 99 | 256.24 | 000057-10-3 | Release agent |
| 23 |  | 17.238 | Oxirane, hexadecyl- | 93 | 268.277 | 007390-81-0 | Stabilizer |
| 24 |  | 17.622 | Eicosane | 96 | 282.329 | 000112-95-8 | Stabilizer |
| 25 |  | 17.809 | Bacteriochlorophyll-c-stearyl | 94 | 840.54 | 1000164-49-7 | Photosensitizer |
| 26 |  | 18.677 | Heneicosane | 94 | 296.344 | 000629-94-7 | Stabilizer |
| 27 |  | 18.945 | Octadecanoic acid, methyl ester | 99 | 298.287 | 000112-61-8 | Stabilizer |
| 28 |  | 20.787 | Tetracosamethyl-cyclododecasiloxane | 93 | 888.226 | 018919-94-3 | Plasticizer |
| 29 |  | 22.057 | Tetracosane | 98 | 338.391 | 000646-31-1 | Stabilizer |
| 30 |  | 22.176 | 2,2'-Methylenebis(4-methyl-6-tert-butylphenol) (Antioxidant 2246) | 96 | 340.24 | 000119-47-1 | Antioxidant |
| 31 |  | 23.269 | Pentacosane | 92 | 352.407 | 000629-99-2 | Stabilizer |
| 32 |  | 25.763 | Tetratriacontane | 90 | 478.548 | 014167-59-0 | Stabilizer |

**Table S6.** The selected properties of the sediment used in the present study (mean ± SD, *n* = 3).

| Properties | Sediment |
| --- | --- |
| pH | 8.60 ± 0.15 |
| EC^α^ (μs cm^-1^) | 6.26 ± 0.96 |
| TP^β^ (mg kg^-1^) | 221 ± 35.1 |
| AP^γ^ (mg kg^-1^) | 2.70 ± 0.10 |
| NH_4_^+^-N (mg kg^-1^) | 16.0 ± 0.65 |
| Cu^ε^ (mg kg^-1^) | 2.20 ± 0.05 |
| Cd (mg kg^-1^) | 0.01 ± 0.002 |
| Cr (mg kg^-1^) | 0.01 ± 0.001 |
| Pb (mg kg^-1^) | 0.16 ± 0.02 |
| Zn (mg kg^-1^) | 1.16 ± 0.15 |

^α^ EC: electrical conductivity.

^β^ TP: total phosphorus.

^γ^ AP: available phosphorus.

^ε^ Extractable heavy metals including Cu, Cd, Cr, Pb, and Zn.

**Table S7.** Literature review of the concentrations of tetracycline (TC) and sulfamethoxazole (SMX) in different maricultural environments.

| **Sample** | **Surface water (****μg L^-1^)** | |  | **Sediments (****μg** **kg^-1^** **dry weight)** | | **Reference** |
| --- | --- | --- | --- | --- | --- | --- |
|  | **TC** | **SMX** |  | **TC** | **SMX** |  |
| Jiaozhou Bay (China) | / | / |  | / | 0.025−3.5 | ([Wang et al. 2023b](#_ENREF_65)) |
| Fengjia Bay (China) | 0.01−1.34 | / |  | / | / | ([Qin et al. 2023](#_ENREF_47)) |
| South China Sea (China) | ND−2.30 | 4 × 10^-4^−5.9 × 10^-3^ |  | / | / | ([Wang et al. 2022b](#_ENREF_68)) |
| Liaodong Bay (China) | / | 0.43 |  | / | / | ([Wang et al. 2022b](#_ENREF_68)) |
| Korea Strait (South Korea) | / | 1.53 × 10^-3^−0.13 |  | / | / | ([Wang et al. 2022b](#_ENREF_68)) |
| Guangzhou (China) | ND-0.85 | / |  | / | / | ([Wang et al. 2021](#_ENREF_62)) |
| Zhejiang province (China) | / | 130–150 |  | / | / | ([Peng et al. 2020](#_ENREF_45)) |
| Yellow Sea (China) | ND−9 × 10^-4^ | ND−1.27 |  | 5.1 × 10^-4^−7.43 | (0.09−2.55) × 10^-3^ | ([Han et al. 2020](#_ENREF_21)) |
| Jiangsu Province (China) | / | / |  | ND−2085 | / | ([Chen et al. 2020](#_ENREF_8)) |
| Southern Baltic Sea | ND | ND−0.31 × 10^-3^ |  | ND−0.014 | ND−9 × 10^-4^ | ([Siedlewicz et al. 2018](#_ENREF_53)) |
| Shandong Province (China) | ND−54.7 | 7.4−69.5 |  | 0.87−25.3 | ND−0.35 | ([Chen et al. 2017](#_ENREF_5)) |
| Hailing Bay region (China) | ND−2.5 | / |  | ND | ND | ([Chen et al. 2015b](#_ENREF_6)) |
| Gulf of Gdańsk (Poland) | / | / |  | 21−625 | / | ([Siedlewicz et al. 2014](#_ENREF_54)) |
| Viet Nam | / | 970–1270 |  | / | 6870–12600 ww^β^ | ([Le et al. 2005](#_ENREF_29)) |
| Viet Nam | / | 40–5570 |  | / | 4.77–820 ww^β^ | ([Le and Munekage 2004](#_ENREF_28)) |
| Range | ND−54.7 | ND–5570 |  | ND–2085 | ND–12600 ww |  |

^α^ ND: not detected due to due to concentration below the detection limit.

^β^ ww: wet weight of sediments.

**Table S8.** Literature review of the abundance of ARGs and MGEs in different marine sediments.

| **Sampling sites** | **ARGs/MGEs Type** | **Methodology** | **Absolute abundance**  **(copies g^-1^)** | **Relative abundance**  **(copies per 16S rRNA genes)** | **Reference** |
| --- | --- | --- | --- | --- | --- |
| Yangtze Estuary  (China) | *sul*1  *sul*2  *tet*W | qPCR | 1.74 × 10^4^−9.52 × 10^6^  2.16 × 10^3^−9.05 × 10^5^  4.58 × 10^4^−1.26 × 10^6^ | / | ([Zhang et al. 2023](#_ENREF_84)) |
| Shanghai  (China) | 510 subtypes | Metagenomic | 0−29,716 | / | ([Yang et al. 2023](#_ENREF_78)) |
| Jiaozhou Bay  (China) | 15 ARGs subtypes | qPCR | Average values of more than  4.8 × 10^7^ | / | ([Wang et al. 2023b](#_ENREF_65)) |
| Bangladesh coastal aquaculture systems | 160 ARGs subtypes | High-throughput qPCR ARG SmartChip | / | 0.05–0.06 | ([Lassen et al. 2022](#_ENREF_27)) |
| Thailand coastal aquaculture systems | 160 ARGs subtypes | High-throughput qPCR ARG SmartChip | / | 0.03–0.13 | ([Lassen et al. 2022](#_ENREF_27)) |
| Jiangsu Province (China) | *sul*1,  *tet*A,  *qnr*S | Metagenomic | 49.4 × 10^8^  9.25 × 10^6^  5.49 × 10^6^ | / | ([Xu et al. 2022b](#_ENREF_75)) |
| Thailand coastal  aquaculture region | 283 ARGs subtypes | High-throughput qPCR (HT-qPCR) | 10^6^−10^7^  10^6^−10^8^  10^6^−10^7^  10^7^−10^8^  10^5^−10^6^  10^6^−10^7^  10^5^−10^7^ | / | ([Thongsamer et al. 2021](#_ENREF_60)) |
| Mariana Trench | 143 ARG subtypes | Metagenomic | / | 5.24 × 10^–3^ to 2.73 × 10^–2^  copy cell^-1^ | ([Yang et al. 2021](#_ENREF_77)) |
| South Korea aquaculture region | 20 ARGs | qPCR | / | 4.24×10^−3^–1.46×10^−2^ | ([Jang et al. 2018](#_ENREF_25)) |
| China coastline mariculture region | *sul*1  *sul*2  *tet*B  *qur*A | qPCR | / | 6.87 × 10^−2^  8.99 × 10^−2^  1.57 × 10^−2^  2.15 × 10^−2^ | ([Gao et al. 2018](#_ENREF_15)) |
| Yangtze River  estuary (China) | *sul*1  *sul*2  *tet*A  *tetW* | qPCR | 10^4^−10^9^  10^3^−10^6^  10^4^−10^7^  10^4^−10^5^ | / | ([Guo et al. 2018](#_ENREF_18)) |
| Bohai Sea (China) | *sul*1  *sul*2  *tetM* | qPCR | / | 10^−4^−10^−2^  10^−4^−10^−2^  1.7×10^−4^ | ([Niu et al. 2016](#_ENREF_43)) |
| Turku archipelago Baltic Sea | 9 ARGs | qPCR | / | 10^−4^–10^−2^ | ([Muziasari et al. 2016](#_ENREF_41)) |
| Pearl River estuary  (China) | *sul*1  *sul*2 | qPCR | 10^8^−10^10^  10^8^−10^10^ | / | ([Chen et al. 2015a](#_ENREF_4)) |
| Manila Bay  (Philippines) | *sul*1  *sul*2 | qPCR | / | 10^– 3^  8×10^−4^ | ([Suzuki et al. 2013](#_ENREF_56)) |
| Baltic Sea in the Turku Archipelago | *tet*C and *tet*M  *tet*A and *tet*H | qPCR | 10^6^−10^7^  10^4^−10^5^ | 10^−4^−10^−2^  10^−5^−10^−4^ | ([Tamminen et al. 2011](#_ENREF_58)) |
| Crassostrea virginica beds (USA) | *tet*D | qPCR | 10^8^−10^10^ | / | ([Barkovskii et al. 2010](#_ENREF_1)) |
| Antarctica | *sul*1  *sul*2  *qnr*S | qPCR | 10^1^−10^3^  10^1^−10^3^  10^1^−10^3^ | / | ([Na et al. 2019](#_ENREF_42)) |
| Arctic and subarctics | *sul*1  *sul*2  *qnr*A | qPCR | / | 2.45×10^−7^−1.71×10^−6^  4.05×10^−7^−6.63×10^−6^  3.40×10^−9^−9.90×10^−7^ | ([Tan et al. 2018](#_ENREF_59)) |
| East China Sea | *tnp*A | qPCR | / | 1.89×10^−4^–1.60^−3^ | ([Lu and Lu 2020](#_ENREF_38)) |
| Bohai Sea (China) | *intІ*1 | qPCR | 2.8 ×10^5^−2.5 10^8^ | / | ([Zhang et al. 2020](#_ENREF_83)) |
| Yellow Sea coastal areas (China) | *intІ*1  *tnp*A | qPCR | 3.30 × 10^2^  3.54 × 10^3^ | / | ([Lu et al. 2022](#_ENREF_36)) |

**Table S9.** Standard curves of the target genes.

| **Number** | **Target gene** | **Linear equation** | ***R^2^*** | **Amplification**  **efficiencies (%)** |
| --- | --- | --- | --- | --- |
| 1 | *tet*M | y = -3.581x + 51.966 | 0.999 | 90.2 |
| 2 | *tet*W | y = -3.726x + 53.418 | 0.998 | 85.5 |
| 3 | *sul*1 | y = -3.438x + 52.428 | 0.998 | 95.4 |
| 4 | *sul*2 | y = -3.727x + 52.573 | 0.998 | 85.5 |
| 5 | *sul*3 | y = -3.55x + 46.81 | 0.997 | 91.3 |
| 6 | *flo*R | y = -3.58x + 53.727 | 0.999 | 90.3 |
| 7 | *qac*H01 | y = -3.467x + 50.883 | 0.996 | 94.3 |
| 8 | *Bla*_TEM_ | y = -3.6x + 54.414 | 0.999 | 89.6 |
| 9 | *amp*276 | y = -3.661x + 50.443 | 0.998 | 87.6 |
| 10 | *aad*A5 | y = -3.74x + 53.642 | 0.998 | 85.1 |
| 11 | *aad*E | y = -3.713x + 52.47 | 0.994 | 85.9 |
| 12 | *mat*A | y = -3.64x + 54.548 | 0.999 | 88.2 |
| 13 | *intІ*1 | y = -3.507x + 49.592 | 0.995 | 92.8 |
| 14 | *tnp*A | y = -3.382x + 52.789 | 0.995 | 97.5 |
| 15 | 16S rRNA | y = -3.365x + 51.731 | 0.993 | 98.3 |

**Table S10.** Primers sequences and PCR reaction conditions for the target genes.

| **Target genes** | **Gene classification** ^α^ | **Resistance mechanism** ^α^ | **Primer sequence (5′-3′)** ^γ^ | **Amplicon size**  **(bp)** | **Annealing temperature**  **(°C)** | **Reference** |
| --- | --- | --- | --- | --- | --- | --- |
| *tet*M | Tetracyclines | cellular protection | F: CATCATAGACACGCCAGGACATAT | 102 | 60 | ([Chu et al. 2021](#_ENREF_11)) |
|  |  |  | R: ATGGYTGTCGTCAGCTCGTG |  |  |  |
| *tet*W |  | cellular protection | F: GAGAGCCTGCTATATGCCAGC | 133 | 60 | ([Liao et al. 2018](#_ENREF_33)) |
|  |  |  | R: GGGCGTATCCACAATGTTAAC |  |  |  |
| *sul*1 | Sulfonamide | cellular protection | F: CACCGGAAACATCGCTGCA | 158 | 57 | ([Liao et al. 2018](#_ENREF_33)) |
|  |  |  | R: AAGTTCCGCCGCAAGGCT |  |  |  |
| *sul*2 |  | cellular protection | F: CTCCGATGGAGGCCGGTAT | 190 | 60 | ([Liao et al. 2018](#_ENREF_33)) |
|  |  |  | R: GGGAATGCCATCTGCCTTGA |  |  |  |
| *sul*3 |  | cellular protection | F: CCCATACCCGGATCAAGAATAA | 144 | 58 | ([Mao et al. 2014](#_ENREF_40)) |
|  |  |  | R: CAGCGAATTGGTGCAGCTACTA |  |  |  |
| *flo*R | Multidrug | efflux pump | F: ATTGTCTTCACGGTGTCCGTTA | 62 | 60 | ([Liao et al. 2018](#_ENREF_33)) |
|  |  |  | R: CCGCGATGTCGTCGAACT |  |  |  |
| *qac*H01 |  | efflux pump | F: GTGGCAGCTATCGCTTGGAT | 59 | 60 | ([Zhu et al. 2017](#_ENREF_91)) |
|  |  |  | R: CCAACGAACGCCCACAA |  |  |  |
| *Bla*_TEM_ | Beta Lactamase | antibiotic deactivation | F: AGCATCTTACGGATGGCATGA | 102 | 60 | ([Zhu et al. 2017](#_ENREF_91)) |
|  |  |  | R: TCCTCCGATCGTTGTCAGAAGT |  |  |  |
| *amp*276 |  | antibiotic deactivation | F: GTGGTGTCACGCTCGTCGTTTGGT | 276 | 60 | / |
|  |  |  | R: GCAACTCGGTCGCCGCATACACTATT |  |  |  |
| *aad*A5 | Aminoglycoside | antibiotic deactivation | F: ATCACGATCTTGCGATTTTGCT | 70 | 60 | ([Liao et al. 2018](#_ENREF_33)) |
|  |  |  | R: CTGCGGATGGGCCTAGAAG |  |  |  |
| *aad*E |  | antibiotic deactivation | F: GATCTTACCTTATTGCCCTTGGA | 143 | 60 | ([Guo et al. 2021](#_ENREF_19)) |
|  |  |  | R: GCGCTTGGCTTTCTTACATG |  |  |  |
| *mat*A | MLSB ^β^ | efflux pump | F: TAGTAGGCAAGCTCGGTGTTGA | 82 | 60 | ([Zhu et al. 2013](#_ENREF_90)) |
|  |  |  | R: CCTGTGCTATTTTAAGCCTTGTTTCT |  |  |  |
| *intІ*1 | MGEs | integrase | F: CCTCCCGCACGATGATC | 281 | 57 | ([Liao et al. 2018](#_ENREF_33)) |
|  |  |  | R: TCCACGCATCGTCAGGC |  |  |  |
| *tnp*A |  | transposase (IS4 group) | F: GGGCGGGTCGATTGAAA | 102 | 56.5 | ([Szekeres et al. 2017](#_ENREF_57)) |
|  |  |  | R: GTGGGCGGGATCTGCTT |  |  |  |
| 16S rRNA | 16S rRNA | NA | F: CCTACGGGAGGCAGCAG | 61 | 60 | ([Zhu et al. 2013](#_ENREF_90)) |
|  |  |  | R: TTACCGCGGCTGCTGGCAC |  |  |  |

^α^ Antibiotic classification and resistance mechanism of each ARG subtype based on the Comprehensive Antibiotic Resistance Database (https://card.mcmaster.ca/).

^β^ MLSB: macrolide-lincosamide-streptogramin B.

^γ^ F: forward primer; R: reverse primer.

**Table S11.** Analyses on the inter-group and within-group differences and similarities in bacterial community structure.

| Group | *R* | *P* value |
| --- | --- | --- |
| CK vs MPs | 0.3467 | 0.005 |
| CK vs PS | 0.8519 | 0.001 |
| CK vs PVC | 0.1585 | 0.005 |
| CK vs Big | 0.5956 | 0.002 |
| CK vs Small | 0.6089 | 0.001 |
| CK vs High | 0.3467 | 0.005 |
| CK vs Low | 0.4296 | 0.003 |
| PVC vs PS | 0.6249 | 0.003 |
| Small vs Big | 0.3526 | 0.005 |
| Low vs High | 0.2017 | 0.010 |

Notes: The *R* values close to 1 indicate a large variation between the groups. The *R* values close to or less than 0 represent that the inter-group and within-group differences are not significant. The *P* value indicates the reliability of the analysis. If *P* < 0.05, the reliability of the test is high.

**Table S12.** Microbial assembly processes neutral model statistics.

| Group | *R*^2^ | Above prediction | Neutral distribution | Below prediction | *m* |
| --- | --- | --- | --- | --- | --- |
| CK | 0.462 | 0.045 | 0.942 | 0.014 | 0.095 |
| PS-S | 0.476 | 0.044 | 0.931 | 0.024 | 0.321 |
| PS-B | 0.490 | 0.034 | 0.951 | 0.015 | 0.264 |
| PS-L | 0.485 | 0.035 | 0.949 | 0.016 | 0.287 |
| PS-H | 0.476 | 0.035 | 0.949 | 0.016 | 0.296 |
| PVC-S | 0.292 | 0.061 | 0.904 | 0.034 | 0.138 |
| PVC-B | 0.391 | 0.045 | 0.925 | 0.030 | 0.191 |
| PVC-L | 0.323 | 0.045 | 0.927 | 0.029 | 0.107 |
| PVC-H | 0.331 | 0.048 | 0.919 | 0.033 | 0.121 |

Notes: *R^2^* Value (Goodness of Fit): A higher *R^2^* value indicates that the process of community assembly is more affected by neutral process, while a higher *R^2^* value suggests a greater influence of neutral process. Delineation Type: The delineation type (above, below, or within neutral predictions) serves as a predictor for distinguishing neutral from non-neutral partitions of the metacommunity. Migration Rate (*m*): The single free parameter "*m*" describes the migration rate. It represents the random loss of an individual in a local community, which would be replaced by dispersal from the metacommunity. Therefore, "*m*" can be interpreted as a measure of dispersal limitation.

**Table S13.** Potential ARG bacterial hosts identified by the co-occurrence network analysis.

| **Phylum** | **Genus** | **Degree** | **Weighted degree** | **Closnesscentrality** | **Betweenesscentrality** | **Authority** |
| --- | --- | --- | --- | --- | --- | --- |
| Acidobacteria | *Subgroup_10* | 3 | 2.39 | 0.26 | 80.30 | 0.16 |
|  | *Occallatibacter* | 1 | 0.72 | 0.20 | 0.00 | 0.02 |
| Actinobacteria | *Egicoccus* | 1 | 0.90 | 0.19 | 0.00 | 0.00 |
|  | *Marmoricola* | 1 | 0.88 | 0.19 | 0.00 | 0.00 |
|  | *Euzebya* | 1 | 0.84 | 0.19 | 0.00 | 0.00 |
|  | *Conexibacter* | 1 | 0.76 | 0.24 | 0.00 | 0.07 |
| Bacteroidetes | *Salinimicrobium* | 3 | 2.43 | 0.26 | 80.30 | 0.16 |
|  | *Salegentibacter* | 1 | 0.72 | 0.19 | 0.00 | 0.00 |
|  | *Prevotellaceae_NK3B31_group* | 1 | 0.72 | 0.20 | 0.00 | 0.02 |
|  | *Ignavibacterium* | 1 | 0.76 | 0.22 | 0.00 | 0.01 |
|  | *Prevotellaceae_UCG-001* | 1 | 0.87 | 0.20 | 0.00 | 0.02 |
|  | *Aurantivirga* | 1 | 0.72 | 0.20 | 0.00 | 0.02 |
| Firmicutes | *Sporosalibacterium* | 1 | 0.85 | 0.20 | 0.00 | 0.02 |
|  | *Desulfitibacter* | 1 | 0.73 | 0.19 | 0.00 | 0.00 |
|  | *Caminicella* | 1 | 0.75 | 0.60 | 0.00 | 0.00 |
|  | *Pontibacillus* | 1 | 0.75 | 0.18 | 0.00 | 0.04 |
|  | *Garciella* | 1 | 0.76 | 0.19 | 0.00 | 0.00 |
|  | *Symbiobacterium* | 1 | 0.70 | 1.00 | 0.00 | 0.00 |
|  | *Vulcanibacillus* | 1 | 0.83 | 0.19 | 0.00 | 0.00 |
|  | *Lactobacillus* | 1 | 0.77 | 0.20 | 0.00 | 0.02 |
|  | *Clostridium_sensu_stricto_10* | 1 | 0.84 | 0.20 | 0.00 | 0.02 |
| Gemmatimonadetes | *Gemmatimonas* | 1 | 0.79 | 0.20 | 0.00 | 0.02 |
| Planctomycetes | *Bythopirellula* | 2 | 1.45 | 0.18 | 0.48 | 0.10 |
|  | *SM1A02* | 1 | 0.82 | 0.20 | 0.00 | 0.02 |
|  | *Planctomicrobium* | 1 | 0.90 | 0.19 | 0.00 | 0.00 |
|  | *Rubripirellula* | 1 | 0.70 | 0.22 | 0.00 | 0.01 |
| Proteobacteria | *Candidatus_Alysiosphaera* | 4 | 3.21 | 0.26 | 105.62 | 0.20 |
|  | *Bradymonas* | 4 | 3.23 | 0.25 | 62.51 | 0.19 |
|  | *Nitrococcus* | 4 | 3.08 | 0.25 | 62.51 | 0.19 |
|  | *Salinisphaera* | 3 | 2.33 | 0.19 | 10.59 | 0.11 |
|  | *Pelagibius* | 3 | 2.37 | 0.25 | 32.69 | 0.17 |
|  | *Thiohalomonas* | 2 | 1.44 | 0.25 | 697.00 | 0.01 |
|  | *Tistlia* | 2 | 1.49 | 0.30 | 385.00 | 0.08 |
|  | *Pelagibacterium* | 2 | 1.51 | 0.30 | 385.00 | 0.08 |
|  | *Pseudolabrys* | 2 | 1.46 | 0.28 | 697.00 | 0.08 |
|  | *Methylophaga* | 1 | 0.75 | 0.19 | 0.00 | 0.00 |
|  | *Haliangium* | 1 | 0.79 | 0.19 | 0.00 | 0.00 |
|  | *MSBL7* | 1 | 0.71 | 0.19 | 0.00 | 0.00 |
|  | *Marinibaculum* | 1 | 0.74 | 0.19 | 0.00 | 0.00 |
|  | *Altererythrobacter* | 1 | 0.79 | 0.19 | 0.00 | 0.00 |
|  | *Vulgatibacter* | 1 | 0.80 | 0.18 | 0.00 | 0.04 |
|  | *Dyella* | 1 | 0.81 | 0.20 | 0.00 | 0.02 |
|  | *Caenispirillum* | 1 | 0.70 | 0.60 | 0.00 | 0.00 |
|  | *Ralstonia* | 1 | 0.82 | 0.20 | 0.00 | 0.02 |
|  | *Bradyrhizobium* | 1 | 0.75 | 0.60 | 0.00 | 0.00 |
|  | *Mesorhizobium* | 1 | 0.80 | 0.22 | 0.00 | 0.01 |
|  | *Methylotenera* | 1 | 0.79 | 0.18 | 0.00 | 0.04 |
|  | *Thiogranum* | 1 | 0.82 | 1.00 | 0.00 | 0.00 |
|  | *Sedimenticola* | 1 | 0.76 | 0.19 | 0.00 | 0.00 |
| Tenericutes | *Haloplasma* | 1 | 0.79 | 0.19 | 0.00 | 0.00 |
| Verrucomicrobia | *Persicirhabdus* | 1 | 0.70 | 0.20 | 0.00 | 0.02 |
|  | *Lentimonas* | 1 | 0.74 | 0.20 | 0.00 | 0.02 |
| Unclassied | *Sva0996_marine_group* | 1 | 0.74 | 0.19 | 0.00 | 0.00 |
|  | *Candidatus_Solibacter* | 1 | 0.83 | 0.20 | 0.00 | 0.02 |
|  | *Eubacterium_coprostanoligenes_group* | 1 | 0.70 | 0.20 | 0.00 | 0.02 |
|  | *LS-NOB* | 1 | 0.71 | 0.20 | 0.00 | 0.02 |


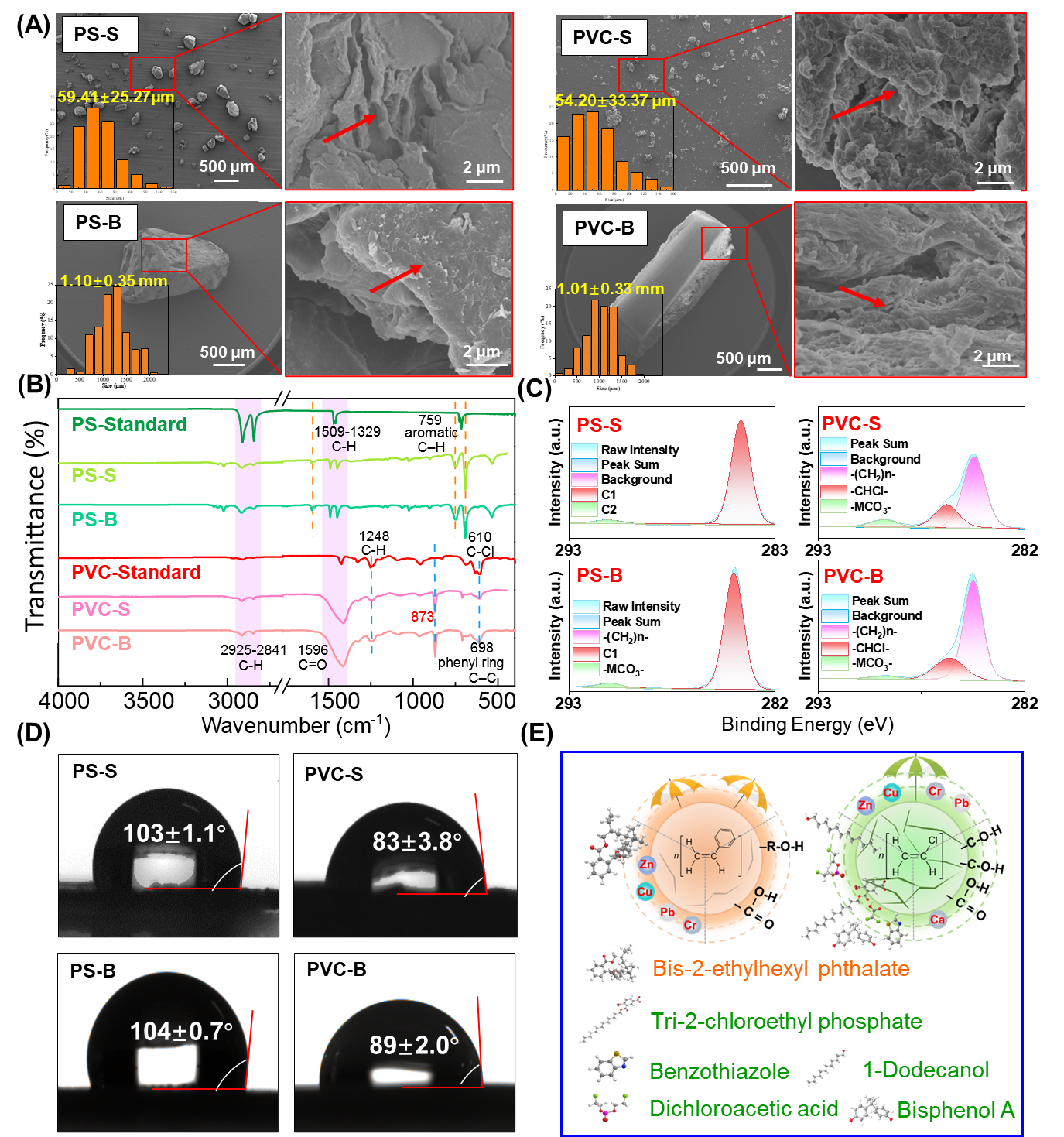


**Fig. S1.** Characteristics of MPs.

(A) SEM images of MPs and their size distribution histogram (the inset column figure). The red edged images were enlarged from the frame in the left panel. (B) FTIR spectra of the standard PS and PVC spectra and the prepared MPs. (C) XPS C1s spectra and (D) contact angles of the MPs. (E) MPs physicochemical properties and leachate composition pattern diagram. Both PS and PVC MPs leachates contain heavy metals including Ca, Cu, Cr, and Pb. PS had a more hydrophobic surface (representing by umbrella) and bis-2-ethylhexyl phthalate detected in the leachate. PVC surface contained more cracks and/or fractures and was rich in oxygen-containing functional groups and Ca elements. The PVC leachate contained tri-2-chloroethyl phosphate, 1-dodecanol, dichloroacetic acid, and benzothiazole additives.


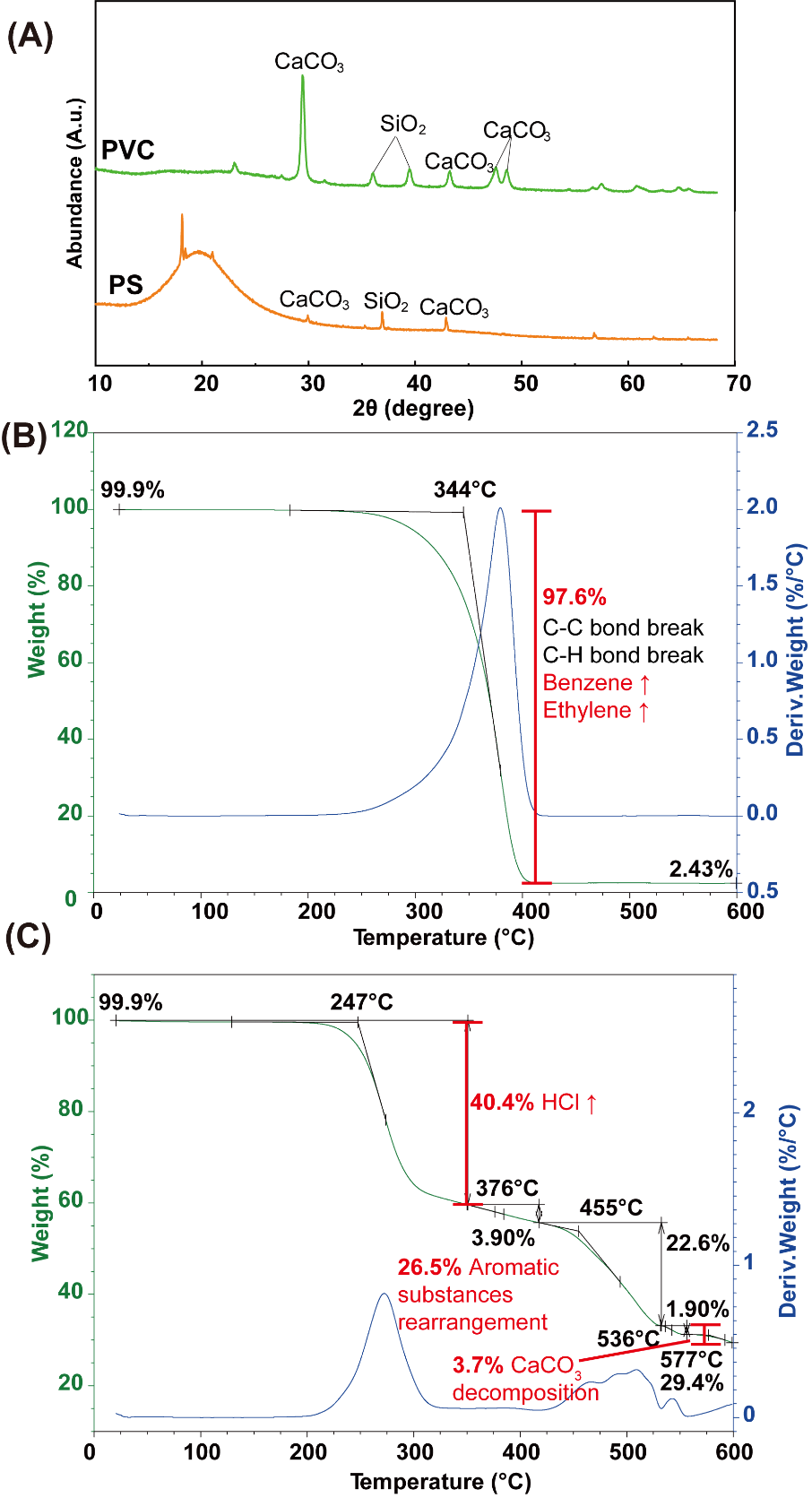


# Fig. S2. XRD spectra and thermogravimetric curves of PVC and PS.

# (A) XRD spectra of PVC and PS, thermogravimetric curves of (B) PS and (C) PVC. For the PVC sample, four prominent peaks can be observed at 29.44° (CaCO_3_), 43.26° (SiO_2_), 47.60° (CaCO_3_), and 48.62° (CaCO_3_). PS decomposed only between 344 °C and 400 °C in a nitrogen atmosphere, with a mass loss of up to 97.6%, which was mainly attributed to the formation of benzene and ethylene monomer by breakage of C**–**C bond and C**–**H bond in PS polymer ([Hu et al. 2023](#_ENREF_24)). In contrast, the decompositions of PVC occurred at 247.6–376.2 °C, 376–536 °C, and 536–600 °C, and the mass loss were 40.4%, 26.5%, and 70.6%, respectively. These mass losses are mainly due to the release of HCl gas, the rearrangement of aromatic substances, and the decomposition of CaCO3 ([Kaal et al. 2023](#_ENREF_26)), further confirming that PVC MPs contained more complex additives compared to PS.


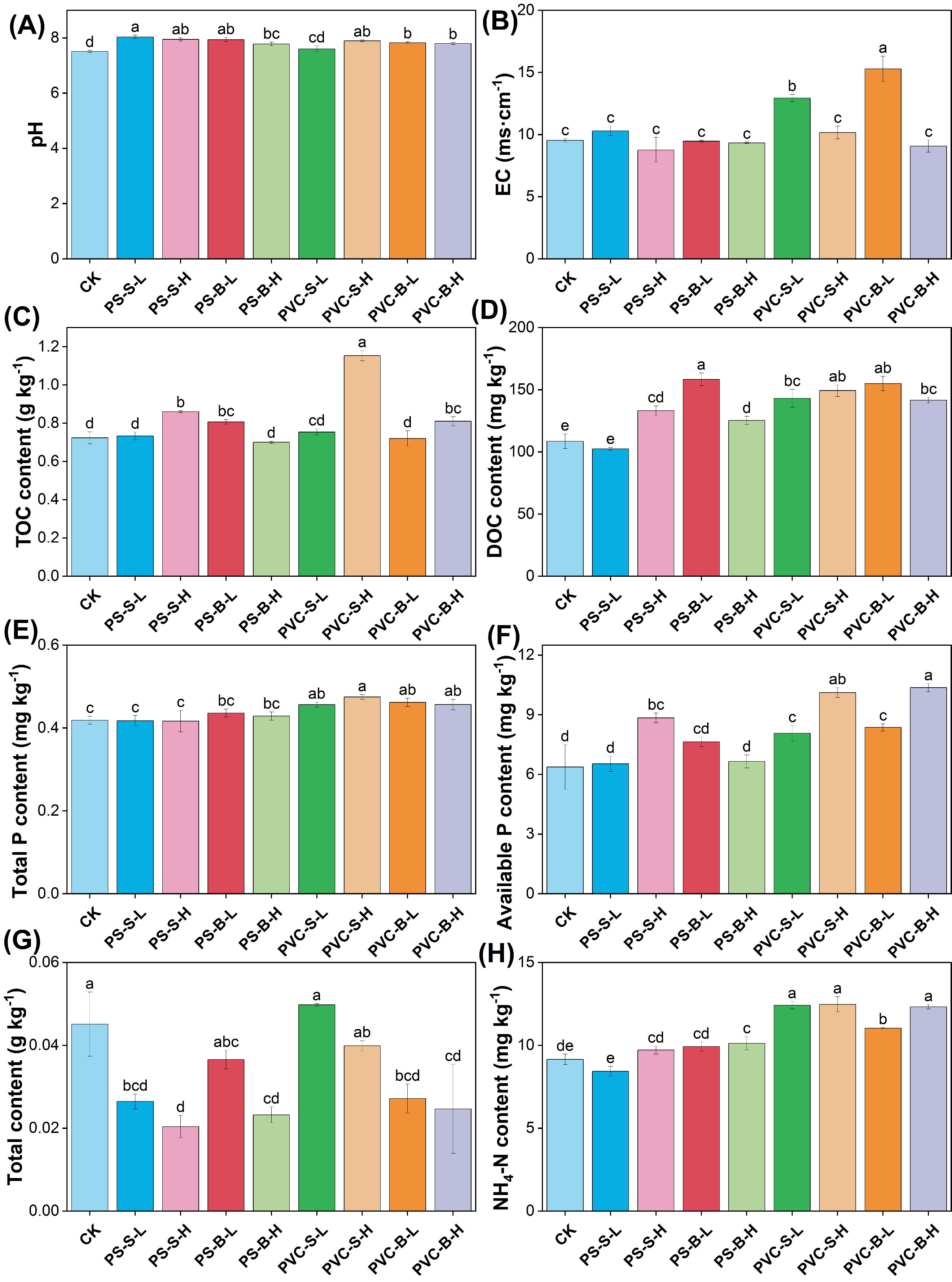


# Fig. S3. Effect of MPs on sediment physicochemical parameters.

# (A) pH, (B)EC, (C) total organic carbon (TOC), (D) DOC, (E) total phosphorus (TP), (F) available phosphorus (AP), (G) total nitrogen (TN) and (H) NH_4_^+^-N. The different small letters reflect a significant difference among the different treatments (Duncan’s multiple-comparison test, *n* = 3, *P* < 0.05).

**Fig. S4.** The concentrations of tetracycline (TC) and sulfamethoxazole (SMX) in (A) sediment overlying seawater and (B) sediment pore water during aging period.

Replenishment of TC to simulate the continuous addition of antibiotics in aquaculture environments. Different small letters on the bars indicate significant differences between different time (Duncan’s multiple-comparison test, *n* = 3, *P* < 0.05).


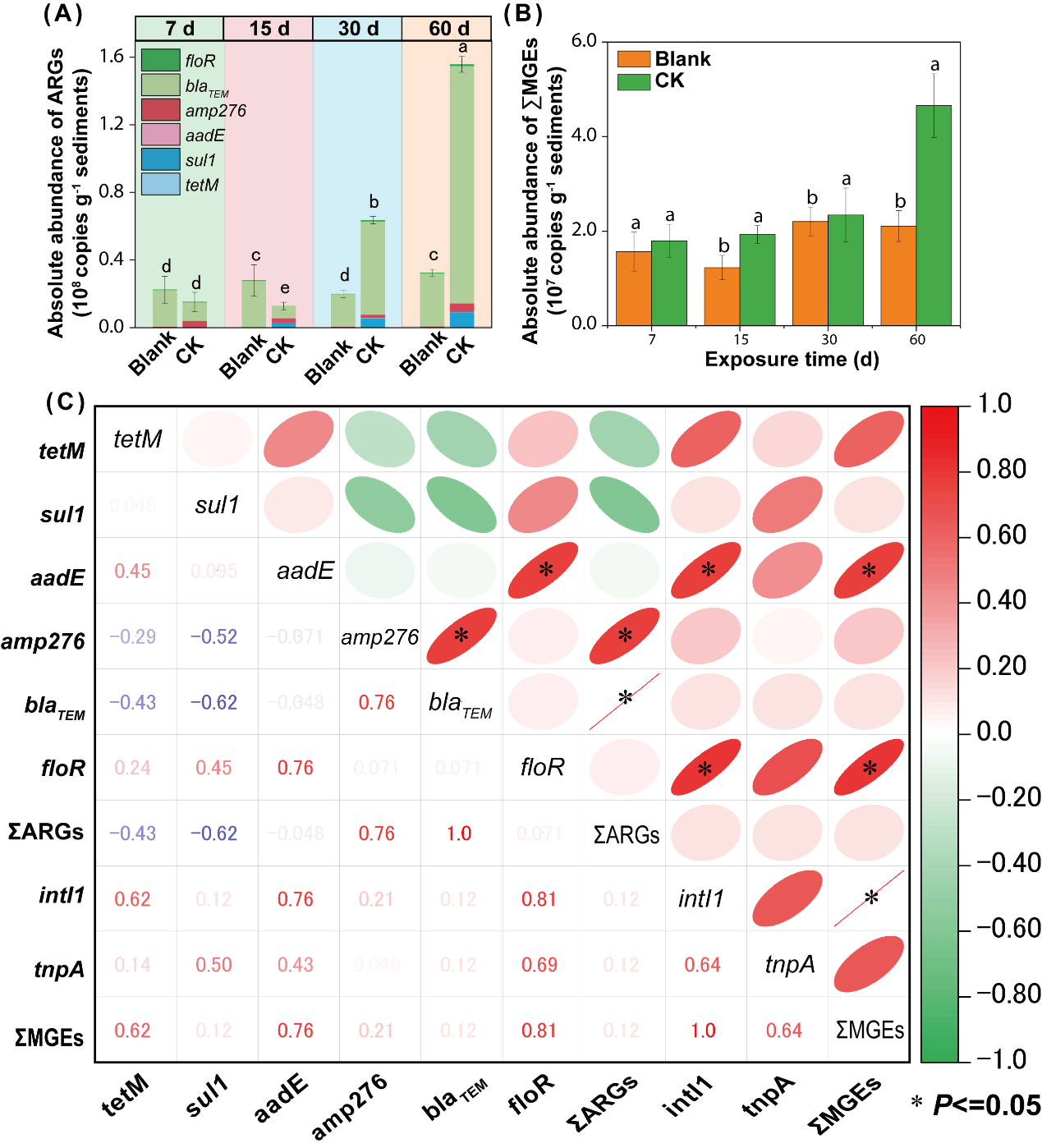


# Fig. S5. Absolute abundance of (A) target six ARGs and (B) two MGEs (*int*I1 and *tnp*A) in sediment at aging phase. (C) Spearman’s correlation heat map of the abundances of target ARGs and MGEs during the antibiotic aging period.

# Blank: No antibiotics added. CK: antibiotics added. The significant levels with *P* ≤ 0.05 are annotated with asterisks (*).


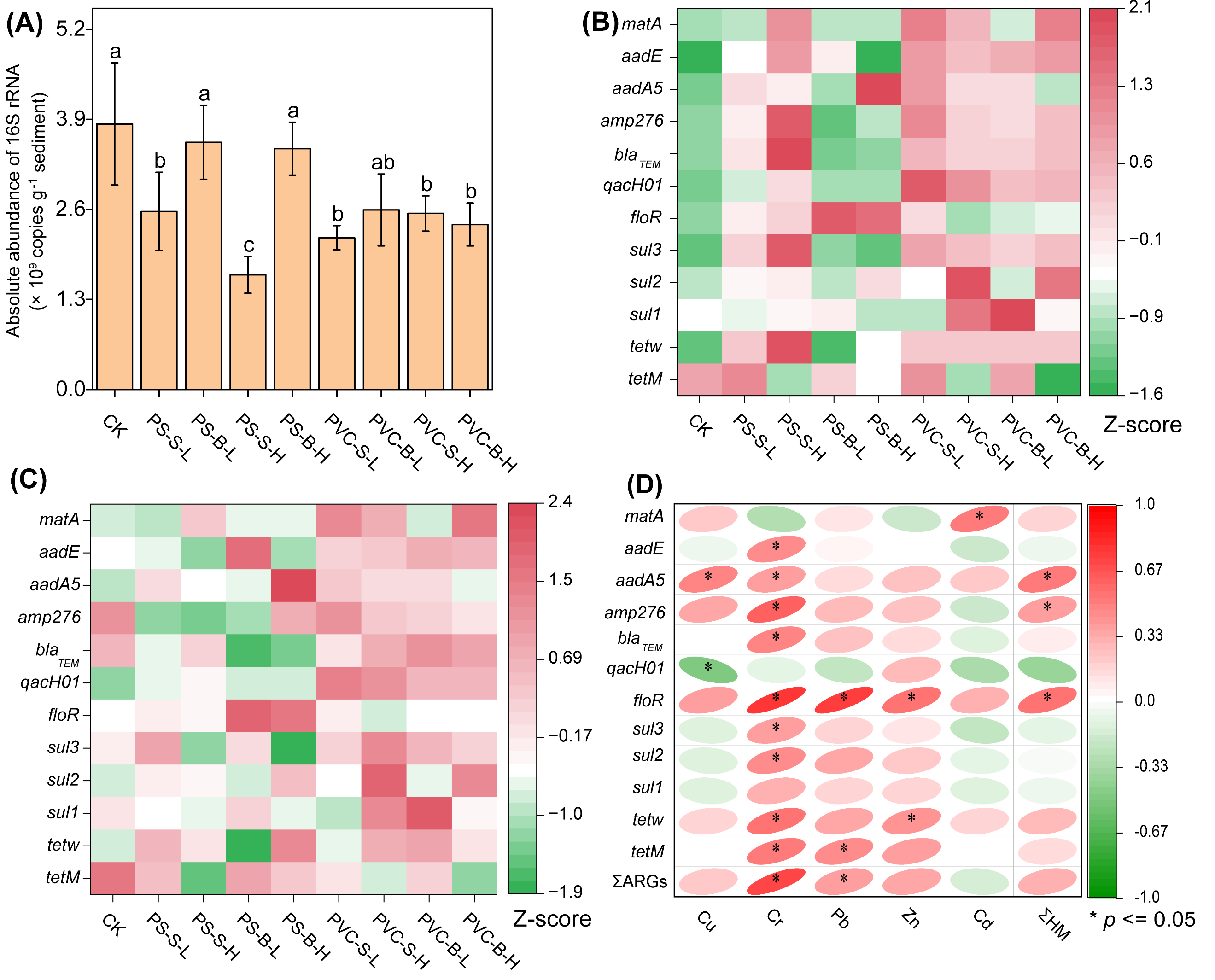


**Fig. S6.** Heat map representation of z-score transformed the absolute and relative abundance of target ARGs in the marine sediments.

(A) Absolute abundance of 16S rRNA Heat map representation of z-score transformed (B) the absolute and (C) relative abundance of target ARGs in the marine sediments polluted by MPs in relative to the control treatment. Higher z-score indicates a higher ARGs abundance. (D) Spearman correlation analysis between the absolute abundance of ARGs and heavy metal content in sediments, with asterisks denoting significant correlations at *p* ≤ 0.05.


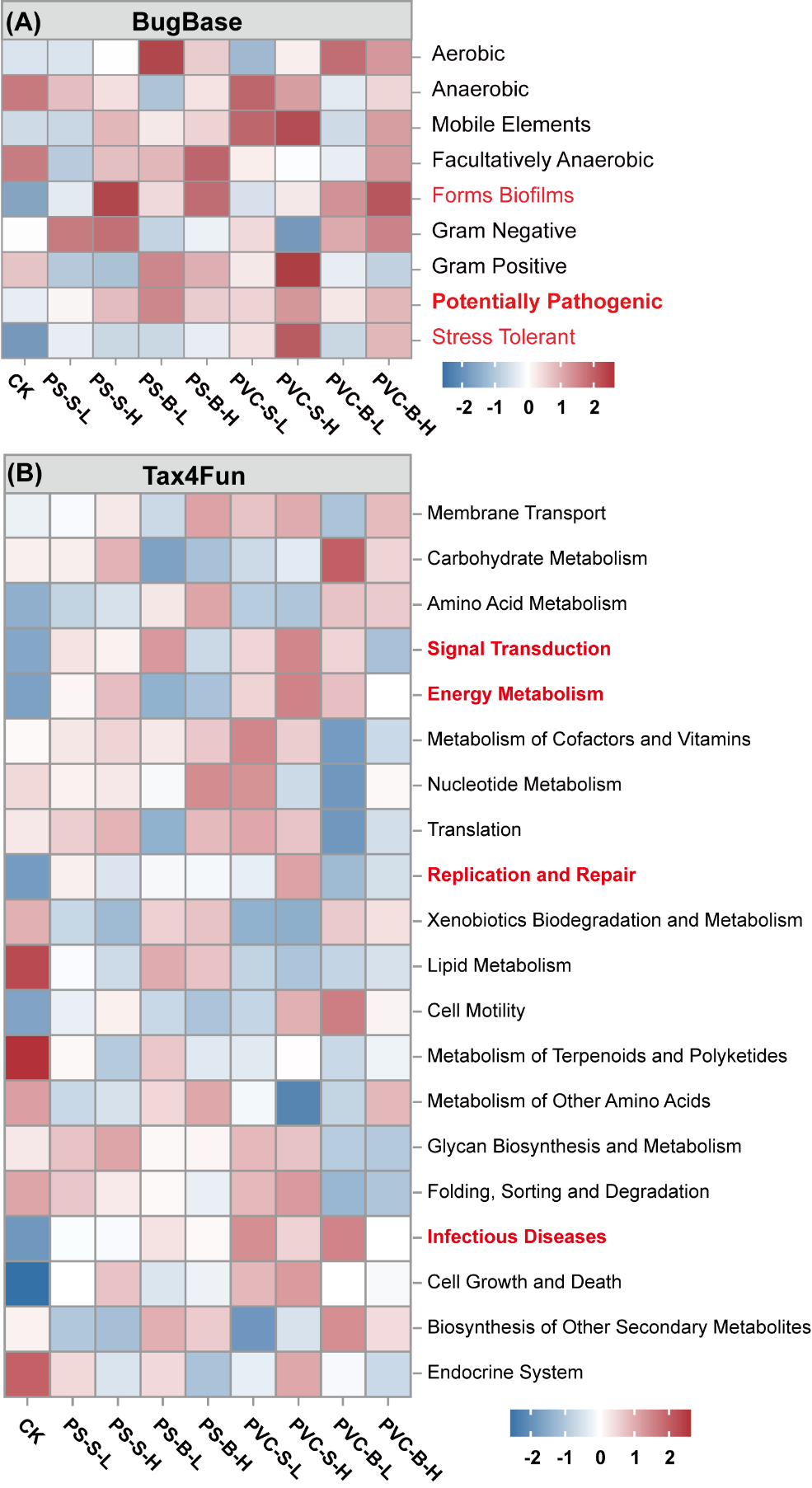


**Fig. S7.** Heatmap analysis of taxonomic to phenotypic mapping of the 16S rRNA sequenced genes in sediments by (A) BugBase and (B) Tax4Fun.

The color code represents the values of min-max normalization of row.


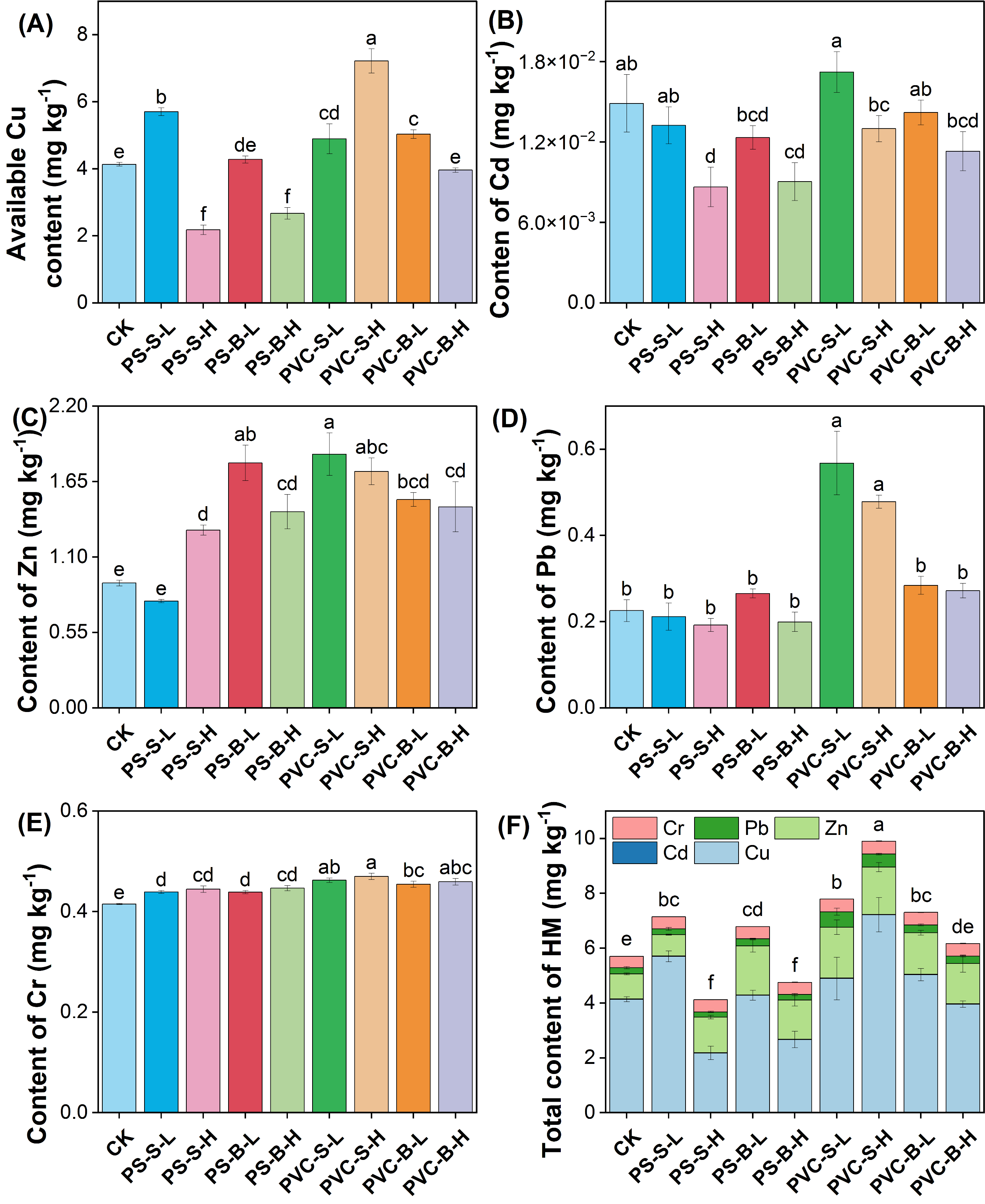


# Fig. S8. Contents of available (a) Cu, (b) Cd, (c) Cr, (d) Pb, (e) Zn and (f) total heavy metal in the sediments.

# Available in sediment. The different small letters reflect a significant difference among the different treatments (Duncan’s multiple-comparison test, *n* = 3, *P* < 0.05).


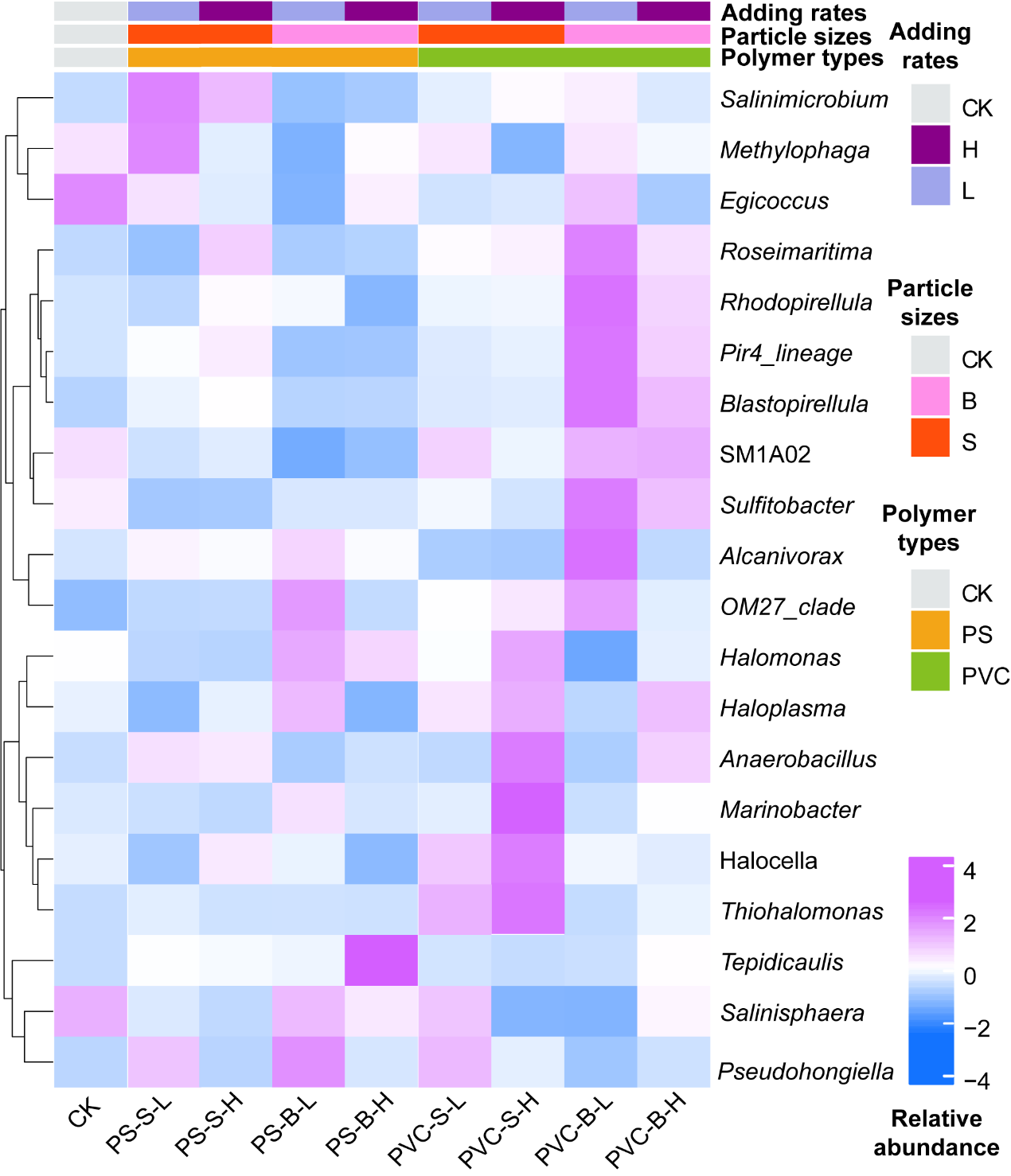


# Fig. S9. Heatmap of the microbial community composition at genus level for taxa with minimum relative abundance of 1% in the sediments polluted by MPs. The color code represents the values of min-max normalization of row.


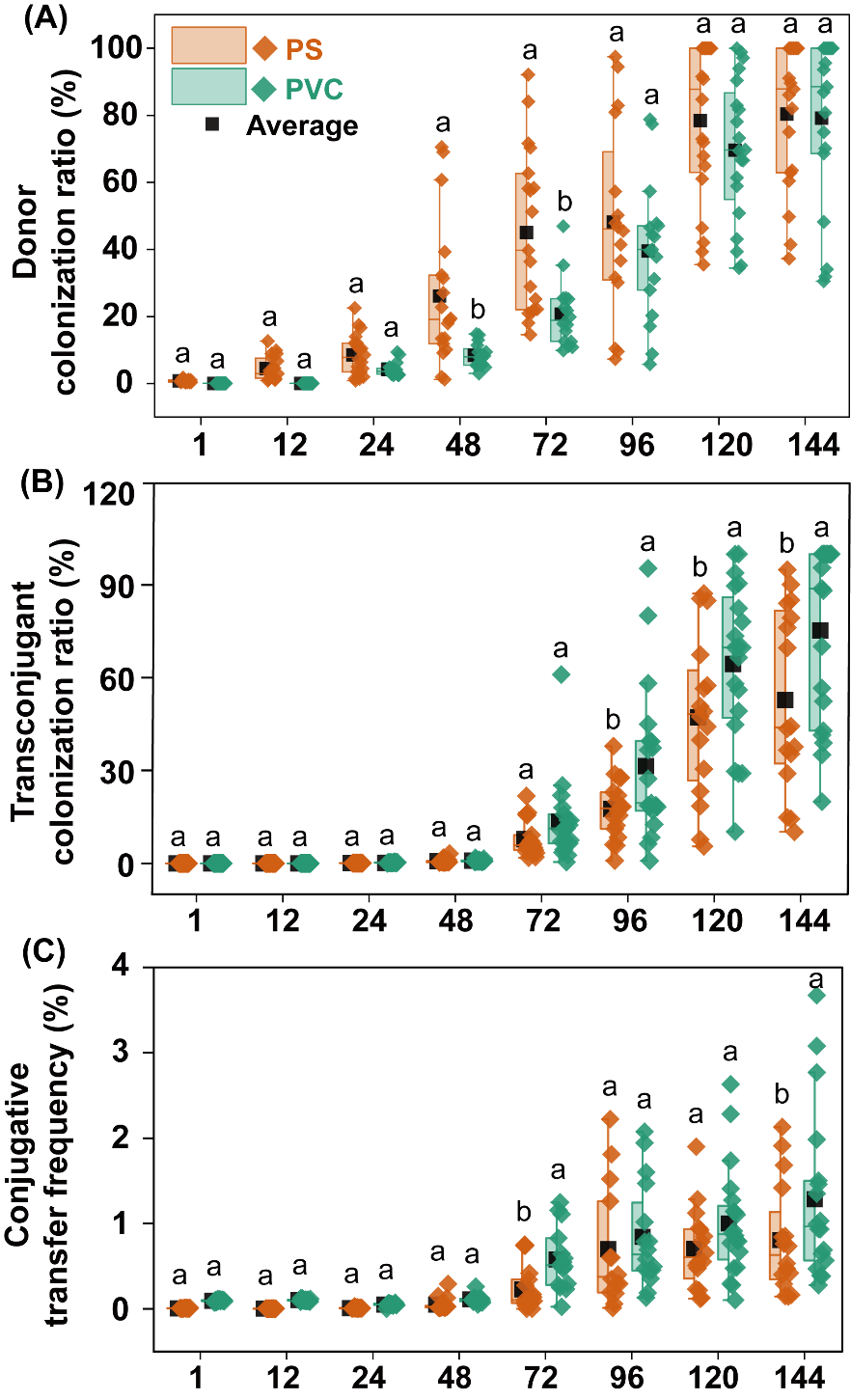


# Fig. S10. Ratio of (A) donor and (B) transconjugant colonization on the MP surfaces.

# (C) Conjugative transfer frequency: the ratio of green fluorescent area to red fluorescent area detected by LSCM and measured with ImageJ/Fiji . Different small letters indicate significant differences between PS and PVC at the same cultivation times (Duncan’s multiple-comparison test, n = 20, P < 0.05). After 1 h of cultivation, red fluorescence appeared on the surface of PS MPs, while only a minimal amount of red fluorescence was observed on PVC MPs, indicating a more rapid colonization of PS by bacteria. By 12–24 h of cultivation, the area of red fluorescence on both types of MPs gradually increased, with the colonization rate on PS MPs (4.09–8.82%) higher than PVC MPs (0–5.40%), suggesting that PS MPs were more conducive to bacterial growth. The colonization rate decelerated between 48 and 96 h, with the colonization rate on PS MPs reaching (42.8 ± 31.0%), higher than that on PVC MPs (39.5 ± 20.4%). By 120–144 h, the surfaces of MPs were entirely covered by bacteria, with a fluorescence ratio of 100%.


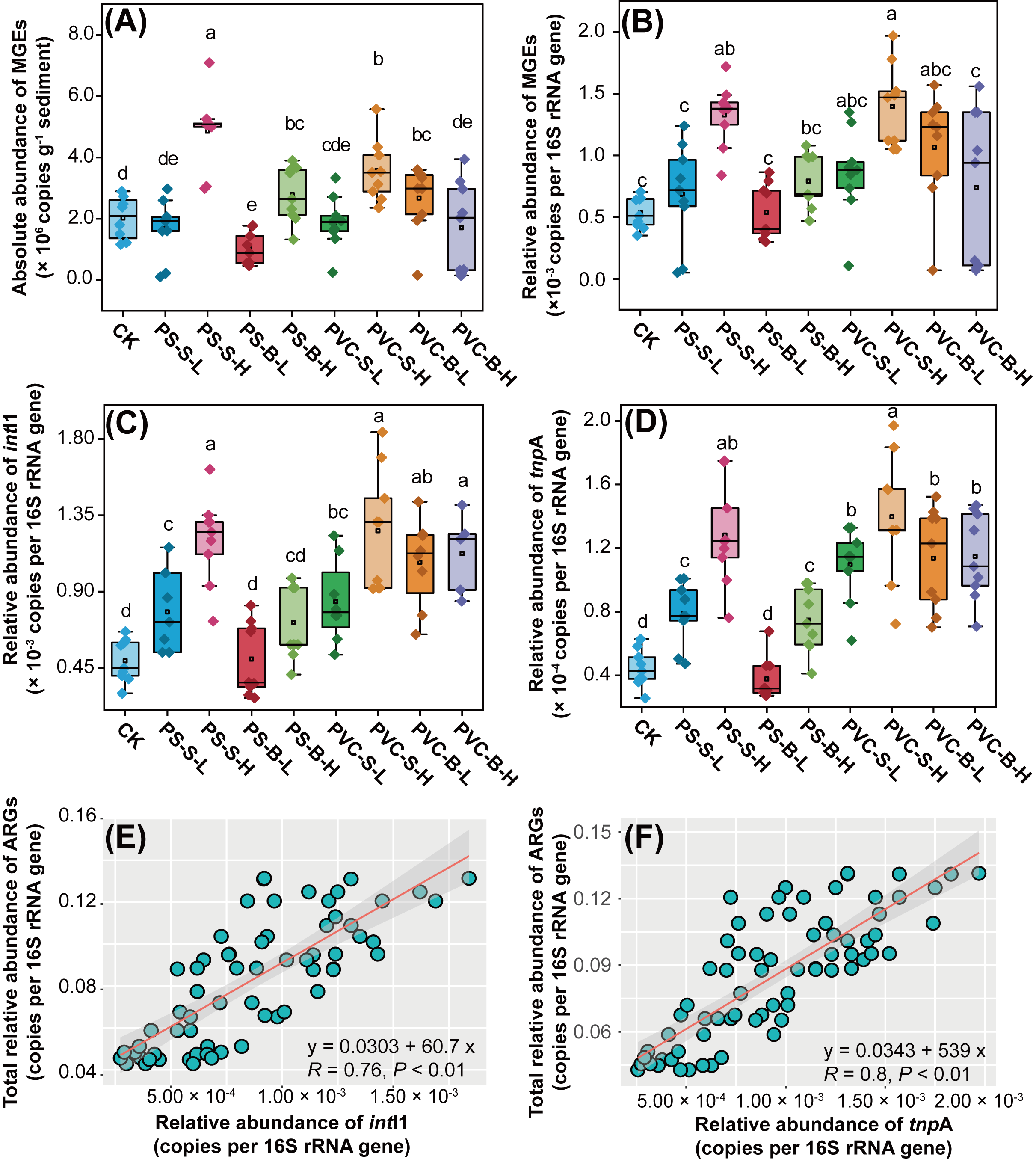


# Fig. S11. The relative abundances of total MGEs, intI1, and tnpA in the marine sediments, and the linear regression between the abundance of ARGs and intI1 and tnpA in the sediments

# The total absolute (A) and relative (B) abundances of the two target MGEs; (C) *int*I1 and (D) *tnp*A in the marine sediments polluted by MPs. Error bars represent the standard deviations of triplicate tests. The different small letters reflect significant difference among the different treatments (Duncan’s multiple-comparison test, *n* = 3, *P* < 0.05). The linear regression between the abundance of ARGs and (E) *intI*1 and (F) *tnp*A in the sediments. The linear relationship fitted a significant correlation (Pearson, *P* < 0.05) and was described using a solid line with 95% confidence interval (gray shades).


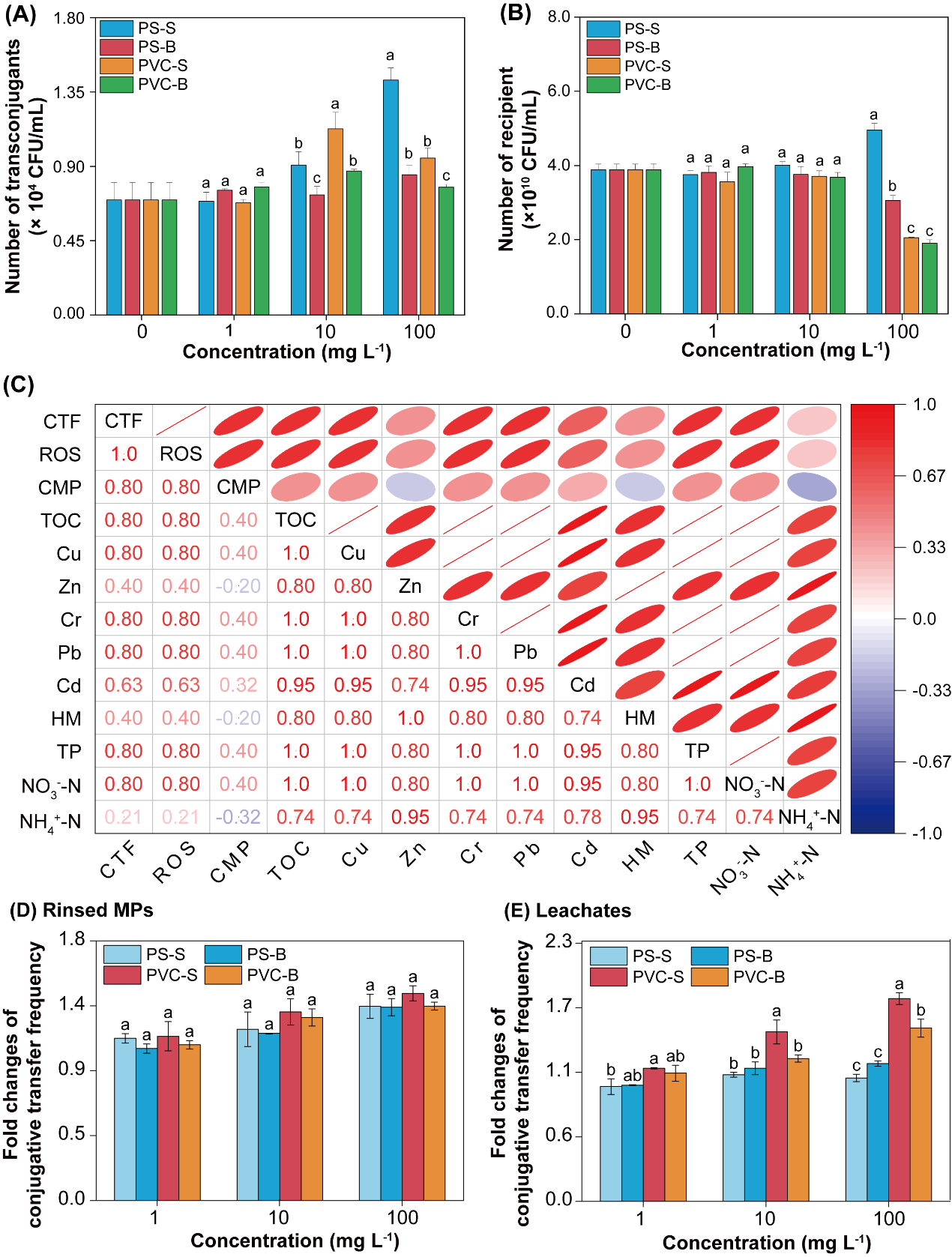


# Fig. S12. Effect of MPs on the conjugative transfer frequency of plasmid RP4 between the donor *P. putida* KT2440 and recipient *E. coli* NK5449 in a 30-mL mating system.

(A) Transconjugant count, (B) recipient *E. coli* NK5449 count. (C) Spearman’s correlation heat map of CTF (conjugative transfer frequency), (CMP) cell membrane permeability, and physicochemical properties of MPs. Amount effect of (D) rinsed MPs and (E) leachates on the conjugative transfer frequency. Error bars represent the standard deviations of triplicate tests. The different small letters reflect a significant difference among the different treatments (Duncan’s multiple-comparison test, *n* = 3, *P* < 0.05).

**Reference**

Barkovskii, A.L., Green, C. and Hurley, D. (2010) The occurrence, spatial and temporal distribution, and environmental routes of tetracycline resistance and integrase genes in Crassostrea virginica beds. *Mar. Pollut. Bull.* 60(12), 2215-2224.

Bliss, C.I. (1939) The toxicity of poisons applied jointly 1. *Ann. Appl. Biol.* 26(3), 585-615.

Brochado, A.R., Telzerow, A., Bobonis, J., et al. (2018) Species-specific activity of antibacterial drug combinations. *Nature* 559(7713), 259-263.

Chen, B., Liang, X., Nie, X., et al. (2015a) The role of class I integrons in the dissemination of sulfonamide resistance genes in the Pearl River and Pearl River Estuary, South China. *J. Hazard. Mater.* 282, 61-67.

Chen, C.Q., Zheng, L., Zhou, J.L., et al. (2017) Persistence and risk of antibiotic residues and antibiotic resistance genes in major mariculture sites in Southeast China. *Sci. Total Environ.* 580, 1175-1184.

Chen, H., Liu, S., Xu, X.-R., et al. (2015b) Antibiotics in the coastal environment of the Hailing Bay region, South China Sea: Spatial distribution, source analysis and ecological risks. *Mar. Pollut. Bull.* 95(1), 365-373.

Chen, K., Liao, C., Li, P., et al. (2022) A compatible interface of wheat straw/polylactic acid composites collaborative constructed using KH570–Nano TiO2. *J. Polym. Environ.* 30(6), 2209-2221.

Chen, L., Li, H., Liu, Y., et al. (2020) Distribution, residue level, sources, and phase partition of antibiotics in surface sediments from the inland river: a case study of the Xiangjiang River, south-central China. *Environ. Sci. Pollut. Res.* 27(2), 2273-2286.

Chen, Q., Gao, Z., Wu, Y., et al. (2023) Insight into chemical features of migrated additives from plastics and associated risks to estuarine ecosystem. *J. Hazard. Mater.* 448, 130861.

Chen, Q., Wang, Q., Zhang, C., et al. (2021) Aging simulation of thin-film plastics in different environments to examine the formation of microplastic. *Water Res.* 202, 117462.

Chu, L., Wang, J., He, S., et al. (2021) Treatment of pharmaceutical wastewater by ionizing radiation: Removal of antibiotics, antimicrobial resistance genes and antimicrobial activity. *J. Hazard. Mater.* 415, 125724.

Dini-Andreote, F., Stegen, J.C., van Elsas, J.D., et al. (2015) Disentangling mechanisms that mediate the balance between stochastic and deterministic processes in microbial succession. *P. Natl. Acad. Sci. USA* 112(11), E1326-E1332.

Dowarah, K. and Devipriya, S.P. (2019) Microplastic prevalence in the beaches of Puducherry, India and its correlation with fishing and tourism/recreational activities. *Mar. Pollut. Bull.* 148, 123-133.

Fang, C., He, Y., Yang, Y., et al. (2023) Laboratory tidal microcosm deciphers responses of sediment archaeal and bacterial communities to microplastic exposure. *J. Hazard. Mater.* 458, 131813.

Gao, Q., Li, Y., Qi, Z., et al. (2018) Diverse and abundant antibiotic resistance genes from mariculture sites of China's coastline. *Sci. Total Environ.* 630, 117-125.

Gu, Y., Tan, X., Cai, X., et al. (2022) Remediation of As and Cd contaminated sediment by biochars: Accompanied with the change of microbial community. *J. Environ. Chem. Eng.* 10(1), 106912.

Gunaalan, K., Fabbri, E. and Capolupo, M. (2020) The hidden threat of plastic leachates: A critical review on their impacts on aquatic organisms. *Water Res.* 184, 116170.

Guo, X.-p., Yang, Y., Lu, D.-p., et al. (2018) Biofilms as a sink for antibiotic resistance genes (ARGs) in the Yangtze Estuary. *Water Res.* 129, 277-286.

Guo, Y., Qiu, T., Gao, M., et al. (2021) Diversity and abundance of antibiotic resistance genes in rhizosphere soil and endophytes of leafy vegetables: Focusing on the effect of the vegetable species. *J. Hazard. Mater.* 415, 125595.

Hämer, J., Gutow, L., Köhler, A., et al. (2014) Fate of microplastics in the marine isopod idotea emarginata. *Environ. Sci. Technol.* 48(22), 13451-13458.

Han, Q.F., Zhao, S., Zhang, X.R., et al. (2020) Distribution, combined pollution and risk assessment of antibiotics in typical marine aquaculture farms surrounding the Yellow Sea, North China. *Environ. Int.* 138, 105551.

Han, Y., Wang, J., Zhao, Z., et al. (2017) Fishmeal application induces antibiotic resistance gene propagation in mariculture sediment. *Environ. Sci. Technol.* 51(18), 10850-10860.

Hardy, O.J. (2008) Testing the spatial phylogenetic structure of local communities: statistical performances of different null models and test statistics on a locally neutral community. *J. Ecol.* 96(5), 914-926.

Hu, J., Lim, F.Y. and Hu, J. (2023) Characteristics and behaviors of microplastics undergoing photoaging and Advanced Oxidation Processes (AOPs) initiated aging. *Water Res.* 232, 119628.

Jang, H.M., Kim, Y.B., Choi, S., et al. (2018) Prevalence of antibiotic resistance genes from effluent of coastal aquaculture, South Korea. *Environ. Pollut.* 233, 1049-1057.

Kaal, J., Goñi-Urtiaga, A., Wenig, P., et al. (2023) Simultaneous molecular fingerprinting of natural organic matter and synthetic polymers (PE, PET, PP, PS and PVC) using analytical pyrolysis. *J. Anal. Appl. Pyrolysis* 175, 106159.

Lassen, S.B., Ahsan, M.E., Islam, S.R., et al. (2022) Prevalence of antibiotic resistance genes in Pangasianodon hypophthalmus and Oreochromis niloticus aquaculture production systems in Bangladesh. *Sci. Total Environ.* 813, 151915.

Le, T.X. and Munekage, Y. (2004) Residues of selected antibiotics in water and mud from shrimp ponds in mangrove areas in Viet Nam. *Mar. Pollut. Bull.* 49(11), 922-929.

Le, T.X., Munekage, Y. and Kato, S.-i. (2005) Antibiotic resistance in bacteria from shrimp farming in mangrove areas. *Sci. Total Environ.* 349(1), 95-105.

Li, B., Qiu, Y., Zhang, J., et al. (2018a) Real-time study of rapid spread of antibiotic resistance plasmid in biofilm using microfluidics. *Environ. Sci. Technol.* 52(19), 11132-11141.

Li, J., Zhang, H., Zhang, K., et al. (2018b) Characterization, source, and retention of microplastic in sandy beaches and mangrove wetlands of the Qinzhou Bay, China. *Mar. Pollut. Bull.* 136, 401-406.

Li, Z., Hu, X., Qin, L., et al. (2020) Evaluating the effect of different modified microplastics on the availability of polycyclic aromatic hydrocarbons. *Water Res.* 170, 115290.

Liao, H., Lu, X., Rensing, C., et al. (2018) Hyperthermophilic composting accelerates the removal of antibiotic resistance genes and mobile genetic elements in sewage sludge. *Environ. Sci. Technol.* 52(1), 266-276.

Lobelle, D. and Cunliffe, M. (2011) Early microbial biofilm formation on marine plastic debris. *Mar. Pollut. Bull.* 62(1), 197-200.

Low, W.W., Wong, J.L.C., Beltran, L.C., et al. (2022) Mating pair stabilization mediates bacterial conjugation species specificity. *Nat. Microbiol.* 7(7), 1016-1027.

Lu, J., Zhang, Y., Wu, J., et al. (2022) Fate of land-based antibiotic resistance genes in marginal-sea sediment: Territorial differentiation and corresponding drivers. *Chemosphere* 288, 132540.

Lu, K., Dong, S., Petersen, E.J., et al. (2017) Biological uptake, distribution, and depuration of radio-labeled graphene in adult zebrafish: Effects of graphene size and natural organic matter. *ACS Nano* 11(3), 2872-2885.

Lu, X.-M. and Lu, P.-Z. (2020) Seasonal variations in antibiotic resistance genes in estuarine sediments and the driving mechanisms. *J. Hazard. Mater.* 383, 121164.

Luo, G., Liang, B., Cui, H., et al. (2023) Determining the contribution of micro/nanoplastics to antimicrobial resistance: challenges and perspectives. *Environ. Sci. Technol.* 57(33), 12137-12152.

Mao, D., Luo, Y., Mathieu, J., et al. (2014) Persistence of extracellular DNA in river sediment facilitates antibiotic resistance gene propagation. *Environ. Sci. Technol.* 48(1), 71-78.

Muziasari, W.I., Pärnänen, K., Johnson, T.A., et al. (2016) Aquaculture changes the profile of antibiotic resistance and mobile genetic element associated genes in Baltic Sea sediments. *FEMS Microbiol. Ecol.* 92(4).

Na, G., Wang, C., Gao, H., et al. (2019) The occurrence of sulfonamide and quinolone resistance genes at the Fildes Peninsula in Antarctica. *Mar. Pollut. Bull.* 149, 110503.

Niu, Z.-G., Zhang, K. and Zhang, Y. (2016) Occurrence and distribution of antibiotic resistance genes in the coastal area of the Bohai Bay, China. *Mar. Pollut. Bull.* 107(1), 245-250.

Paes, E.d.S., Gloaguen, T.V., Silva, H.d.A.d.C., et al. (2022) Widespread microplastic pollution in mangrove soils of Todos os Santos Bay, northern Brazil. *Environ. Res.* 210, 112952.

Peng, Y.-Y., Gao, F., Yang, H.-L., et al. (2020) Simultaneous removal of nutrient and sulfonamides from marine aquaculture wastewater by concentrated and attached cultivation of Chlorella vulgaris in an algal biofilm membrane photobioreactor (BF-MPBR). *Sci. Total Environ.* 725, 138524.

Pu, M., Ailijiang, N., Mamat, A., et al. (2022) Occurrence of antibiotics in the different biological treatment processes, reclaimed wastewater treatment plants and effluent-irrigated soils. *J. Environ. Chem. Eng.* 10(3), 107715.

Qin, Y., Ren, X., Ju, H., et al. (2023) Occurrence and Distribution of Antibiotics in a Tropical Mariculture Area of Hainan, China: Implications for Risk Assessment and Management. *Toxics* 11(5), 421.

Rohart, F., Gautier, B., Singh, A., et al. (2017) mixOmics: An R package for ‘omics feature selection and multiple data integration. *PLoS Comput. Biol.* 13(11), e1005752.

Sagawa, N., Kawaai, K. and Hinata, H. (2018) Abundance and size of microplastics in a coastal sea: Comparison among bottom sediment, beach sediment, and surface water. *Mar. Pollut. Bull.* 133, 532-542.

Seeley, M.E., Song, B., Passie, R., et al. (2020) Microplastics affect sedimentary microbial communities and nitrogen cycling. *Nat. Commun.* 11(1), 2372.

Sheridan, E.A., Fonvielle, J.A., Cottingham, S., et al. (2022) Plastic pollution fosters more microbial growth in lakes than natural organic matter. *Nat. Commun.* 13(1), 4175.

Si, Q., Li, F., Gao, C., et al. (2016) Detection of phthalate esters in seawater by stir bar sorptive extraction and gas chromatography–mass spectrometry. *Mar. Pollut. Bull.* 108(1), 163-170.

Siedlewicz, G., Białk-Bielińska, A., Borecka, M., et al. (2018) Presence, concentrations and risk assessment of selected antibiotic residues in sediments and near-bottom waters collected from the Polish coastal zone in the southern Baltic Sea — Summary of 3years of studies. *Mar. Pollut. Bull.* 129(2), 787-801.

Siedlewicz, G., Pazdro, K., Borecka, M., et al. (2014) Insights on Environmental Changes: Where the World is Heading. Zielinski, T., Pazdro, K., Dragan-Górska, A. and Weydmann, A. (eds), pp. 33-48, Springer International Publishing, Cham.

Sui, Q., Zhang, L., Xia, B., et al. (2020) Spatiotemporal distribution, source identification and inventory of microplastics in surface sediments from Sanggou Bay, China. *Sci. Total Environ.* 723, 138064.

Suzuki, S., Ogo, M., Miller, T., et al. (2013) Who possesses drug resistance genes in the aquatic environment?: sulfamethoxazole (SMX) resistance genes among the bacterial community in water environment of Metro-Manila, Philippines. *Front. Microbiol.* 4.

Szekeres, E., Baricz, A., Chiriac, C.M., et al. (2017) Abundance of antibiotics, antibiotic resistance genes and bacterial community composition in wastewater effluents from different Romanian hospitals. *Environ. Pollut.* 225, 304-315.

Tamminen, M., Karkman, A., Lõhmus, A., et al. (2011) Tetracycline resistance genes persist at aquaculture farms in the absence of selection pressure. *Environ. Sci. Technol.* 45(2), 386-391.

Tan, L., Li, L., Ashbolt, N., et al. (2018) Arctic antibiotic resistance gene contamination, a result of anthropogenic activities and natural origin. *Sci. Total Environ.* 621, 1176-1184.

Thongsamer, T., Neamchan, R., Blackburn, A., et al. (2021) Environmental antimicrobial resistance is associated with faecal pollution in Central Thailand’s coastal aquaculture region. *J. Hazard. Mater.* 416, 125718.

Ur Razzaq, A., McEachern, D.J., Rupar, P.A., et al. (2023) Upcycled polyvinyl chloride (PVC) electrospun nanofibers from waste PVC-based materials for water treatment. *ACS Appl. Eng. Mater.* 1(7), 1924-1936.

Wang, C., Zhao, Y., Liu, S., et al. (2021) Contamination, distribution, and risk assessment of antibiotics in the urban surface water of the Pearl River in Guangzhou, South China. *Environmental Monitoring and Assessment* 193(2), 98.

Wang, H., Yu, P., Schwarz, C., et al. (2022a) Phthalate esters released from plastics promote biofilm formation and chlorine resistance. *Environ. Sci. Technol.* 56(2), 1081-1090.

Wang, P.-Y., Zhao, Z.-Y., Xiong, X.-B., et al. (2023a) Microplastics affect soil bacterial community assembly more by their shapes rather than the concentrations. *Water Res.* 245, 120581.

Wang, Q., Tan, L., Sun, S., et al. (2023b) Land-derived wastewater facilitates antibiotic resistance contamination in marine sediment of semi-closed bay: A case study in Jiaozhou Bay, China. *J. Environ. Manage.* 339, 117870.

Wang, Q., Wangjin, X., Zhang, Y., et al. (2020a) The toxicity of virgin and UV-aged PVC microplastics on the growth of freshwater algae Chlamydomonas reinhardtii. *Sci. Total Environ.* 749, 141603.

Wang, T., Hu, M., Song, L., et al. (2020b) Coastal zone use influences the spatial distribution of microplastics in Hangzhou Bay, China. *Environ. Pollut.* 266, 115137.

Wang, X., Lin, Y., Zheng, Y., et al. (2022b) Antibiotics in mariculture systems: A review of occurrence, environmental behavior, and ecological effects. *Environ. Pollut.* 293, 118541.

Wang, X., Zheng, H., Zhao, J., et al. (2020c) Photodegradation elevated the toxicity of polystyrene microplastics to grouper (Epinephelus moara) through disrupting hepatic lipid homeostasis. *Environ. Sci. Technol.* 54(10), 6202-6212.

Wang, Y., Lu, J., Mao, L., et al. (2019) Antiepileptic drug carbamazepine promotes horizontal transfer of plasmid-borne multi-antibiotic resistance genes within and across bacterial genera. *ISME J.* 13(2), 509-522.

Wei, Y., Zhang, Y., Xu, J., et al. (2014) Simultaneous quantification of several classes of antibiotics in water, sediments, and fish muscles by liquid chromatography-tandem mass spectrometry. *Front. Environ. Sci. Eng.* 8(3), 357-371.

Wu, J., Zhou, J., Liu, D., et al. (2023) Phthalates promote dissemination of antibiotic resistance genes: An overlooked environmental risk. *Environ. Sci. Technol.* 57(17), 6876-6887.

Xu, C., Lu, J., Shen, C., et al. (2022a) Deciphering the mechanisms shaping the plastisphere antibiotic resistome on riverine microplastics. *Water Res.* 225, 119192.

Xu, L., Wang, T., Wang, J., et al. (2017) Occurrence, speciation and transportation of heavy metals in 9 coastal rivers from watershed of Laizhou Bay, China. *Chemosphere* 173, 61-68.

Xu, M., Huang, X.-h., Shen, X.-x., et al. (2022b) Metagenomic insights into the spatiotemporal responses of antibiotic resistance genes and microbial communities in aquaculture sediments. *Chemosphere* 307, 135596.

Xue, B., Zhang, L., Li, R., et al. (2020) Underestimated microplastic pollution derived from fishery activities and “hidden” in deep sediment. *Environ. Sci. Technol.* 54(4), 2210-2217.

Yang, H., Liu, R., Liu, H., et al. (2021) Evidence for long-term anthropogenic pollution: The hadal trench as a depository and indicator for dissemination of antibiotic resistance genes. *Environ. Sci. Technol.* 55(22), 15136-15148.

Yang, H., Xu, M., Wang, L., et al. (2023) Metagenomic analysis to determine the characteristics of antibiotic resistance genes in typical antibiotic-contaminated sediments. *J. Environ. Sci.* 128, 12-25.

Yu, Y., Chen, L., fang, Y., et al. (2019) High temperatures can effectively degrade residual tetracyclines in chicken manure through composting. *J. Hazard. Mater.* 380, 120862.

Yu, Z., Wang, Y., Lu, J., et al. (2021) Nonnutritive sweeteners can promote the dissemination of antibiotic resistance through conjugative gene transfer. *ISME J.* 15(7), 2117-2130.

Yuan, Q., Sun, R., Yu, P., et al. (2022) UV-aging of microplastics increases proximal ARG donor-recipient adsorption and leaching of chemicals that synergistically enhance antibiotic resistance propagation. *J. Hazard. Mater.* 427, 127895.

Zeng, Q., Xiang, J., Yang, C., et al. (2023) Microplastics affect nitrogen cycling and antibiotic resistance genes transfer of sediment. *Chem. Eng. J.* 454, 140193.

Zhang, Y., Lu, J., Wu, J., et al. (2020) Occurrence and distribution of antibiotic resistance genes in sediments in a semi-enclosed continental shelf sea. *Sci. Total Environ.* 720, 137712.

Zhang, Z., Peng, H., Zhang, J., et al. (2023) Pollution characteristics of typical ARGs in the sediments of the sea area adjacent to the Yangtze Estuary, China. *Environ. Pollut.* 316, 120470.

Zhao, M., Ma, X., Liao, X., et al. (2022) Characteristics of algae-derived biochars and their sorption and remediation performance for sulfamethoxazole in marine environment. *Chem. Eng. J.* 430, 133092.

Zheng, H., Feng, N., Yang, T., et al. (2021) Individual and combined applications of biochar and pyroligneous acid mitigate dissemination of antibiotic resistance genes in agricultural soil. *Sci. Total Environ.* 796, 148962.

Zheng, H., Wang, Z., Zhao, J., et al. (2013) Sorption of antibiotic sulfamethoxazole varies with biochars produced at different temperatures. *Environ. Pollut.* 181, 60-67.

Zhou, J. and Ning, D. (2017) Stochastic community assembly: Does it matter in microbial ecology? *Microbiol. Mol. Biol. Rev.* 81(4), e00002-e00017.

Zhu, D., Ma, J., Li, G., et al. (2022) Soil plastispheres as hotspots of antibiotic resistance genes and potential pathogens. *ISME J.* 16(2), 521-532.

Zhu, Y.-G., Johnson, T.A., Su, J.-Q., et al. (2013) Diverse and abundant antibiotic resistance genes in Chinese swine farms. *P. Natl. Acad. Sci. USA* 110(9), 3435-3440.

Zhu, Y., Zhao, Y., Li, B., et al. (2017) Continental-scale pollution of estuaries with antibiotic resistance genes. *Nat. Microbiol.* 2(4), 16270.
